# Global, regional, and national burden of suicide mortality 1990 to 2016: systematic analysis for the Global Burden of Disease Study 2016

**DOI:** 10.1136/bmj.l94

**Published:** 2019-02-06

**Authors:** Mohsen Naghavi, Global Burden of Disease Self-Harm Collaborators, Heather M Orpana, Laurie B Marczak, Megha Arora, Nooshin Abbasi, Rizwan Suliankatchi Abdulkader, Zegeye Abebe, Haftom Niguse Abraha, Mohsen Afarideh, Mahdi Afshari, Alireza Ahmadi, Amani Nidhal Aichour, Ibtihel Aichour, Miloud Taki Eddine Aichour, Nadia Akseer, Rajaa M Al-Raddadi, Fares Alahdab, Ala’a Alkerwi, Peter Allebeck, Nelson Alvis-Guzman, Nahla Hamed Anber, Mina Anjomshoa, Carl Abelardo T Antonio, Amit Arora, Krishna K Aryal, Solomon Weldegebreal Asgedom, Ashish Awasthi, Beatriz Paulina Ayala Quintanilla, Hamid Badali, Suzanne Lyn Barker-Collo, Till Winfried Bärnighausen, Shahrzad Bazargan-Hejazi, Corina Benjet, Isabela M Bensenor, Noami Berfeld, Mircea Beuran, Zulfiqar A Bhutta, Belete Biadgo, Nigus Bililign, Guilherme Borges, Rohan Borschmann, Alexandra Brazinova, Nicholas J K Breitborde, Traolach Brugha, Zahid A Butt, Juan J Carrero, Félix Carvalho, Deborah Carvalho Malta, Carlos A Castañeda-Orjuela, Ferrán Catalá-López, Liliana G Ciobanu, Berihun Assefa Dachew, Lalit Dandona, Rakhi Dandona, Paul I Dargan, Ahmad Daryani, Dragos Virgil Davitoiu, Kairat Davletov, Louisa Degenhardt, Gebre Teklemariam Demoz, Don C Des Jarlais, Samath Dhamminda Dharmaratne, Shirin Djalalinia, Linh Doan, David Teye Doku, Manisha Dubey, Ziad El-Khatib, Sharareh Eskandarieh, Alireza Esteghamati, Sadaf Esteghamati, Andre Faro, Farshad Farzadfar, Wubalem Fekadu, Eduarda Fernandes, Alize J Ferrari, Irina Filip, Florian Fischer, Kyle J Foreman, Takeshi Fukumoto, Abadi Kahsu Gebre, Giuseppe Grosso, Rahul Gupta, Juanita A Haagsma, Hassan Haghparast Bidgoli, Arvin Haj-Mirzaian, Samer Hamidi, Graeme J Hankey, Josep Maria Haro, Hamid Yimam Hassen, Simon I Hay, Behnam Heidari, Delia Hendrie, Enayatollah Homaie Rad, Seyed Mostafa Hosseini, Sorin Hostiuc, Seyed Sina Naghibi Irvani, Sheikh Mohammed Shariful Islam, Mihajlo Jakovljevic, Spencer James, Achala Upendra Jayatilleke, Ravi Prakash Jha, Jost B Jonas, Jacek Jerzy Jozwiak, Rajendra Kadel, Amaha Kahsay, Amir Kasaeian, Getachew Mullu Kassa, Norito Kawakami, Adane Teshome Kefale, Grant Rodgers Kemp, Yousef Saleh Khader, Morteza Abdullatif Khafaie, Ibrahim A Khalil, Ejaz Ahmad Khan, Muhammad Ali Khan, Muhammad Shahzeb Khan, Young-Ho Khang, Jagdish Khubchandani, Aliasghar A Kiadaliri, Christian Kieling, Young-Eun Kim, Adnan Kisa, Ann Kristin Skrindo Knudsen, Yoshihiro Kokubo, Ai Koyanagi, Varsha Sarah Krish, Barthelemy Kuate Defo, G Anil Kumar, Manasi Kumar, Prabhat Lamichhane, Justin J Lang, Arman Latifi, Paul H Lee, Janni Leung, Lee-Ling Lim, Alan D Lopez, Stefan Lorkowski, Paulo A Lotufo, Rafael Lozano, Raimundas Lunevicius, P A Mahesh, Marek Majdan, Reza Majdzadeh, Reza Malekzadeh, Ana-Laura Manda, Mohammad Ali Mansournia, Lorenzo Giovanni Mantovani, Joemer C Maravilla, Jose Martinez-Raga, Manu Raj Mathur, Pallab K Maulik, John J McGrath, Ravi Mehrotra, Tesfa Mekonen, Walter Mendoza, Tuomo J Meretoja, Tomislav Mestrovic, Ted R Miller, GK Mini, Erkin M Mirrakhimov, Philip B Mitchell, Babak Moazen, Karzan Abdulmuhsin Mohammad, Moslem Mohammadi, Shafiu Mohammed, Ali H Mokdad, Lorenzo Monasta, Mahmood Moosazadeh, Ghobad Moradi, Maziar Moradi-Lakeh, Mehdi Moradinazar, Ilais Moreno Velásquez, Naho Morisaki, Shane Douglas Morrison, Marilita M Moschos, Seyyed Meysam Mousavi, Ghulam Mustafa, Gabriele Nagel, Aliya Naheed, Gurudatta Naik, Farid Najafi, Ionut Negoi, Ruxandra Irina Negoi, Huong Lan Thi Nguyen, Long Hoang Nguyen, Molly R Nixon, Richard Ofori-Asenso, Felix Akpojene Ogbo, In-Hwan Oh, Andrew T Olagunju, Tinuke O Olagunju, Simon Øverland, Mayowa Ojo Owolabi, Songhomitra Panda-Jonas, Charles D H Parry, Sanghamitra Pati, Scott B Patten, George C Patton, Max Petzold, Michael R Phillips, Oleguer Plana-Ripoll, Maarten J Postma, Akram Pourshams, Hossein Poustchi, Mostafa Qorbani, Amir Radfar, Anwar Rafay, Alireza Rafiei, Fakher Rahim, Afarin Rahimi-Movaghar, Vafa Rahimi-Movaghar, Muhammad Aziz Rahman, Rajesh Kumar Rai, Shahab Rezaeian, Leonardo Roever, Luca Ronfani, Gholamreza Roshandel, Ali Rostami, Perminder S Sachdev, Hosein Safari, Saeid Safiri, Payman Salamati, Yahya Salimi, Joshua A Salomon, Abdallah M Samy, Itamar S Santos, Milena M Santric-Milicevic, Benn Sartorius, Shahabeddin Sarvi, Maheswar Satpathy, Monika Sawhney, David C Schwebel, Sadaf G Sepanlou, Masood Ali Shaikh, Mehdi Sharif, Kenji Shibuya, Mika Shigematsu, Rahman Shiri, Ivy Shiue, Soraya Siabani, Tariq J Siddiqi, Inga Dora Sigfusdottir, João Pedro Silva, Jasvinder A Singh, Adauto Martins Soares Filho, Soheila Sobhani, Dan J Stein, Murray B Stein, Mu’awiyyah Babale Sufiyan, Bruno F Sunguya, Rafael Tabarés-Seisdedos, Karen M Tabb, Mohammad Tavakkoli, Arash Tehrani-Banihashemi, Mohamad-Hani Temsah, Roman Topor-Madry, Bach Xuan Tran, Khanh Bao Tran, Irfan Ullah, Jurgen Unutzer, Muhammad Shariq Usman, Olalekan A Uthman, Pascual R Valdez, Tommi Juhani Vasankari, Cintia Vasconcelos, Vasily Vlassov, Theo Vos, Isidora S Vujcic, Yasir Waheed, Yuan-Pang Wang, Elisabete Weiderpass, Andrea Werdecker, Ronny Westerman, Harvey A Whiteford, Grant M A Wyper, Mehdi Yaseri, Ebrahim M Yimer, Engida Yisma, Naohiro Yonemoto, Seok-Jun Yoon, Marcel Yotebieng, Mahmoud Yousefifard, Chuanhua Yu, Zoubida Zaidi, Mohammad Zamani, Christopher J L Murray, Mohsen Naghavi

**Affiliations:** 1 Institute for Health Metrics and Evaluation, University of Washington, Seattle, WA 98121, USA

## Abstract

**Objectives:**

To use the estimates from the Global Burden of Disease Study 2016 to describe patterns of suicide mortality globally, regionally, and for 195 countries and territories by age, sex, and Socio-demographic index, and to describe temporal trends between 1990 and 2016.

**Design:**

Systematic analysis.

**Main outcome measures:**

Crude and age standardised rates from suicide mortality and years of life lost were compared across regions and countries, and by age, sex, and Socio-demographic index (a composite measure of fertility, income, and education).

**Results:**

The total number of deaths from suicide increased by 6.7% (95% uncertainty interval 0.4% to 15.6%) globally over the 27 year study period to 817 000 (762 000 to 884 000) deaths in 2016. However, the age standardised mortality rate for suicide decreased by 32.7% (27.2% to 36.6%) worldwide between 1990 and 2016, similar to the decline in the global age standardised mortality rate of 30.6%. Suicide was the leading cause of age standardised years of life lost in the Global Burden of Disease region of high income Asia Pacific and was among the top 10 leading causes in eastern Europe, central Europe, western Europe, central Asia, Australasia, southern Latin America, and high income North America. Rates for men were higher than for women across regions, countries, and age groups, except for the 15 to 19 age group. There was variation in the female to male ratio, with higher ratios at lower levels of Socio-demographic index. Women experienced greater decreases in mortality rates (49.0%, 95% uncertainty interval 42.6% to 54.6%) than men (23.8%, 15.6% to 32.7%).

**Conclusions:**

Age standardised mortality rates for suicide have greatly reduced since 1990, but suicide remains an important contributor to mortality worldwide. Suicide mortality was variable across locations, between sexes, and between age groups. Suicide prevention strategies can be targeted towards vulnerable populations if they are informed by variations in mortality rates.

## Introduction

Suicide is recognised as a critical public health issue by the World Health Organization in its Comprehensive Mental Health Action Plan.^1^ The plan contains a target to decrease global suicide mortality by 10% between 2012 and 2020. Proposed actions for WHO Member States include the development and implementation of comprehensive national suicide prevention strategies, with a focus on populations identified as at increased risk for suicide. As of January 2018, 28 of 194 WHO Member States report having a national suicide prevention strategy.^2^ Suicide mortality is also an indicator for Sustainable Development Goal 3.4.2, to be used to measure progress towards a targeted one third reduction in premature mortality from non-communicable diseases through enhanced prevention, treatment, and promotion of mental health and well being.^3^

Suicide is defined as a death caused by intentional self directed injury.^4^ It varies systematically by age, sex, and means of suicide.^5^ ^6^ A complex web of factors underlies suicide mortality, including both risk factors and protective factors at individual, family, community, and societal levels 7 8 9 10; choices of and access to means of suicide 11 12 13; and mental illness and access to mental healthcare and other services.^14^ ^15^ ^16^ ^17^ These drivers of suicide mortality also vary systematically by region. For example, in most parts of the world, suicide deaths are higher among men than women,^5^ although this ratio is much lower for countries across a belt that extends from southern India to China, including some islands in the Pacific ocean.^5^ ^18^ ^19^ In Western countries, there is a strong relation between mental illness and suicide,^20^ however, in Asia, this relation is much less pronounced.^21^

Given the focus on preventing suicide internationally and by specific countries, an accounting of levels and trends of suicide mortality, including analyses by region, country, age, and sex is necessary to inform suicide prevention efforts. However, suicide is stigmatised and, in some countries, illegal, which can lead to misclassification and variability in data quality.^5^ ^22^ The methods of the Global Burden of Disease Study 2016,^23^ which address some of this misclassification through data processing, provide an opportunity to present internationally comparable estimates of suicide that account for some of these data issues. Details on how misclassification is handled are outlined in the Global Burden of Disease Study 2016 Causes of Death appendix.^23^

The standardised methods and comprehensive data of the Global Burden of Disease Study 2016 facilitate an assessment of the patterns of suicide mortality in 2016 by age, sex, and location, and allow comparisons across the period of 1990 to 2016. The data and methods in the Global Burden of Disease Study 2016 represent substantive improvements to previous iterations of the Global Burden of Disease Study including additional data sources, advances in modelling strategies, and the refinement of a continuous scale (the Socio-demographic index) to explore levels and trends relative to socioeconomic factors between locations. This paper uses estimates from the Global Burden of Disease Study 2016 to describe patterns of suicide mortality globally, regionally, and for 195 countries and territories, by age, sex, and Socio-demographic index, from 1990 to 2016.

## Methods

### Overview

The Global Burden of Disease Study 2016 includes estimates of mortality owing to 264 causes by location, age, and sex between 1990 and 2016 for 195 countries and territories. We provide an overview of the methods employed by the Global Burden of Disease Study 2016 with details specific to the estimation of suicide mortality. The Global Burden of Disease Study 2016 used the ICD (international classification of diseases) definition of suicide mortality as death caused by purposely self-inflicted poisoning or injury (ICD-10 codes X60-X64.9, X66-X84.9, Y87.0; ICD-9 codes E950-E959). For this analysis we present results for suicide as an aggregate cause of death. Additional detailed results can be explored by using online data visualisation tools or downloading a results query tool. Our analyses adhere to the Guidelines for Accurate and Transparent Health Estimates Reporting (GATHER).^24^ The Global Burden of Disease Study 2016 uses de-identified, aggregated data, therefore a waiver of informed consent was reviewed and approved by the University of Washington Institutional Review Board (application number 46665).

### Causes of death data cleaning and formatting

We obtained cause of death data coded as suicide from vital registration systems and verbal autopsy reports. Vital registration systems collect information about births and deaths in a population, and the vital registration data used for our analysis contains the most detailed cause of death whenever possible by using ICD coding systems. We consider vital registration data to be of the highest quality, and use it whenever possible. 

Verbal autopsy is a method of classifying deaths in locations without comprehensive vital registration systems. Verbal autopsy uses trained interviewers to collect information about signs, symptoms and sociodemographic data of the deceased, which are then used to infer a cause of death. 

The database developed for the Global Burden of Disease Study 2016 contains 15 937 location years of vital registration data and 1619 location years of data for suicide. The Global Burden of Disease Study 2016 estimates the period reflecting the initial Global Burden of Disease Study, which estimated disease burden for 1990 commissioned by the World Bank in 1993, to the previous calendar year. Our cause of death estimation extends back to 1980, but in developing countries, data are sparser for these locations before 1990, so we restricted cause-specific analyses to 1990 to 2016. Estimates for 2016 are currently available, and estimates for 2017 will be available in late 2018. We developed a star-rating system to capture the quality of data available for each country over the time series for Global Burden of Disease estimation. The rating for each location is available in supplementary table 1. The rating is dependent on availability, completeness, and detail of data and the percentage of deaths that are ill-defined garbage codes or assigned to highly aggregated causes. We consider extensive vital registration data (ie, vital registration data that is available and complete for the Global Burden of Disease estimation time series) to be high quality. Additional details on the calculation of the rating by using the components can be found in the Global Burden of Disease Study 2016 Causes of Death appendix. We included all data in the models to enhance predictive validity for estimates in countries without extensive vital registration data. We excluded data from countries without extensive vital registration coverage from models to avoid inflation of uncertainty. We accessed the details of the specific data source by location used in the estimation process through the Global Burden of Disease data tool. Supplementary table 2 shows how many verbal autopsy, vital registration, and other sources were used for suicide estimation for each location.

The Global Burden of Disease Study 2016 addressed variation in data quality through a series of methods that include data standardisation and the redistribution of inappropriately coded deaths or “garbage codes” that are not possible causes of death, or that are not specific underlying causes of death, that have been entered as the underlying cause of death on death certificates.^23^ Undercounting or wrong assignment of deaths from suicide is a known problem in suicide death estimation, and the level and type of wrong assignment differs by location, age, sex, and time.^22^ ^25^ Wrong assignment is corrected for in part by reassignment from ICD codes that can include suicide deaths, such as undetermined intent injury codes (Y10-Y34 in ICD-10; E980-E988 in ICD-9) or exposure to unspecified factor (X59 in ICD-10; E887 in ICD-9), or as poorly defined or unknown causes of mortality (R99).^23^ First we determined a set of all possible “target causes” for the garbage codes to be redistributed onto based on knowledge of pathophysiology or certification practices to redistribute these garbage codes. Then we used a regression between suicide fractions and undetermined intent causes by age and sex in each location for each cause of injury to estimate the fraction allocated to each target code; the same regressions were implemented for homicide and unintentional injuries.We redistributed deaths coded to undetermined intent causes to suicide, homicide, and unintentional injury by using these fractions. Supplementary figure 1 shows the variation of direct assignment to suicide in women, aged 40 to 44, and suicide deaths that were reassigned from other causes in six countries for the year 2013.

### Estimation

We estimated suicide mortality by using the database described above, covariates (see supplementary table 3), and the cause of death ensemble model (CODEm) framework developed for the Global Burden of Disease Study 2016.^23^ Ensemble modelling is a term used to describe the combination of multiple independent model frameworks by using assessments of their relative predictive power. This method of modelling has been shown to estimate more accurate measures of uncertainty compared with other modelling techniques.^23^ Using ensemble models in the Global Burden of Disease Study 2016 allows models for countries with less reliable mortality data to borrow strength from adjacent geographies or time periods, by weighting the component models, to increase the accuracy of the final estimates and decrease uncertainty. Models for suicide were age limited such that deaths from suicide were restricted to a lower limit of age 10, owing to the very low numbers of suicide reported among those under the age of 10 in most populations and difficulty in determining intent in children.^26^ For this study, we developed individual CODEm models by sex, and separately for each country with or without extensive complete vital registration data. There are many model specifications. All models are systematically tested by using multiple iterations of cross-validation tests to evaluate the out-of-sample predictive validity of model variants that met predetermined requirements for direction and significance of regression coefficients. We incorporated models that performed best into a weighted, or “ensemble” model with the highest weights assigned to models with the best out-of-sample prediction error.^23^

We derived point estimates from the mean of 1000 draws from the posterior distribution of modelled suicide mortality by age, sex, and location; these 1000 draws for the final ensemble model are from each contributing model, with the number of draws being contributed proportional to the model’s weight in the final ensemble model. We derived 95% uncertainty intervals from the 2.5th and 97.5th centiles of these 1000 draws. We calculated age standardised mortality rates by using the time-invariant standard population developed for the Global Burden of Disease Study 2016. Years of life lost incorporate age at the time of death and can provide a more integrated view of the burden of suicide, particularly where mortality occurs at younger ages. We calculated years of life lost as the product of the mean age of death by age, sex, year, and location and the difference between the age at death and the standard life expectancy. In the Global Burden of Disease Study 2016, the standard life expectancy was 86.6 years, based on the lowest observed risk of death in each five year age group, from populations of greater than 5 million. Additional details of these calculations are available in the online appendix to the Global Burden of Disease Study 2016 Causes of Death publication.^23^

We compared rates and levels of suicide mortality and years of life lost among locations across age, sex, and country development status, for the years 1990 through 2016 and included 95% uncertainty intervals for each point estimate. The Socio-demographic index, developed for the Global Burden of Disease Study 2016 as a composite measure of development status,^23^ incorporates the geometric mean of total fertility rate, income per capita, and mean years of education among those aged over 15 to create an index for each location. Sex differences in suicide rates vary considerably, with a higher female to male ratio for countries lower on the development spectrum. We explored variation in sex ratios by Socio-demographic index level to identify locations where suicide mortality sex ratios differ from the expected patterns. Temporal change was evaluated from the difference in level or rate between time periods. Differences were considered statistically significant where the 95% uncertainty interval did not include zero (ie, where the quantity of interest increased (or decreased) in at least 95% of the draws).

### Patient and public involvement

The investigator did not conduct any interaction or intervention with individuals about whom data was obtained. Patients and the public were not involved in the design, analysis or interpretation of this research study.

## Results

### Global trends

There were 817 000 (95% uncertainty interval 762 000 to 884 000) deaths globally from suicide in 2016, comprising 1.49% (1.39% to 1.61%) of total deaths in that year and an all ages rate of 11.1 (10.3 to 12.0) deaths per 100 000. The total number of deaths from suicide increased globally between 1990 and 2016 by 6.7% (0.4% to 15.6%), but the age standardised mortality rate from suicide decreased by 32.7% (27.2% to 36.6%) from 16.6 deaths (15.2 to 17.6) per 100 000 in 1990 to 11.2 deaths (10.4 to 12.1) per 100 000 in 2016. In 2016, 34.6 (32.4 to 37.4) million years of life lost resulted from suicide. The global age standardised rate of years of life lost from suicide was estimated at 458.4 (438.5 to 506.1) per 100 000 in 2016 accounting for 2.18% (1.9% to 2.2%) of total years of life lost. This represented a decrease of 34.2% (28.4% to 38.1%) from 1990 when the age standardised years of life lost rate was 696.6 (641.4 to 744.2) per 100 000.

### Regional trends

Regionally, suicide was in the leading 10 causes of death in five of the 21 Global Burden of Disease defined regions. Suicide ranked 4th by age standardised mortality rate in eastern Europe, 6th in high income Asia Pacific, 7th in Australasia, and 10th in both central Europe and in high income North America (supplementary figure 2A). Ranked by age standardised years of life lost rate, suicide was the leading cause of death in high income Asia Pacific; 3rd leading cause in eastern Europe and Australasia; 4th in central Europe, western Europe, and high income North America; 6th in southern Latin America; and 8th in central Asia (supplementary fig 2B). Table 1 shows that the highest regional age standardised mortality rate in 2016 was estimated for eastern Europe (27.5 deaths per 100 000, 95% uncertainty interval 10.1 to 37.2), followed by high income Asia Pacific (18.7, 15.6 to 21.7), and southern sub-Saharan Africa (16.3, 14.3 to 19.3). Table 2 shows that a similar pattern was observed for regional age standardised years of life lost rates with the highest age standardised years of life lost rate estimated for eastern Europe (1200.3 years of life lost per 100 000, 95% uncertainty interval 869.2 to 1635.9), followed by high income Asia Pacific (742.0, 614.6 to 855.6) and southern sub-Saharan Africa (664.1, 579.6 to 809.8). The age standardised mortality rate for suicide decreased across most Global Burden of Disease regions between 1990 and 2016, with only non-significant increases observed for the regions of central Latin America (14.6%, 95% uncertainty interval −5.9% to 31.3%), high income Asia Pacific (10.1%, −23.5% to 30.0%), western sub-Saharan Africa (4.3%, −10.4% to 20.7%), and eastern Europe (1.4%, −24.2% to 34.3%). 

**Table 1:** Total number of deaths, age standardised mortality rate (ASMR) per 100 000 from suicide in 2016, and total percent change in ASMR from suicide 1990 to 2016, for all regions

Region	No of deaths (95% UI)		ASMR (95% UI)				Percent change (95% UI)	Female to male ASMR ratio
			Men	Women	Total			
East Asia	138 000 (129 000 to 154 000)		10.9 (9.8 to 12.8)	6.6 (6.1 to 7.1)	8.7 (8.2 to 9.7)		−63.0 (−65.7 to −55.8)	0.6
South East Asia	42 000 (39 000 to 50 000)		10.3 (9.1 to 12.5)	3.6 (3.3 to 4.0)	6.9 (6.3 to 8.0)		−34.0 (−41.6 to −17.3)	0.3
Oceania	1000 (1000 to 2000)		20.5 (14.5 to 28.5)	7.5 (4.8 to 11.6)	14.1 (10.1 to 17.4)		−13.0 (−27.7 to 8.2)	0.4
Central Asia	11 000 (9000 to 13 000)		21.1 (16.2 to 25.2)	4.8 (4.2 to 5.5)	12.5 (10.2 to 14.5)		−3.3 (−16.5 to 10.9)	0.2
Central Europe	19 000 (16 000 to 21 000)		22.6 (17.9 to 25.9)	4.2 (3.9 to 4.5)	13.0 (10.8 to 14.5)		−23.3 (−32.7 to −14.5)	0.2
Eastern Europe	68 000 (50 000 to 94 000)		50.0 (34.8 to 71.1)	8.3 (5.6 to 12.2)	27.5 (20.1 to 37.2)		1.4 (−24.1 to 34.4)	0.2
High income Asia Pacific	46 000 (38 000 to 53 000)		26.9 (20.1 to 32.6)	11.0 (9.3 to 12.9)	18.7 (15.6 to 21.7)		10.1 (−23.5 to 30.0)	0.4
Australasia	3000 (3000 to 4000)		16.4 (13.0 to 19.2)	5.0 (4.5 to 5.5)	10.6 (9.0 to 12.0)		−19.9 (−31.7 to −8.3)	0.3
Western Europe	53 000 (47 000 to 65 000)		15.2 (13.4 to 20.8)	4.3 (4.1 to 4.6)	9.6 (8.7 to 12.4)		−31.4 (−38.0 to −20.0)	0.3
Southern Latin America	8000 (7000 to 9000)		19.6 (16.0 to 24.0)	4.1 (3.6 to 4.7)	11.5 (9.7 to 13.6)		−15.0 (−27.3 to −1.1)	0.2
High income North America	51 000 (43 000 to 57 000)		20.0 (16.2 to 23)	5.8 (5.5 to 6.0)	12.7 (10.8 to 14.1)		−2.3 (−14.4 to 3.1)	0.3
Caribbean	4000 (4000 to 5000)		15.3 (12.9 to 17.6)	4.0 (3.3 to 4.7)	9.4 (8.3 to 10.6)		−24.5 (−32.9 to −15.5)	0.3
Andean Latin America	3000 (2000 to 3000)		7.4 (5.6 to 8.8)	2.9 (2.4 to 3.3)	5.1 (4.2 to 5.8)		−6.4 (−23.0 to 12.7)	0.4
Central Latin America	16 000 (13 000 to 19 000)		11.0 (8.5 to 13.4)	2.1 (1.9 to 2.3)	6.4 (5.2 to 7.6)		14.6 (−5.9 to 31.3)	0.2
Tropical Latin America	14 000 (12 000 to 18 000)		10.8 (8.8 to 14.0)	2.3 (2.0 to 2.6)	6.4 (5.3 to 7.9)		−11.6 (−22.4 to 4.4)	0.2
North Africa and Middle East	26 000 (23 000 to 31 000)		6.8 (5.9 to 8.4)	2.6 (2.4 to 3.0)	4.8 (4.2 to 5.6)		−2.8 (−15.7 to 14.3)	0.4
South Asia	252 000 (214 000 to 273 000)		18.2 (12.7 to 20.2)	12.6 (11.2 to 13.9)	15.4 (12.9 to 16.6)		−17.5 (−25.8 to −3.2)	0.7
Central sub-Saharan Africa	7000 (5000 to 9000)		16.6 (11.6 to 22.5)	6.0 (5.0 to 7.1)	10.9 (8.5 to 14.0)		−2.3 (−21.4 to 17.4)	0.4
Eastern sub-Saharan Africa	24 000 (21 000 to 28 000)		18.7 (15.8 to 22.1)	7.0 (5.9 to 8.2)	12.5 (11.1 to 14.1)		−15.3 (−26.5 to 2.3)	0.4
Southern sub-Saharan Africa	11 000 (10 000 to 13 000)		26.1 (21.9 to 31.6)	7.8 (6.7 to 8.9)	16.3 (14.3 to 19.3)		−10.8 (−25.0 to 19.1)	0.3
Western sub-Saharan Africa	19 000 (16 000 to 25 000)		12.6 (10.2 to 17.1)	6.7 (4.8 to 8.9)	9.6 (7.9 to 12.3)		4.3 (−10.4 to 20.7)	0.5

UI**=**uncertainty interval

**Table 2:** Total number of years of life lost (YLL) and age standardised rate (ASR) of YLL per 100 000 from suicide in 2016, and total percent change in ASR of YLL from suicide 1990 to 2016, for all regions

Region	No of YLL (95% UI)		ASR of YLL (95% UI)				Percent change (95% UI)	Female to male ASR of YLL ratio
			Men	Women	Total			
East Asia	4 665 000 (4 418 000 to 5 330 000)		353.7 (323.4 to 430.8)	223.8 (208.7 to 241.1)	289.6 (274.4 to 330.3)		−68.3 (−70.7 to −61.1)	0.6
South East Asia	1 864 000 (1 690 000 to 2 231 000)		420.1 (369.5 to 516.8)	134.9 (121.7 to 152.1)	276.1 (250.6 to 329.2)		−36.5 (−44.1 to −20.0)	0.3
Oceania	78 000 (54 000 to 99 000)		1013.2 (693.5 to 1409.7)	344.8 (206.5 to 547.7)	684.6 (472.7 to 858.2)		−12.2 (−28.0 to 10.6)	0.3
Central Asia	528 000 (418 000 to 617 000)		946.1 (704.9 to 1133.6)	224.0 (196.9 to 253.9)	575.1 (457.5 to 671.3)		1.8 (−14.2 to 17.2)	0.2
Central Europe	671 000 (550 000 to 746 000)		882.2 (687.8 to 994.6)	155.8 (145.1 to 168.4)	517.0 (420.4 to 574.9)		−23.0 (−34.5 to −14.3)	0.2
Eastern Europe	2 769 000 (2 000 000 to 3 767 000)		2118.2 (1455.2 to 3016.8)	346.4 (225.9 to 517.2)	1200.3 (869.2 to 1635.9)		5.8 (−22.5 to 42.8)	0.2
High income Asia Pacific	1 480 000 (1 228 000 to 1 708 000)		1034.7 (774.0 to 1248.3)	447.5 (377.6 to 533.3)	742.0 (614.6 to 855.6)		16.8 (−22.4 to 39.3)	0.4
Australasia	141 000 (119 000 to 159 000)		729.8 (593.9 to 856.9)	225.5 (202.9 to 251.5)	477.2 (409.0 to 539.6)		−21.4 (−33.5 to −10.1)	0.3
Western Europe	1 785 000 (1 617 000 to 2 346 000)		602.6 (532.4 to 859.4)	167.5 (158.5 to 178.1)	384.1 (347.2 to 514.0)		−32.2 (−39.8 to −21.5)	0.3
Southern Latin America	340 000 (277 000 to 392 000)		838.8 (645.7 to 998.1)	188.1 (162.9 to 217.6)	510.2 (414.4 to 589.8)		−7.1 (−21.1 to 5.5)	0.2
High income North America	2 062 000 (1 745 000 to 2 270 000)		856.4 (696.9 to 973.2)	257.6 (245.3 to 268.7)	556.4 (473.7 to 613.3)		−3.1 (−16.2 to 2.5)	0.3
Caribbean	174 000 (153 000 to 199 000)		598.1 (515.9 to 707.4)	160.9 (128.4 to 199.7)	375.9 (329.7 to 429.7)		−26.8 (−35.0 to −17.6)	0.3
Andean Latin America	140 000 (115 000 to 159 000)		324.8 (243.3 to 385.6)	136.6 (116.6 to 157.4)	230.0 (190.4 to 262.4)		−5.9 (−23.5 to 13.7)	0.4
Central Latin America	813 000 (657 000 to 988 000)		503.8 (382.8 to 630.8)	106.2 (98.4 to 117.2)	302.4 (245.3 to 365.1)		18.8 (−4.3 to 39.0)	0.2
Tropical Latin America	649 000 (543 000 to 803 000)		464.7 (373.9 to 603.1)	103.0 (90.4 to 117.1)	280.9 (235.1 to 348.2)		−6.8 (−18.7 to 12.0)	0.2
North Africa and Middle East	1 281 000 (1 141 000 to 1 532 000)		308.6 (266.7 to 382.3)	117.8 (104.4 to 132.3)	216.0 (192.0 to 257.1)		−2.9 (−16.4 to 15.9)	0.4
South Asia	12 636 000 (10 923 000 to 13 692 000)		782.9 (562.1 to 868.9)	626.6 (556.1 to 690.5)	704.8 (605.1 to 764.8)		−23.0 (−30.9 to −9.7)	0.8
Central sub-Saharan Africa	275 000 (208 000 to 374 000)		532.4 (370.4 to 747.5)	180.2 (150.9 to 220.7)	351.3 (269.0 to 462.0)		−4.1 (−24.1 to 16.1)	0.3
Eastern sub-Saharan Africa	969 000 (848 000 to 1 111 000)		555.7 (464.5 to 665.8)	203.8 (172.4 to 236.9)	373.5 (330.5 to 425.2)		−16.9 (−29.1 to 2.8)	0.4
Southern sub-Saharan Africa	510000 (442000 to 628000)		1056.5 (896.2 to 1341.8)	296.1 (250.8 to 344.3)	664.1 (579.6 to 809.8)		−16.2 (−30.1 to 15.1)	0.3
Western sub-Saharan Africa	783 000 (644 000 to 1 020 000)		397.1 (316.9 to 543.2)	182.2 (133.9 to 240.8)	288.9 (238.1 to 375.7)		4.4 (−10.5 to 22.6)	0.5

UI**=**uncertainty interval

**Fig 1:**
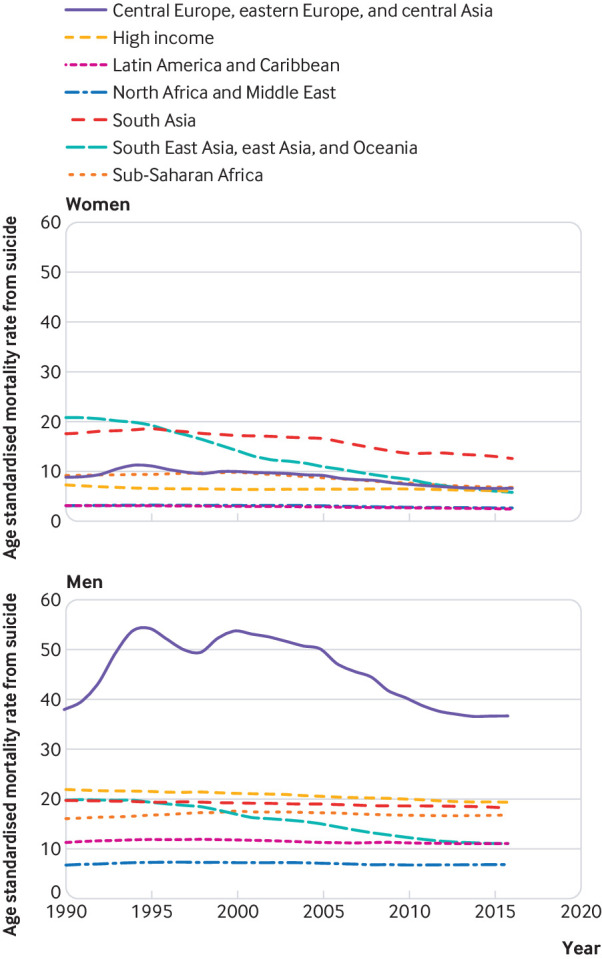
Age standardised mortality rate from suicide, by Global Burden of Disease super region, for women and men, 1990 to 2016

Figure 1 shows that there were periods of increases and declines in the age standardised mortality rate from suicide for men in eastern Europe in particular. The age standardised mortality rate from suicide in for men in eastern Europe was similar at the beginning and end of the study period (27.1 deaths per 100 000, 95% uncertainty interval 23.8 to 34.1 in 1990; 27.5, 20.1 to 37.2 in 2016) and rose as high as 42.8 deaths per 100 000 (95% uncertainty interval 33.7 to 50.2) during this period.

### National trends

**Fig 2:**
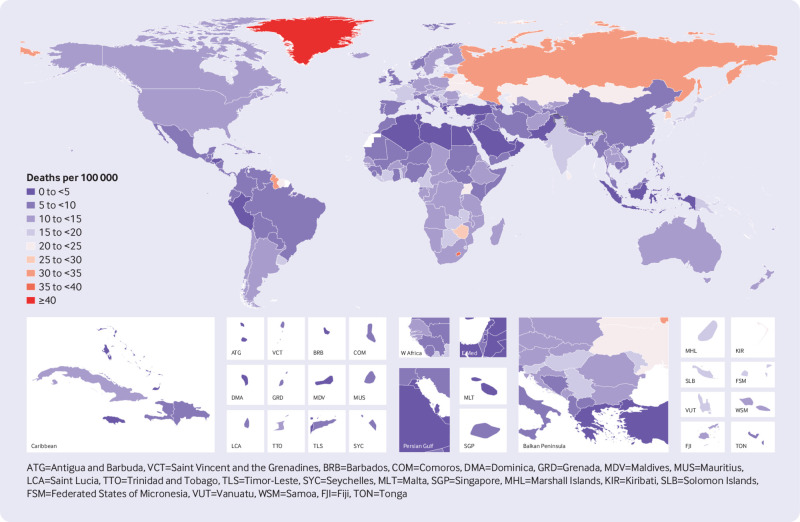
Age standardised mortality rate from suicide for men and women combined, 2016

**Fig 3:**
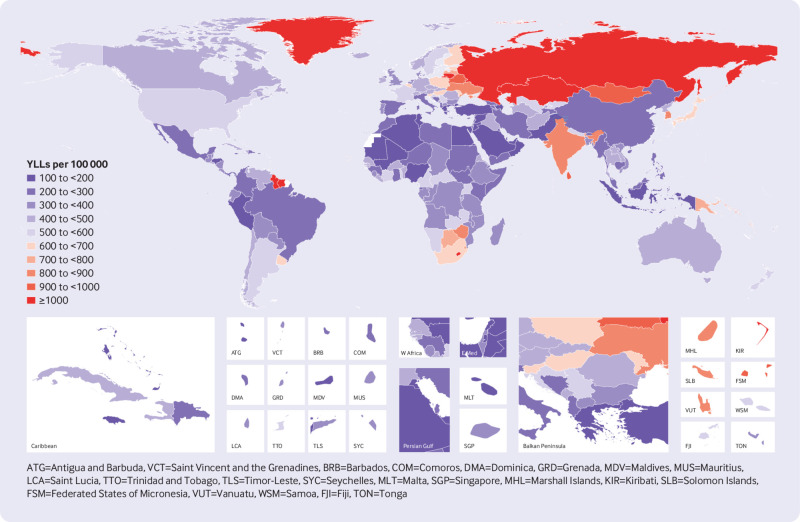
Age standardised rate of years of life lost (YLL) from suicide for men and women combined, 2016

Considerable variability emerged beneath these regional patterns. High rates of suicide mortality in a few countries influenced regional averages in Global Burden of Disease regions, particularly South Korea in high income Asia Pacific, Indonesia in South East Asia, and Lesotho and Zimbabwe in Southern sub-Saharan Africa (supplementary table 1). Figure 2 shows that in 2016, for countries with populations greater than 1 million, age standardised mortality rates from suicide were highest in Lesotho (39.0 deaths per 100 000, 95% uncertainty interval 25.5 to 55.7), Lithuania (31.0, 25.6 to 36.2), Russia (30.6, 20.6 to 43.6), and Zimbabwe (27.8, 21.1 to 37.3). Figure 3 shows that in 2016, for countries with populations greater than 1 million, rates of age standardised years of life lost were highest in Lesotho (1413.2 years per 100 000, 95% uncertainty interval 944.9 to 2065.9), Russia (1349.5, 889.7 to 1922.4), Lithuania (1317.8, 1065.1 to 1547.5), Kazakhstan (1119.9, 858.9 to 1462.7), and Mongolia (998.1, 744.3 to 1230.5). 

Figure 2 shows that age standardised mortality rates for suicide were lowest in Lebanon (2.4 deaths per 100 000, 95% uncertainty intervals 1.6 to 3.5), Syria (2.5, 2.0 to 3.0), Palestine (2.7, 2.1 to 3.6), Kuwait (2.7, 1.7 to 3.8), and Jamaica (2.9, 2.2 to 3.7). Figure 3 shows that rates of age standardised years of life lost rates were lowest in the same countries.

**Fig 4:**
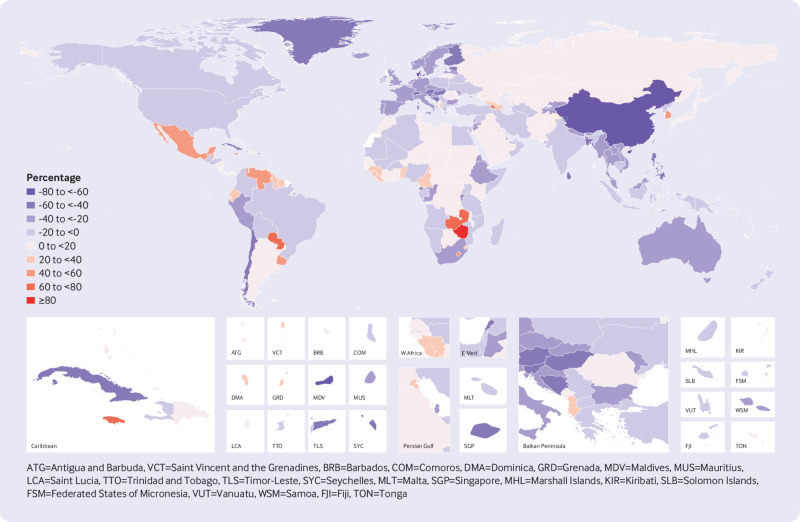
Percentage change in age standardised mortality rate from suicide for men and women combined, 1990 to 2016

Between 1990 and 2016, noticeable decreases in age standardised mortality rates from suicide were estimated for 63 of the 195 countries and territories in the Global Burden of Disease Study 2016 (supplementary table 1). Figure 4 shows that the largest statistically significant decreases occurred in China (64.1%, 95% uncertainty interval 57.1% to 66.7%), Denmark (60.0%, 37.6% to 68.3%), the Philippines (58.1%, 10.4% to 69.8%), Singapore (50.6%, 31.0% to 62.9%), and Switzerland (50.3%, 27.9% to 63.5%), as well as in the smaller countries of the Maldives (59.1%, 36.3% to 72.0%) and Seychelles (56.1%, 26.6% to 65.9%). Deaths from suicide in China and India – as the most populous countries – together constituted 44.2% of global suicide deaths in 2016. However, the large decrease in the age standardised mortality rate from suicide in China over the past 27 years was not matched in India where the age standardised mortality rate decreased by 15.2% (95% uncertainty interval 0.3% to 23.7%). Globally, the age standardised mortality rate from suicide increased in some locations, with the largest statistically significant increases estimated for Zimbabwe (96.2%, 95% uncertainty interval 30.7% to 268.4%), Jamaica (70.9%, 21.0% to 128.2%), Paraguay (70.4%, 23.2. to 117.1), Zambia (61.6%, 5.23% to 128.7%), and Belize (52.2%, 8.8% to 100.7%).

### Sex and age trends

Globally, the age standardised mortality rate was higher for men (15.6 deaths per 100 000, 95% uncertainty interval 13.7 to 17.2) than for women (7.0, 6.5 to 7.4), however, the rate of decrease from 1990 to 2016 was lower for men (23.8%, 95% uncertainty interval 15.6% to 32.7%) than for women (49.0%, 42.6% to 54.6%). This was reflected in the female to male ratio which was 0.73 in 1990 and 0.46 in 2016. In 2016, for countries with a population greater than 1 million, the age standardised mortality rates from suicide were highest for men in Lithuania (56.6 deaths per 100 000, 95% uncertainty interval 44.4 to 67.7), Russia (55.3, 34.9 to 85.0), and Kazakhstan (44.2, 32.5 to 59.6). The age standardised mortality rate for suicide were highest for women in Lesotho (35.4, 16.8 to 54.7), Uganda (18.7, 12.7 to 25.5), Liberia (17.0, 12.5 to 21.5), and South Korea (15.5, 10.0 to 22.7) (supplementary table 1). 

The age standardised mortality rates from suicide in 2016 were lowest for men in Lebanon (3.4 deaths per 100 000, 95% uncertainty interval 2.0 to 5.6), Kuwait (3.8, 2.1 to 5.7), Pakistan (3.9, 2.9 to 5.2), Syria (4.0, 3.1 to 5.1), and Palestine (4.1, 3.0 to 5.7). The age standardised mortality rate from suicide were lowest for women in Syria (1.0, 0.8 to 1.2), Oman (1.1, 0.9 to 1.3), Jamaica (1.1, 0.8 to 1.5), and Greece (1.2, 1.0 to 1.4).

Although age standardised mortality rates were lower for women than for men across all regions, strong regional variation in relative rates by sex existed in 2016. Table 1 shows that the lowest ratios of suicide mortality rates for women compared with those for men occurred in the regions of eastern (0.17) and central (0.18) Europe, and across central (0.19), southern (0.21), and tropical (0.21) Latin America. This ratio was closest to parity in east (0.60) and south (0.69) Asia. At the national level, rates of suicide mortality for women were lower than those for men in all countries except for Liberia (1.08) (supplementary table 1). Relative ratios of female to male age standardised mortality rates were also comparatively high for many countries across Africa (Morocco 0.95, Nigeria 0.88, and Lesotho 0.88), as well as for many countries in south Asia (Pakistan 0.96, Bangladesh 0.73, and India 0.69).

**Fig 5:**
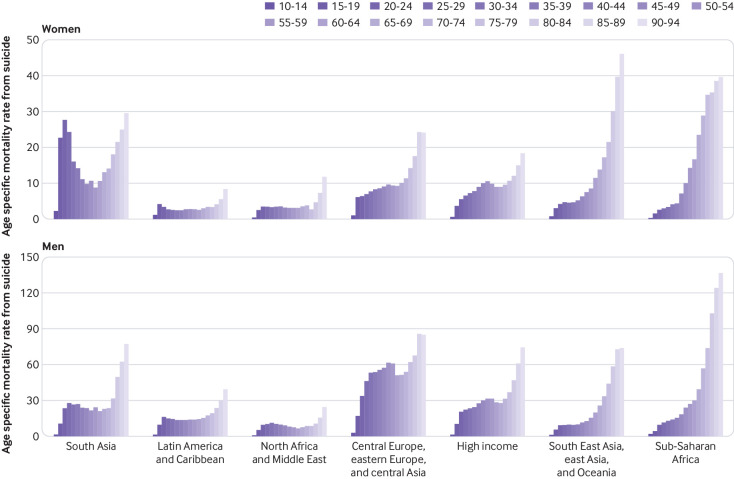
Age specific mortality rate from suicide by Global Burden of Disease super region and five year age groups for women and men, 2016

Figure 5 shows that, for men and women and across all regions, rates of suicide mortality were highest for the oldest age groups. However, suicide did not rank in the leading 10 causes of death for those aged 70 and over (supplementary fig 3A). At the same time, regional variation in the overall age distribution of mortality rates from suicide was notable – with comparatively high rates for younger adults in several regions. In the 10 to 24 age group, suicide was ranked in the leading five causes of mortality in all Global Burden of Disease regions except for central, eastern, western, and southern sub-Saharan Africa (supplementary fig 3B). Figure 5 shows that a bimodality in the distribution of age specific mortality rates was particularly evident for women in south Asia and for men in central Europe, eastern Europe, and central Asia and in north Africa and Middle East. Across the time series, global mortality rates from suicide were greater, although not significantly so, for women than for men only among those in the 15 to 19 age group (supplementary table 4), although the gap in this age group has also decreased over time (supplementary fig 4). In 2016 mortality rates from suicide for men exceeded those for women in all other age groups by as much as 2.98 times.

For both sexes combined, higher levels of suicide deaths occurred before age 30 in south Asia (46.0% of all suicide deaths), Latin America and Caribbean (38.7%), and North Africa and the Middle East (38.6%) (supplementary fig 5). These higher proportions of suicide deaths among those under 30 reflects the age structure of these populations as age specific mortality rates in the under 30 populations are relatively lower compared with rates at older ages, except for women in south Asia. Deaths from suicide for those younger than 30 comprised less than 30% of total suicide deaths in other Global Burden of Disease super regions. One fifth of suicide deaths occurred among those aged 60 or over in the Global Burden of Disease super regions of high income (20.8%), and South East Asia, east Asia, and Oceania (24.8%).

###  Socio-demographic index and differences by sex

**Fig 6:**
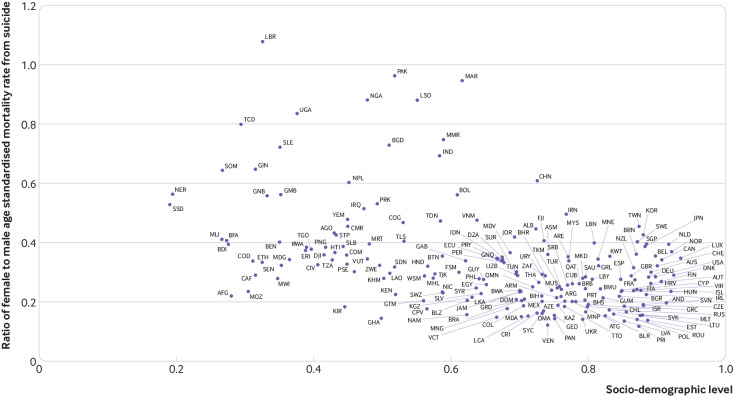
Ratio of female to male age standardised mortality rate for suicide by Socio-demographic index level by location, 2016. Location codes are described in full in supplementary table 5

Globally, ratios of suicide deaths were skewed strongly towards lower rates for women, but there was a large variation between locations related to Socio-demographic index level. Figure 6 shows that the ratio of age standardised mortality rates for women compared with men were highest in countries with a low Socio-demographic index, however, not all countries followed this trend (eg, China has a Socio-demographic index 0.73 and a female to male ratio of 0.61). For the 23 countries with a female to male ratio of 0.50 or greater, there was no clear association between Socio-demographic index and this ratio. A small number of countries were estimated to have both a relatively low Socio-demographic index and a low female to male ratio of age standardised mortality rate from suicide including Afghanistan, Mozambique, Malawi, and Central African Republic.

## Discussion

Suicide continues to be an important cause of preventable mortality worldwide, resulting in an estimated 817 000 deaths in 2016. When ranking leading causes by age standardised mortality rate, this study found that suicide deaths were in the leading 10 causes of death across eastern Europe, central Europe, high income Asia Pacific, Australasia, and high income North America. When ranked by age standardised years of life lost rates, suicide was the leading cause of death in high income Asia Pacific and was among the top 10 leading causes in eastern Europe, central Europe, western Europe, central Asia, Australasia, southern Latin America, and high income North America. Suicide continues to be an important cause of mortality in most countries worldwide, but it is promising that both the global age standardised mortality rate and years of life lost rate from suicide have decreased by a third between 1990 and 2016. Globally, the decline in the age standardised mortality rate from suicide (32.7%) we noted is similar to that estimated by the Global Burden of Disease Study 2016 for all cause mortality (30.6%).^23^ Whether the decline in suicide mortality is due to suicide prevention activities, or whether it reflects general improvements to population health, warrants further research.

The decline in the age standardised mortality rate has not been universal. There is considerable variation in suicide mortality and rates of change between the sexes, across age groups, and between regions. Consistent with previous research, men had higher rates of suicide at all time points, for all age groups except for among those aged 15 to 19.^27^ ^28^ ^29^ In this age group women had consistently higher rates of suicide mortality; however, this gap narrowed between 1990 and 2016 to almost parity in 2016. A greater decline in the global suicide mortality rate was estimated for women (49.0%) than men (23.8%), and if this trend continues, differences in suicide mortality by sex will continue to widen. The association between lower levels of Socio-demographic index and higher female to male ratios warrants further research to understand if this pattern reflects a higher level of behaviour related to suicide among women in these countries, or a relatively higher level of lethality of the means used by women in countries with lower development contexts.^18^ ^28^ ^30^ Regional variation is also apparent. For example, post-communist privatisation and the Russian economic crisis of 1998 were followed by increases in suicide mortality in eastern Europe, in contrast to a general pattern of decreasing mortality rates overall.^31^ ^32^ Much of the global estimated decline is owing to the large decrease in suicide mortality in China during the study period, and the lower but still important decrease in India. The changes observed in China have been attributed to economic growth, urbanisation, improved standards of living, and better access to medical care in rural areas.^33^ ^34^ Page and colleagues report that the share of pesticide poisoning suicides, the most common means of suicide in China, decreased from 55% to 49% between 2006 and 2013.^35^ Further research to understand if changes to availability or lethality of pesticides has contributed to this decline could suggest a scalable public health intervention for other similar contexts.

Taken as a whole, these patterns reflect a complex interplay of factors, specific to regions and nations, including sociodemographic, sociocultural, and religious factors^6^ ^36^; levels of economic development, unemployment and economic events 7 9; distribution of risk factors, such as exposure to violence or use of alcohol and drugs 8 10; choices of and access to means of suicide 12 37; and patterns of mental illness and as well as culturally specific relations with suicide.^15^ ^21^ Moreover, although the decrease in suicide mortality has been substantial during the period 1990 to 2016, if current trends continue, only 3% of 118 countries will attain the Sustainable Development Goals target to reduce suicide mortality by one third between 2015 and 2030.^38^

### Strengths and weaknesses of this study

In estimating suicide mortality rates by using methods that are comparable over time and between countries for the period 1990 to 2016, this study provides comprehensive estimates of suicide mortality and years of life lost by age, sex, region, and country. In addition, this study improves on country-level estimates through data standardisation and redistribution of insufficiently detailed or implausible cause of death codes, which is particularly relevant for suicide deaths. This study is limited by the gaps in data, especially among low and middle income countries, and variations in data quality in the Global Burden of Disease Study. The Global Burden of Disease Study 2016 employs data standardisation and evaluations of data quality in a sophisticated modelling framework that borrows strength across space and time to address some of these issues.^23^ Because of the social, cultural, religious, and sometimes legal ramifications of a death by suicide, there are additional concerns with respect to under-reporting of suicide. A recent systematic review concluded that in general, suicide deaths are under-reported, although more research on the reliability of suicide data, especially in low and middle income countries, is needed.^25^ Moreover, under-reporting and misclassification of suicide could be affected by sociocultural factors,^39^ resulting in differential misclassification. Owing to this, lower rates in countries with religious and cultural sanctions against suicide should be interpreted with caution. Although the Global Burden of Disease Study 2016 method to reclassify garbage codes adjusts for some of this misclassification,^23^ this could still be a conservative estimate of the suicide mortality rate if suicide deaths are wrongly assigned to other plausible causes, such as unintentional injuries, when deaths are recorded.^22^ ^25^ This paper has not reported the burden of suicide attributable to risk factors such as mental disorders, drug and alcohol use, or violence 10 14; further research quantifying the contribution of these and other risk factors to suicide mortality would be useful in informing interventions to prevent suicide.

### Conclusion and policy implications

Although there has been progress on reducing suicide mortality in recent decades, suicide remains an important preventable contributor to the global burden of disease across all regions. National, regional, sex, and age related variations yield insight which can inform suicide prevention initiatives, adding to existing effective suicide prevention interventions.^17^ Sociocultural and economic factors must also be considered, as temporal and global patterns underscore their likely contribution to suicide mortality.^7^ ^9^ A continued focus on strengthening data on suicide deaths is needed, including accurately capturing suicide deaths in low and middle income countries,^25^ as well as data on means of suicide.^11^ ^40^ Similarly, more evidence on effective suicide prevention interventions is needed, particularly in low and middle income countries. The evaluation of national and regional suicide prevention strategies, such as Preventing Suicide in England,^41^ the National Suicide Prevention Plan 2015-2020 for Guyana,^42^ and the Fiji National Mental Health and Suicide Prevention Policy will provide evidence to support the public health community to strengthen effective efforts and to redirect activities that are not impactful.^5^ ^17^ ^43^ However, care must be taken to not apply strategies that work to dissimilar contexts. Research must continue to build the evidence base for effective interventions that are sensitive to regional and national contexts to address this continuing public health concern.

**What is already known on this topic**

Suicide is a global public health concern
The World Health Organization reports approximately 800 000 global suicide deaths annually
Men, younger adults, and older adults are reported to have higher rates of suicide than women and middle aged adults

**What this study adds**

The global age standardised mortality rate from suicide decreased by almost a third between 1990 and 2016
Men had higher mortality rates from suicide than women, at all ages except for the 15 to 19 age group
Men experienced a lower decrease in age standardised mortality rate from suicide from 1990 to 2016 (23.8%) compared with women (49.0%)

## Data Availability

Data sources and code used in the Global Burden of Disease Study 2016 can be accessed at the following address: http://ghdx.healthdata.org/gbd-2016.

## Web Extra

Extra material supplied by the author

Supplementary materials: Supplementary figures 1 to 5 and tables 1 to 5

Supplementary materials: Full author list and affiliations

## Notes

Contributors: HMO, LBM, MA, and MN prepared the first draft. HMO, LBM, MA, and MN analysed the data and edited the first draft and final versions of the manuscript. HMO, LBM, CJLM, and MN finalised all drafts and approved the final version of the manuscript. All other authors provided data, developed models, reviewed results, provided guidance on methodology, or reviewed the manuscript, and approved the final version of the manuscript. All authors had full access to the data in the study and can take responsibility for the integrity of the data and the accuracy of the data analysis. MN is the guarantor. The corresponding author attests that all listed authors meet authorship criteria and that no others meeting the criteria have been omitted.

The lead author (MN) affirms that this manuscript is an honest, accurate, and transparent account of the study being reported; no important aspects of the manuscript have been omitted; and discrepancies from the study as planned have been explained.
