# Supplementary material for: Global, regional, and national burden of suicide mortality 1990 to 2016: systematic analysis for the Global Burden of Disease Study 2016
Source: BMJ. 2019 Feb 6;364:l94. doi: 10.1136/bmj.l94 (PMC6598639; doi:10.1136/bmj.l94)
Supplement: Supplementary file 1 — Supplementary materials: Supplementary figures 1 to 5 and tables 1 to 5 [file nagm045998.ww1.pdf]

Supplementary Figure 1. Fraction of deaths classified as suicide in 40-44 year old females for the year 2013, from source of death classification

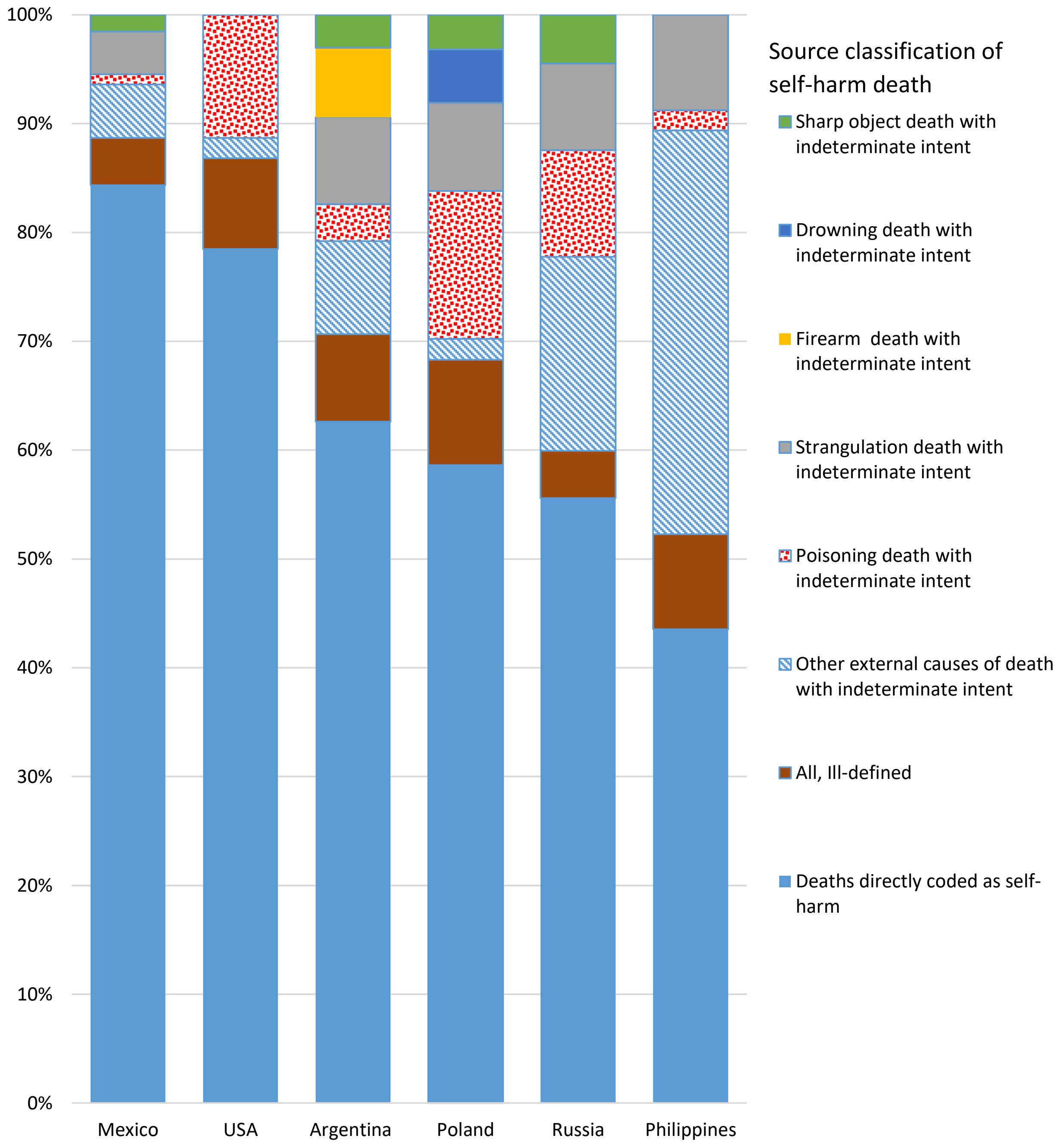

Supplementary Figure 2A: Both sexes, Age-standardized, 2016, Deaths per 100,000

2016

	East Asia	Andean Latin America	Australasia	Caribbean	Central Asia	Central Europe	Central Latin America	Central Sub-Saharan Africa	Eastern Europe	Eastern Sub-Saharan Africa	High-income Asia Pacific	High-income North America	North Africa and Middle East	Oceania	South Asia	Southeast Asia	Southern Latin America	Southern Sub-Saharan Africa	Tropical Latin America	Western Europe	Western Sub-Saharan Africa
Ischemic heart disease	1	1	1	1	1	1	1	2	1	1	2	1	1	1	1	1	1	2	1	1	1
Stroke	2	3	3	2	2	2	5	3	2	4	3	4	2	2	4	2	2	5	2	3	3
COPD	3	8	5	9	5	7	6	10	11	10	12	5	7	4	2	6	4	7	5	5	10
Alzheimer disease	4	5	2	5	6	4	4	8	3	8	1	2	3	9	9	3	5	10	3	2	9
Lung cancer	5	11	4	8	8	3	13	31	6	36	4	3	11	14	18	9	6	14	10	4	34
Liver cancer	6	16	24	27	15	22	19	23	34	28	9	27	19	30	45	14	33	27	27	20	13
Stomach cancer	7	7	20	20	10	15	14	32	12	33	7	29	17	19	21	20	11	32	14	15	26
Road injuries	8	9	16	12	12	16	9	14	9	15	13	11	6	10	11	10	10	9	8	21	16
Hypertensive heart disease	9	15	35	11	3	8	10	12	15	12	21	16	10	11	13	11	13	11	11	16	23
Lower respiratory infections	10	2	9	4	4	9	8	4	8	3	5	6	8	5	5	4	3	3	4	7	2
Esophageal cancer	11	60	27	46	23	39	59	40	47	30	20	32	52	76	39	53	32	19	29	28	47
Chronic kidney disease	12	4	10	7	11	17	2	22	33	20	10	8	5	7	8	8	7	13	9	13	19
Colorectal cancer	13	14	6	13	17	6	16	33	7	23	8	7	18	27	28	16	9	23	13	6	33
Diabetes	14	6	8	3	7	11	3	11	31	9	14	9	4	3	6	5	8	4	6	10	8
Falls	15	24	15	23	37	19	24	19	20	16	22	18	25	46	10	15	37	44	18	17	17
Self-harm	16	27	7	18	13	10	21	26	4	21	6	10	28	15	14	24	15	15	24	11	25
Congenital defects	17	17	37	19	25	33	18	36	32	68	36	34	14	21	26	26	23	36	20	36	22
Drowning	18	42	68	37	29	50	40	37	23	42	33	63	46	20	37	33	55	45	42	74	39
Rheumatic heart disease	19	56	54	43	30	55	77	43	57	57	46	45	49	12	16	42	26	43	71	46	42
Cirrhosis hepatitis B	20	28	44	67	9	38	76	24	30	27	28	73	21	16	23	18	42	47	45	55	20
Other cardiovascular	21	25	17	17	26	12	23	13	25	7	19	15	12	13	71	21	12	25	15	9	12
Other neoplasms	22	30	18	24	27	21	22	39	24	25	16	21	29	39	22	27	18	33	23	18	37
Neonatal preterm birth	23	21	52	21	22	43	25	27	58	29	61	39	15	22	15	22	29	24	28	50	21
Pancreatic cancer	24	29	13	30	32	14	31	50	19	52	11	13	30	52	54	38	17	34	26	12	38
Breast cancer	25	23	12	16	18	13	20	30	14	34	17	12	20	17	24	19	14	21	19	8	24

Supplementary Figure 2B: Both sexes, Age-standardized, 2016, YLLs per 100,000

2016

	East Asia	Andean Latin America	Australasia	Caribbean	Central Asia	Central Europe	Central Latin America	Central Sub-Saharan Africa	Eastern Europe	Eastern Sub-Saharan Africa	High-income Asia Pacific	High-income North America	North Africa and Middle East	Oceania	South Asia	Southeast Asia	Southern Latin America	Southern Sub-Saharan Africa	Tropical Latin America	Western Europe	Western Sub-Saharan Africa
Stroke	1	4	4	2	2	2	8	7	2	6	3	5	2	2	3	2	2	8	3	3	6
Ischemic heart disease	2	2	1	1	1	1	1	6	1	5	2	1	1	1	1	1	1	4	1	1	5
Road injuries	3	3	6	5	6	6	5	9	5	13	10	3	3	8	9	5	4	6	4	8	14
COPD	4	15	7	17	11	10	10	16	17	16	17	6	11	5	5	9	9	12	8	7	17
Lung cancer	5	19	2	13	13	3	17	40	9	54	5	2	13	22	26	12	7	18	12	2	45
Liver cancer	6	24	25	38	23	27	29	30	42	32	7	28	27	40	57	17	34	35	38	22	16
Congenital defects	7	6	10	7	9	9	7	14	11	35	12	12	5	11	14	10	5	17	6	10	11
Stomach cancer	8	9	24	31	15	18	16	43	14	44	6	35	26	32	32	30	15	40	17	17	39
Neonatal preterm birth	9	7	17	9	7	13	9	10	31	11	28	14	6	10	7	7	8	10	10	18	9
Alzheimer disease	10	13	8	18	21	11	11	21	21	26	4	8	10	23	23	11	16	25	9	5	26
Lower respiratory infections	11	1	19	3	3	7	6	3	7	3	9	11	7	3	4	3	3	2	5	11	3
Drowning	12	21	39	24	17	33	27	22	16	34	27	42	32	13	22	19	31	34	25	55	31
Self-harm	13	16	3	16	8	4	13	24	3	24	1	4	21	12	11	18	6	13	14	4	25
Neonatal encephalopathy	14	11	29	10	5	47	15	11	35	9	53	37	20	18	8	13	36	11	15	37	7
Esophageal cancer	15	76	30	56	36	41	68	51	48	42	21	33	56	85	50	67	37	27	36	29	65
Hypertensive heart disease	16	33	55	19	4	15	24	19	25	20	36	23	17	16	18	16	21	15	21	33	38
Chronic kidney disease	17	5	20	12	14	23	3	34	38	28	13	15	9	7	13	8	10	14	11	23	21
Colon and rectum cancer	18	23	5	23	28	5	26	48	13	38	8	9	25	41	42	23	12	33	19	6	43
Falls	19	32	28	37	34	22	30	35	20	25	20	29	24	51	15	22	41	55	28	24	23
Diabetes	20	8	12	6	10	14	4	15	39	14	16	13	8	4	12	4	11	5	7	15	15
Leukemia	21	20	14	27	30	25	21	63	33	69	18	24	23	42	51	37	24	42	33	16	63
Mechanical forces	22	38	50	39	49	52	37	46	37	51	44	38	37	37	58	65	40	38	49	63	40
Other neoplasms	23	31	18	26	29	21	22	45	28	23	15	22	33	43	28	31	18	29	26	14	44
Cirrhosis hepatitis B	24	36	40	67	12	34	76	27	27	31	23	70	31	20	29	24	44	46	45	49	19
Brain cancer	25	41	15	49	35	20	41	76	34	71	31	25	35	80	65	48	39	58	31	19	66

Supplementary Figure 3A: Both sexes, 70+ years, Deaths per 100,000

2016

	East Asia	Andean Latin America	Australasia	Caribbean	Central Asia	Central Europe	Central Latin America	Central Sub-Saharan Africa	Eastern Europe	Eastern Sub-Saharan Africa	High-income Asia Pacific	High-income North America	North Africa and Middle East	Oceania	South Asia	Southeast Asia	Southern Latin America	Southern Sub-Saharan Africa	Tropical Latin America	Western Europe	Western Sub-Saharan Africa
Ischemic heart disease	1	1	1	1	1	1	1	1	1	1	2	1	1	1	1	1	1	1	1	1	1
Cerebrovascular disease	2	5	3	2	2	2	6	2	2	2	3	3	2	2	4	2	3	2	3	3	3
Chronic obstructive pulmonary disease	3	6	4	7	5	5	5	7	4	8	9	4	5	3	2	4	5	5	4	4	8
Alzheimer disease and other dementias	4	3	2	3	4	3	2	6	3	7	1	2	3	9	7	3	4	6	2	2	6
Tracheal, bronchus, and lung cancer	5	12	5	10	9	8	11	23	6	29	5	5	11	12	14	9	10	13	10	5	23
Hypertensive heart disease	6	11	23	9	3	6	8	8	7	10	14	15	8	10	12	10	8	7	8	12	15
Stomach cancer	7	8	18	14	8	14	10	21	8	25	6	26	13	14	17	20	13	24	14	17	19
Liver cancer	8	14	30	17	13	22	15	19	24	26	10	27	20	24	37	16	28	22	23	23	13
Lower respiratory infections	9	2	7	5	10	9	7	4	13	4	4	6	7	6	5	5	2	4	5	6	2
Chronic kidney disease	10	4	8	8	7	13	4	18	21	14	8	7	6	7	10	8	6	10	7	9	14
Esophageal cancer	11	44	27	36	17	42	47	27	35	22	26	31	44	53	32	44	25	16	26	30	39
Diabetes mellitus	12	7	10	4	6	10	3	9	18	9	13	9	4	4	6	6	7	3	6	10	7
Colon and rectum cancer	13	13	6	11	12	7	13	22	5	19	7	8	14	19	18	14	9	18	13	7	24
Falls	14	21	11	13	35	16	19	17	26	11	23	12	23	35	9	13	26	32	16	14	12
Other cardiovascular and circulatory diseases	15	20	12	12	18	11	17	11	17	6	16	10	9	11	42	17	11	17	12	8	11
Rheumatic heart disease	16	40	44	38	27	44	67	35	52	50	37	35	46	13	13	41	20	41	65	34	33
Atrial fibrillation and flutter	17	19	13	16	15	19	20	34	11	32	17	17	22	34	20	23	18	26	20	13	35
Road injuries	18	17	48	27	32	38	24	32	34	16	31	39	15	27	16	19	33	20	24	48	17
Parkinson disease	19	26	16	22	16	18	27	51	12	47	21	14	21	40	29	26	17	37	28	19	41
Self-harm	20	59	49	33	34	29	58	28	20	24	22	43	57	45	33	46	43	30	58	42	26

Supplementary Figure 3B: Both sexes, 10 to 24, Deaths per 100,000

2016

	East Asia	Andean Latin America	Australasia	Caribbean	Central Asia	Central Europe	Central Latin America	Central Sub-Saharan Africa	Eastern Europe	Eastern Sub-Saharan Africa	High-income Asia Pacific	High-income North America	North Africa and Middle East	Oceania	South Asia	Southeast Asia	Southern Latin America	Southern Sub-Saharan Africa	Tropical Latin America	Western Europe	Western Sub-Saharan Africa
Road injuries	1	1	2	1	2	1	2	1	2	4	2	1	2	1	2	1	1	3	2	1	6
Drowning	2	6	10	5	4	3	4	8	4	9	5	5	5	3	6	3	4	10	4	10	10
Self-harm	3	3	1	4	1	2	3	12	1	10	1	2	3	2	1	4	2	6	3	2	12
Leukemia	4	5	5	11	10	5	5	29	13	36	3	8	9	19	27	16	5	18	6	4	34
Falls	5	19	14	33	12	11	15	27	6	18	7	18	15	38	16	15	16	45	15	13	24
Congenital birth defects	6	14	7	8	15	9	9	24	10	47	6	6	7	17	23	10	9	13	10	6	11
Ischemic heart disease	7	10	27	21	9	16	11	39	15	24	13	15	8	15	8	11	14	42	11	22	32
Cerebrovascular disease	8	8	20	9	19	13	12	21	21	17	14	17	10	6	32	12	10	12	9	17	18
Exposure to mechanical forces	9	11	15	10	22	15	8	17	12	25	18	11	11	14	31	28	7	11	13	15	20
Interpersonal violence	10	2	4	2	5	6	1	9	3	7	9	3	4	8	7	6	3	2	1	8	16
Other unintentional injuries	11	27	30	20	17	18	20	38	16	34	35	36	18	37	25	20	12	47	14	32	37
Brain and nervous system cancer	12	17	9	31	16	8	18	53	18	49	8	12	19	62	37	27	15	35	12	7	47
Other neoplasms	13	15	6	14	13	7	10	22	14	11	4	7	14	23	19	17	8	14	7	5	30
Drug use disorders	14	23	3	45	24	12	25	54	5	87	25	4	25	61	45	39	25	25	20	3	50
Epilepsy	15	18	11	17	6	14	14	16	26	12	12	25	17	30	12	22	19	9	21	11	19
Chronic kidney disease	16	13	33	13	7	22	6	30	30	23	24	30	13	16	15	8	17	19	17	33	17
Lower respiratory infections	17	4	21	7	3	4	7	6	7	5	10	14	6	4	9	7	6	5	5	18	7
Poisonings	18	31	24	46	30	34	32	50	23	57	23	20	31	26	48	54	31	33	58	31	56
Liver cancer	19	40	37	63	44	42	59	61	48	54	30	46	55	67	91	62	71	51	63	40	27
Other transport injuries	20	20	12	36	33	10	17	41	9	44	17	10	28	22	21	23	13	28	16	14	55

Supplementary Figure 4

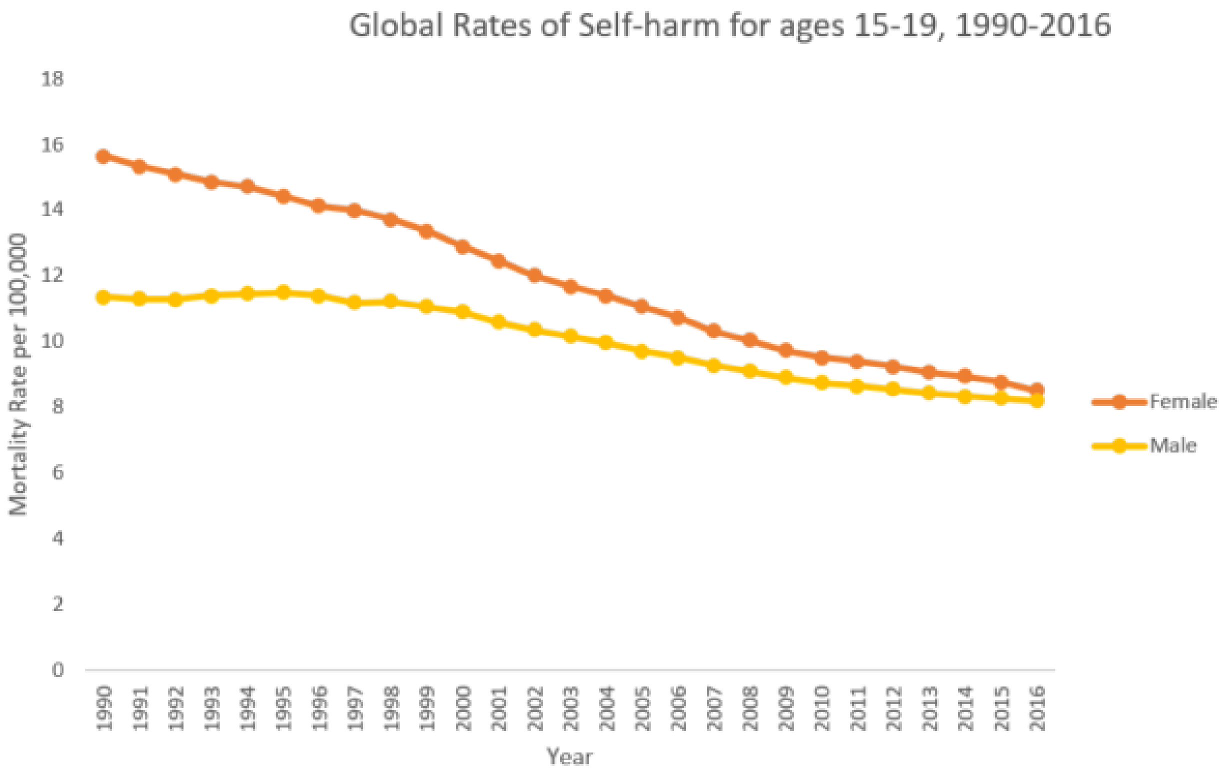

Supplementary Figure 5. Percent of all self-harm deaths that occurred in age groups 15 to 29 years, 30 to 59 years, and 60 years and above – globally and an in each GBD super-region for both sexes combined in 2016.

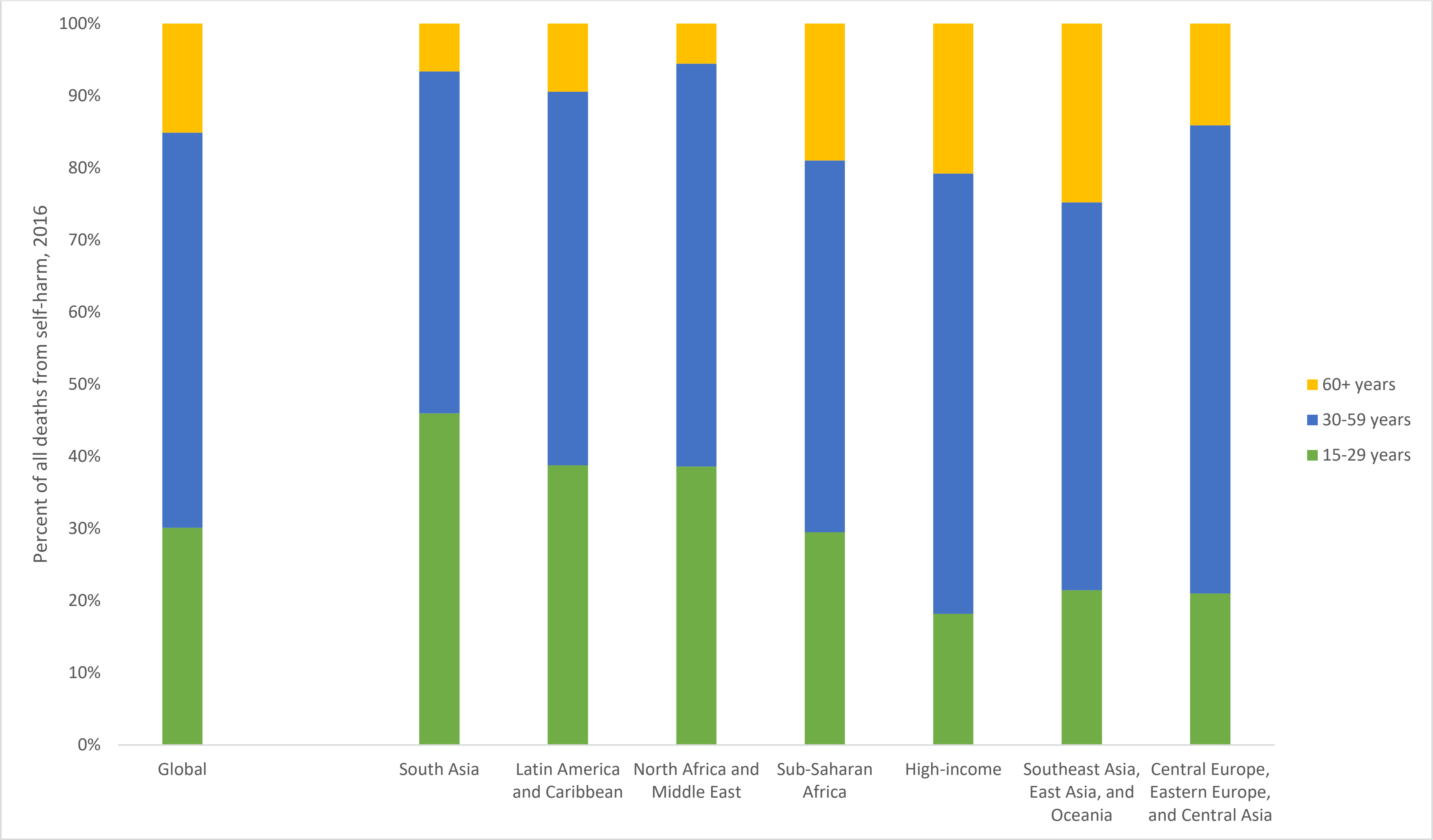

SR Table 1. Total number of deaths, age-standardized rates of death in males, age-standardized rates of death in females, age-standardized rates of death for both sexes, female/male age-standardized death rates ratio, and the percent change in the rate of death for all reporting locations in 2016

Location Name	Cause of Death Star Rating	Deaths						YLLs					
		Number	Male ASR	Female ASR	Both sexes ASR	Percent change 1990-2016	Female:Male ASR Ratio	Number	Male ASR	Female ASR	Both sexes ASR	Percent change 1990-2016	Female:Male ASR Ratio
Southeast Asia, East Asia, and Oceania		182000 (172000 to 204000)	11.1 (10.1 to 12.9)	5.8 (5.4 to 6.2)	8.4 (7.9 to 9.3)	-58.6 (-61.6 to -50.1)	0.5	6610000 (6280000 to 7600000)	382.5 (350.6 to 463.7)	199.2 (187.5 to 214.4)	291.6 (277.4 to 335.2)	-63.7 (-65.9 to -63.1)	0.5
East Asia		138000 (129000 to 154000)	10.9 (9.8 to 12.8)	6.6 (6.1 to 7.1)	8.7 (8.2 to 9.7)	-63 (-65.7 to -55.8)	0.6	4660000 (4420000 to 5330000)	353.7 (323.4 to 430.8)	223.8 (208.7 to 241.1)	289.6 (274.4 to 330.3)	-68.8 (-70.7 to -68.3)	0.6
China	★★★☆☆	131000 (122000 to 145000)	10.7 (9.5 to 12.6)	6.5 (6 to 7)	8.5 (8 to 9.5)	-64.1 (-66.9 to -57.1)	0.6	4390000 (4160000 to 5040000)	343 (313.5 to 419)	219.2 (202.9 to 237.3)	281.9 (267.3 to 324.2)	-70.1 (-72 to -69.6)	0.6
North Korea	☆☆☆☆☆	3240 (2440 to 4360)	16.8 (11.9 to 24.2)	8.9 (6.3 to 11.9)	12.2 (9.2 to 16.3)	-4.2 (-28.9 to 27.9)	0.5	132000 (97600 to 177000)	629.5 (426.4 to 900.1)	368.4 (259.7 to 488.1)	491.8 (363.8 to 659.9)	-5 (-31.3 to -4.9)	0.6
Taiwan	★★★☆☆	4050 (3370 to 4760)	18.9 (14.2 to 23.1)	8.6 (6.7 to 10.5)	13.6 (11.2 to 16)	-12.5 (-29.1 to 5.6)	0.5	145000 (114000 to 171000)	699.1 (482.1 to 863.9)	331.8 (259.8 to 402.8)	513.8 (405.8 to 607.4)	-9.1 (-27.7 to -9.2)	0.5
Southeast Asia		42400 (38600 to 49800)	10.3 (9.1 to 12.5)	3.6 (3.3 to 4)	6.8 (6.3 to 8)	-34 (-41.6 to -17.3)	0.3	1860000 (1690000 to 2230000)	420.1 (369.5 to 516.8)	134.9 (121.7 to 152.1)	276.1 (250.6 to 329.2)	-37.7 (-44.1 to -36.5)	0.3
Cambodia	★☆☆☆☆	923 (758 to 1080)	10.8 (8.5 to 12.9)	3 (2.5 to 3.6)	6.6 (5.5 to 7.6)	-11.5 (-29 to 12.3)	0.3	44500 (36000 to 53400)	455.1 (355.1 to 554.9)	117.1 (94.4 to 144.4)	278.8 (226.7 to 329.4)	-13.2 (-31.9 to -12.6)	0.3
Indonesia	★★☆☆☆	8580 (7800 to 10300)	5.3 (4.7 to 6.7)	1.8 (1.7 to 2)	3.5 (3.2 to 4.2)	-13.4 (-25.2 to 1.7)	0.3	402000 (363000 to 492000)	232.3 (204.8 to 294.7)	68.4 (62.9 to 75)	150.6 (136.1 to 183.7)	-14.8 (-26.4 to -14)	0.3
Laos	★☆☆☆☆	659 (439 to 831)	16.7 (10.5 to 21.8)	4.9 (3.5 to 6.8)	10.6 (7.3 to 13.3)	-33.3 (-49.7 to -14.5)	0.3	34700 (22800 to 44500)	756.6 (460.3 to 1006.9)	219.1 (143.8 to 309.7)	483.7 (323.2 to 612.5)	-35 (-52.3 to -35.5)	0.3
Malaysia	★★☆☆☆	2280 (1930 to 2880)	12.7 (10.1 to 16)	4.3 (3.5 to 5.1)	8.4 (7.2 to 10.2)	-19.9 (-35.1 to -1.1)	0.3	92800 (78500 to 124000)	463.5 (373.3 to 633.9)	125.3 (102.1 to 151.5)	292.8 (248.5 to 382.7)	-20.1 (-35.8 to -20.2)	0.3
Maldives	★★☆☆☆	12.1 (8.43 to 16.2)	5.7 (4 to 7.7)	2.1 (0.9 to 3.8)	3.9 (2.7 to 5.2)	-59.1 (-72 to -36.3)	0.4	533 (364 to 733)	208.7 (144.7 to 293)	73.7 (31.6 to 139.4)	140.6 (97.5 to 189.1)	-65.5 (-76.5 to -64.3)	0.4
Mauritius	★★★★☆	115 (91.1 to 143)	13.6 (10.2 to 17.3)	3.4 (2.7 to 4.2)	8.3 (6.6 to 10.3)	-35.8 (-48.4 to -16.5)	0.3	5360 (4140 to 6610)	617.7 (440.4 to 786.9)	172.5 (136.1 to 220.2)	393.7 (303.4 to 484.1)	-36.1 (-48 to -35.6)	0.3
Myanmar	★☆☆☆☆	3010 (2480 to 3730)	6.8 (5.4 to 8.5)	5.1 (4 to 7)	5.9 (4.9 to 7.3)	-33.3 (-48.1 to -9)	0.8	138000 (112000 to 172000)	291.8 (227 to 372.5)	204.3 (157.7 to 287.3)	247.2 (200.4 to 307.5)	-36.4 (-52.2 to -36.6)	0.7
Philippines	★★★★☆	5600 (4220 to 9060)	10.2 (7.3 to 17.7)	2.8 (2.3 to 3.5)	6.4 (4.9 to 10)	-58.1 (-69.8 to -10.4)	0.3	269000 (2e+05 to 441000)	422.8 (296.8 to 758.6)	107.1 (83.7 to 135.1)	264.9 (198.4 to 431.7)	-62.4 (-70.1 to -57.3)	0.3
Sri Lanka	★★★☆☆	4980 (3770 to 6710)	40.2 (28.9 to 57.1)	9.4 (6.6 to 12.6)	23.7 (18.1 to 31.9)	-42.4 (-56.5 to -20.9)	0.2	202000 (153000 to 274000)	1550.4 (1090.4 to 2245)	427.6 (298.2 to 574.2)	958.1 (729.7 to 1292.9)	-46.9 (-59.7 to -46.1)	0.3
Seychelles	★★★☆☆	8.23 (6.71 to 11)	14.5 (11.7 to 19.7)	2.5 (2 to 3.1)	8.3 (6.9 to 10.9)	-56.1 (-65.9 to -26.6)	0.2	345 (274 to 468)	581.4 (448.6 to 818.3)	92.5 (70.4 to 121.2)	340.8 (274.4 to 458.8)	-59.4 (-67.5 to -57.6)	0.2
Thailand	★★★☆☆	8410 (6990 to 9750)	17.4 (13.6 to 20.8)	4.6 (3.7 to 5.7)	10.9 (9.1 to 12.6)	-22.7 (-36.8 to -5.3)	0.3	351000 (289000 to 409000)	772.7 (589.6 to 928.1)	179.9 (140.7 to 227.7)	474.1 (390.1 to 554.2)	-24.7 (-39.6 to -24.6)	0.2
Timor-Leste	☆☆☆☆☆	67.3 (36.8 to 95.2)	10.8 (5.4 to 16.2)	4.4 (2.8 to 6.3)	7.6 (4.4 to 10.5)	-36.2 (-63.9 to -4.6)	0.4	3310 (1710 to 4730)	462.5 (201 to 711.7)	169.1 (94.4 to 253.6)	317.6 (157.3 to 461.5)	-39.9 (-70 to -40.8)	0.4
Vietnam	★☆☆☆☆	7740 (6400 to 9200)	11.4 (8.3 to 14.1)	5.4 (4.3 to 7.1)	8.2 (6.8 to 9.7)	-28.3 (-43.6 to -6.2)	0.5	320000 (257000 to 389000)	446.2 (323.5 to 571.6)	194.4 (148.4 to 260.9)	318.2 (256.3 to 383.2)	-32.2 (-48.7 to -32.1)	0.4
Oceania		1450 (1010 to 1810)	20.5 (14.5 to 28.5)	7.5 (4.8 to 11.6)	14.1 (10.1 to 17.4)	-13 (-27.7 to 8.2)	0.4	78300 (53600 to 98600)	1013.2 (693.5 to 1409.7)	344.8 (206.5 to 547.7)	684.6 (472.7 to 858.2)	-12.6 (-28 to -12.2)	0.3
American Samoa	★★★☆☆	4.57 (3.51 to 5.58)	10.4 (7.6 to 13.1)	3.7 (2.8 to 4.8)	7.1 (5.5 to 8.5)	-13.3 (-33.2 to 10.7)	0.4	233 (179 to 284)	484.8 (358.6 to 613.5)	158.8 (117.5 to 206.3)	327.4 (252.9 to 397.8)	-12.8 (-34 to -12.7)	0.3
Federated States of Micronesia	☆☆☆☆☆	19.3 (13.3 to 25.8)	29.7 (19 to 40.8)	8.9 (5.4 to 13.5)	19.4 (12.9 to 26.2)	-12.9 (-39.5 to 24.9)	0.3	1040 (716 to 1380)	1408.3 (894.8 to 1932.4)	392.9 (226.6 to 615.9)	915.1 (604.3 to 1241)	-13.4 (-39.8 to -13)	0.3
Fiji	★★☆☆☆	108 (75.5 to 145)	17.9 (11.4 to 25.7)	7.3 (4.9 to 10.4)	12.6 (8.9 to 16.9)	-2.8 (-34.3 to 49.8)	0.4	5480 (3860 to 7370)	850 (528.8 to 1245.4)	332.4 (216.3 to 485.1)	596.3 (420.2 to 797.8)	-2.9 (-34.7 to -2.5)	0.4
Guam	★★★☆☆	33.4 (26.6 to 40.5)	32.2 (24.3 to 39.9)	6.9 (5.3 to 8.6)	19.5 (15.6 to 23.6)	-1 (-22.7 to 23.2)	0.2	1580 (1240 to 1910)	1496.3 (1111.5 to 1858.7)	287.7 (217.6 to 364.9)	903.2 (710.6 to 1102.3)	0.2 (-23.9 to 0.1)	0.2
Kiribati	★★☆☆☆	26.7 (20.5 to 34.1)	43.4 (31.3 to 58.1)	7.9 (6 to 10.2)	24.9 (19.3 to 32.1)	1 (-23.6 to 37.8)	0.2	1460 (1110 to 1860)	2138.2 (1538.8 to 2830.8)	361.5 (266.8 to 474.5)	1232.1 (943.4 to 1571.2)	2 (-22.7 to 2.8)	0.2
Marshall Islands	☆☆☆☆☆	11.6 (8.03 to 14.5)	26.4 (17.6 to 35.2)	7.4 (4.4 to 11.1)	16.9 (12 to 21.2)	-15.7 (-37.5 to 11.4)	0.3	634 (436 to 798)	1309.3 (847.6 to 1738.5)	348.1 (202.1 to 529.8)	837 (580 to 1050.3)	-13.7 (-36.8 to -13.4)	0.3
Northern Mariana Islands	★★★☆☆	13 (8.63 to 17.1)	19.4 (13.7 to 25.8)	3.2 (2.3 to 4.3)	11.3 (8.2 to 14.5)	17.5 (-19.2 to 73.7)	0.2	659 (438 to 875)	804.2 (537.2 to 1079.5)	125.6 (89.3 to 169.4)	460 (322.5 to 600.2)	10.3 (-26.7 to 10.4)	0.2
Papua New Guinea	★☆☆☆☆	1070 (680 to 1420)	21.9 (14.6 to 33.2)	8.4 (4.7 to 13.9)	15.2 (10.1 to 20)	-18.2 (-35.6 to 7.6)	0.4	58500 (36400 to 77900)	1078.7 (691.5 to 1608.8)	380.6 (199.4 to 656)	736.3 (468.8 to 980.8)	-18.5 (-37 to -18.3)	0.4
Samoa	☆☆☆☆☆	21.7 (16.8 to 26.7)	19.9 (14.8 to 25.3)	5.7 (4.3 to 7.2)	12.9 (10 to 15.8)	-28.6 (-45.6 to -5)	0.3	1100 (830 to 1390)	924.4 (672.6 to 1184.5)	242.2 (172.1 to 314.3)	597.1 (455.6 to 738.1)	-30.1 (-48 to -29.9)	0.3
Solomon Islands	☆☆☆☆☆	91.5 (60.2 to 121)	25.7 (17.3 to 36.5)	10 (5.8 to 16.1)	17.8 (12.1 to 23.4)	-9.4 (-31.2 to 23.2)	0.4	4970 (3260 to 6570)	1246.9 (813.1 to 1777.6)	450.1 (249.8 to 741.1)	849.8 (558 to 1121.6)	-7.5 (-31.5 to -7.7)	0.4
Tonga	★☆☆☆☆	7.42 (5.77 to 9.13)	10.5 (7.8 to 13.4)	5 (3.7 to 6.6)	7.8 (6.1 to 9.5)	1.3 (-23.6 to 33.1)	0.5	382 (288 to 476)	510.7 (372.1 to 660.2)	209.1 (148.9 to 280.7)	361.8 (276.9 to 448.9)	5.5 (-22.9 to 5)	0.4
Vanuatu	☆☆☆☆☆	41.1 (28.7 to 53.7)	25.2 (17.6 to 34.7)	8.7 (5.4 to 13.8)	16.9 (12.1 to 21.8)	-9.2 (-30.8 to 20.8)	0.3	2210 (1520 to 2880)	1234 (848.3 to 1696.7)	394.4 (236.3 to 632.6)	811.5 (564.8 to 1062.4)	-6.9 (-30.5 to -7.2)	0.3
Central Europe, Eastern Europe, and Central Asia		98200 (78100 to 127000)	36.7 (28.4 to 48.7)	6.6 (5.1 to 8.6)	20.6 (16.4 to 26.5)	-6.8 (-23.9 to 13.8)	0.2	3970000 (3140000 to 5050000)	1534 (1171.6 to 2022.7)	273.8 (211.4 to 361.1)	886.8 (699.3 to 1116.3)	-3.2 (-21.8 to -2.9)	0.2
Central Asia		10900 (8760 to 12700)	21.1 (16.2 to 25.2)	4.8 (4.2 to 5.5)	12.5 (10.2 to 14.5)	-3.3 (-16.5 to 10.9)	0.2	528000 (418000 to 617000)	946.1 (704.9 to 1133.6)	224 (196.9 to 253.9)	575.1 (457.5 to 671.3)	2.2 (-14.2 to 1.8)	0.2
Armenia	★★★★☆	252 (161 to 308)	13.1 (6.7 to 16.7)	2.8 (2.3 to 3.4)	7.4 (4.7 to 9)	78.8 (-13.2 to 139.8)	0.2	9490 (5970 to 11700)	522.8 (278.8 to 667.6)	94.7 (78.8 to 115.2)	290.5 (182.4 to 357.7)	89.8 (-24.3 to 67.2)	0.2
Azerbaijan	★★★☆☆	423 (287 to 566)	7 (4.3 to 9.9)	1.4 (1.1 to 1.9)	4.1 (2.8 to 5.5)	22.5 (-18 to 67.4)	0.2	19800 (13300 to 26800)	316.3 (188.2 to 446.3)	57.4 (42.1 to 77.2)	185.6 (125.5 to 250)	30.7 (-19.4 to 28.9)	0.2
Georgia	★★★★☆	310 (231 to 396)	12.3 (8.5 to 16.2)	1.9 (1.4 to 2.5)	6.7 (5 to 8.6)	22.7 (-13.6 to 63.6)	0.2	11900 (8900 to 15500)	501.3 (354.5 to 678.7)	72.3 (53.2 to 96.9)	280.5 (207.8 to 368.9)	27.7 (-12.4 to 25.7)	0.1
Kazakhstan	★★★★☆	4460 (3410 to 5820)	44.2 (32.5 to 59.6)	8.3 (6.6 to 10.8)	25 (19.2 to 32.5)	1.6 (-20.8 to 30.3)	0.2	205000 (156000 to 268000)	1928.1 (1410.3 to 2625.6)	372.1 (289.5 to 483.1)	1119.9 (858.9 to 1462.7)	4.3 (-19.8 to 4.9)	0.2
Kyrgyzstan	★★★★☆	672 (552 to 917)	21.1 (16.6 to 30.7)	4.5 (3.9 to 5.3)	12.4 (10.2 to 16.9)	-27.7 (-39.1 to -8.7)	0.2	32000 (26400 to 43500)	902.5 (705.5 to 1297.3)	196.1 (166 to 232.2)	541 (444.6 to 735)	-26.9 (-37.4 to -25.9)	0.2
Mongolia	★★☆☆☆	626 (478 to 773)	34.7 (26.7 to 43.4)	6.2 (4.7 to 7.9)	20.1 (16.1 to 24.6)	15.6 (-11.1 to 47)	0.2	32600 (23600 to 40600)	1695.6 (1202.2 to 2139.3)	308.4 (230.8 to 399.3)	998.1 (744.3 to 1230.5)	14.5 (-11.2 to 14.7)	0.2
Tajikistan	★★★☆☆	396 (313 to 505)	8.3 (6.3 to 10.9)	2.4 (1.8 to 3.3)	5.3 (4.3 to 6.8)	-25.7 (-41.9 to -2.1)	0.3	19900 (15600 to 25500)	368 (277.5 to 485.3)	103.6 (75.9 to 144)	235.7 (186.5 to 301.5)	-25.3 (-41.8 to -24.9)	0.3
Turkmenistan	★★★★☆	467 (355 to 546)	13.7 (10.1 to 17.1)	3.4 (3 to 4)	8.4 (6.7 to 10)	-15.9 (-27.1 to 2)	0.2	24500 (17900 to 28700)	660.5 (447.1 to 793.3)	169.7 (146.7 to 199.6)	410.7 (307 to 477)	-11.2 (-22.3 to -10.8)	0.3
Uzbekistan	★★★★☆	3290 (2400 to 3980)	17.1 (11.6 to 21.5)	4.9 (4 to 6)	10.8 (8.1 to 13)	4.6 (-14.4 to 26)	0.3	172000 (122000 to 207000)	815.2 (514.2 to 1024.1)	256.9 (210.9 to 309.9)	529.2 (379.5 to 634.1)	16.4 (-5.4 to 16.4)	0.3
Central Europe		18900 (15900 to 21300)	22.6 (17.9 to 25.9)	4.2 (3.9 to 4.5)	13 (10.8 to 14.5)	-23.3 (-32.7 to -14.5)	0.2	671000 (550000 to 746000)	882.2 (687.8 to 994.6)	155.8 (145.1 to 168.4)	517 (420.4 to 574.9)	-22.6 (-34.5 to -23)	0.2
Albania	★★★☆☆	181 (137 to 220)	7.9 (5.2 to 10.2)	3.5 (2.8 to 4.5)	5.7 (4.2 to 6.9)	33.6 (-3.4 to 68.2)	0.4	7990 (5790 to 9760)	354.3 (222.5 to 460.1)	166.4 (131.3 to 213.1)	258.6 (186.6 to 317)	36.3 (-4.3 to 34.8)	0.5
Bosnia and Herzegovina	★★☆☆☆	352 (273 to 459)	12.2 (9 to 16.6)	2.7 (2.1 to 3.4)	7.3 (5.7 to 9.5)	-40.4 (-55.7 to -20.4)	0.2	12500 (9760 to 16300)	474 (357.8 to 639)	104.6 (81.1 to 134.2)	291.1 (228.9 to 375)	-45.9 (-59.6 to -45.8)	0.2
Bulgaria	★★★★☆	1080 (844 to 1490)	18.1 (13.4 to 27)	4 (3.1 to 5.1)	10.7 (8.2 to 15.2)	-33.4 (-48.3 to -10.7)	0.2	33300 (25200 to 49000)	651.9 (465.2 to 1022.3)	139.9 (109.8 to 182)	395.9 (297.3 to 590.8)	-32.8 (-47.8 to -31.5)	0.2
Croatia	★★★★☆	825 (679 to 1070)	24.1 (18.8 to 33.1)	5.7 (4.6 to 7)	14.3 (11.7 to 18.9)	-37.8 (-50 to -16.1)	0.2	25700 (20900 to 34200)	870.8 (674.2 to 1229.7)	200.1 (159.2 to 244)	530.2 (429.2 to 711.1)	-40.3 (-51.6 to -39.2)	0.2
Czech Republic	★★★★☆	1560 (1300 to 2060)	20 (15.5 to 27)	3.8 (3.3 to 4.2)	11.6 (9.4 to 14.8)	-40.4 (-51 to -22.9)	0.2	54200 (42500 to 69000)	770.2 (564.5 to 1000.9)	140.4 (123.8 to 157.2)	456.5 (351.9 to 572.2)	-39.5 (-51.4 to -38.8)	0.2
Hungary	★★★★★	2460 (2040 to 3340)	31.5 (24.9 to 46)	7.4 (6.3 to 8.7)	18.5 (15.2 to 25.4)	-48.1 (-58.2 to -24.4)	0.2	79700 (65400 to 110000)	1119.4 (877.7 to 1650.2)	253.3 (214.4 to 298.4)	675.3 (551.5 to 924.3)	-51.9 (-60.4 to -50.5)	0.2
Macedonia	★★★☆☆	207 (177 to 243)	13.5 (11 to 16.5)	3.8 (3.2 to 4.4)	8.4 (7.2 to 9.9)	-16.5 (-29.4 to -0.5)	0.3	7180 (6250 to 8760)	480.4 (397.9 to 604.4)	134.8 (114.4 to 156.7)	306.8 (267.5 to 374.2)	-16.1 (-28.4 to -16.1)	0.3
Montenegro	★★☆☆☆	82.2 (70.3 to 96)	16.9 (13.7 to 20.4)	5.8 (4.7 to 6.9)	11.1 (9.6 to 12.9)	7.7 (-9.9 to 28.9)	0.3	2930 (2520 to 3370)	647.3 (533.4 to 776.3)	217.7 (176.4 to 261.2)	431.2 (370.5 to 498.6)	3.5 (-13.6 to 3.1)	0.3
Poland	★★★★☆	6830 (4840 to 8180)	26.2 (17.1 to 32)	3.7 (3.1 to 4.3)	14.6 (10.1 to 17.5)	-0.6 (-26.9 to 19.6)	0.1	262000 (178000 to 314000)	1080.2 (666.4 to 1327.3)	146.1 (123.5 to 171.6)	610.7 (408.4 to 732.6)	-2.4 (-29.8 to -3.8)	0.1
Romania	★★★★☆	2420 (1760 to 2850)	17.7 (11.8 to 21.5)	2.8 (2.4 to 3.3)	10 (7.2 to 11.8)	1.5 (-22.1 to 20.8)	0.2	87400 (61800 to 104000)	712.8 (463.7 to 871.4)	108.9 (92.5 to 129.2)	409.2 (286.5 to 486.4)	1.9 (-26.3 to 0.7)	0.2
Serbia	★★★☆☆	1720 (1550 to 1990)	25.4 (22.2 to 30.6)	6.5 (5.8 to 7.4)	15.5 (13.9 to 18.2)	-19.6 (-31.3 to -5.2)	0.3	57300 (51300 to 68400)	954.5 (829.2 to 1180.9)	231.9 (203 to 264.8)	590 (523.6 to 711.2)	-19.6 (-31.8 to -19.5)	0.2
Slovakia	★★★☆☆	714 (586 to 861)	19 (15 to 23.5)	3 (2.4 to 3.5)	10.6 (8.6 to 12.8)	-35 (-47 to -14.6)	0.2	26500 (21200 to 31900)	736.1 (560.2 to 905.4)	113 (91.8 to 136.6)	422.4 (339.4 to 509.6)	-37.8 (-49 to -36.7)	0.2
Slovenia	★★★★☆	453 (365 to 608)	27.7 (21.4 to 38.9)	5.3 (4.3 to 6.5)	16.1 (13 to 21.9)	-46.6 (-58 to -22.9)	0.2	14400 (11500 to 19800)	1005.3 (773.1 to 1432.2)	187.1 (151.2 to 229)	601.2 (482.6 to 823.5)	-50.4 (-60.2 to -49.2)	0.2
Eastern Europe		68400 (50200 to 94200)	50 (34.8 to 71.1)	8.3 (5.5 to 12.2)	27.5 (20.1 to 37.2)	1.4 (-24.1 to 34.4)	0.2	2770000 (2e+06 to 3770000)	2118.2 (1455.2 to 3016.8)	346.4 (225.9 to 517.2)	1200.3 (869.2 to 1635.9)	5.5 (-22.5 to 5.8)	0.2
Belarus	★★★★☆	2660 (2070 to 3540)	42.7 (31.2 to 59.1)	6.5 (4.9 to 8.5)	23 (17.8 to 30.6)	-0.1 (-21.5 to 26.4)	0.2	103000 (79000 to 138000)	1717.1 (1252.6 to 2391.3)	257.6 (190.7 to 337.6)	957.6 (738.7 to 1289.5)	-2.6 (-23.3 to -1.6)	0.2
Estonia	★★★★★	256 (200 to 382)	28.8 (21.8 to 45.7)	4 (3.1 to 5)	15.5 (12.1 to 23.5)	-44.3 (-56.2 to -18.8)	0.1	9020 (6990 to 13900)	1135.1 (851.8 to 1818.7)	146.3 (112.7 to 183.5)	632.1 (490.8 to 971.5)	-47.9 (-58.6 to -46.3)	0.1
Latvia	★★★★★	482 (388 to 654)	36.5 (28.1 to 51.4)	4.9 (4 to 6.1)	19.4 (15.5 to 26.7)	-31.1 (-44.7 to -11.4)	0.1	17200 (13600 to 23700)	1450.8 (1091.4 to 2058.6)	179.2 (144.2 to 224.2)	794 (624.2 to 1098.9)	-32.4 (-45.5 to -31.4)	0.1
Lithuania	★★★★★	1090 (910 to 1280)	56.6 (44.4 to 67.7)	8.7 (7.6 to 10)	31 (25.5 to 36.2)	2.4 (-11.8 to 17)	0.2	41100 (33600 to 48100)	2355.3 (1835.5 to 2821.1)	341.3 (294.6 to 392.3)	1317.8 (1065.1 to 1547.5)	3.9 (-14.5 to 3.6)	0.1
Moldova	★★★★★	728 (581 to 895)	27.6 (21 to 34.7)	4.2 (3.4 to 5)	15.1 (12 to 18.6)	-22.5 (-36.3 to -6.6)	0.2	28700 (22700 to 36000)	1089.1 (821.1 to 1404.5)	160 (131.2 to 192)	607.4 (483.1 to 764.6)	-25.5 (-38.9 to -25.1)	0.1
Russian Federation	★★★★★	52000 (35200 to 75100)	55.3 (34.9 to 85)	9.6 (5.6 to 15.3)	30.6 (20.6 to 43.6)	4.4 (-30 to 48.9)	0.2	2140000 (1420000 to 3060000)	2368.2 (1476.5 to 3590.2)	402.5 (231.7 to 653.1)	1349.5 (889.7 to 1922.4)	8.9 (-27.4 to 9.6)	0.2
Ukraine	★★★★★	11200 (7870 to 16300)	37.9 (25.1 to 56.7)	5.4 (3.4 to 8)	20.2 (14 to 29.1)	-7.9 (-33.4 to 25.2)	0.1	436000 (299000 to 632000)	1551.1 (1009.1 to 2304)	216.2 (134.9 to 323.6)	857.6 (589.7 to 1216.1)	-5.7 (-31.9 to -4.4)	0.1
High-income		161000 (140000 to 184000)	19.3 (16.2 to 23)	6 (5.7 to 6.4)	12.5 (11 to 14.4)	-11.9 (-25 to -5.4)	0.3	5810000 (5110000 to 6670000)	789.2 (679.2 to 945)	249.1 (235.6 to 264.7)	518.6 (461.6 to 597.1)	-9.6 (-26.4 to -11.1)	0.3
High-income Asia Pacific		45600 (38000 to 53100)	26.9 (20.1 to 32.6)	11 (9.3 to 12.9)	18.7 (15.6 to 21.7)	10.1 (-23.5 to 30)	0.4	1480000 (1230000 to 1710000)	1034.7 (774 to 1248.3)	447.5 (377.6 to 533.3)	742 (614.6 to 855.6)	20.8 (-22.4 to 16.8)	0.4
Brunei	★★★☆☆	20.6 (15 to 27.8)	6.9 (4.5 to 9.7)	2.5 (1.8 to 3.3)	4.8 (3.5 to 6.2)	-2.7 (-31.7 to 36.6)	0.4	936 (671 to 1300)	289.9 (181 to 431.8)	99.5 (71.2 to 136.6)	197.7 (142.9 to 269.5)	-4 (-34.4 to -3.2)	0.3
Japan	★★★★☆	29000 (25300 to 32900)	25.1 (19.7 to 28.4)	9.7 (8.9 to 10.6)	17.3 (14.7 to 19)	-0.8 (-19.2 to 8.7)	0.4	926000 (788000 to 1020000)	998.1 (777.2 to 1119.5)	397.5 (368.1 to 432.2)	699.7 (592.2 to 764.7)	12.1 (-13.5 to 9.7)	0.4

South Korea	★★★☆☆	16100 (10400 to 21800)	36.3 (18.8 to 53.4)	15.5 (10 to 22.7)	25.1 (16.5 to 33.7)	77.3 (-29.9 to 172.3)	0.4	538000 (364000 to 728000)	1221.8 (707.6 to 1778.6)	588.5 (376.7 to 878.4)	899 (617.5 to 1209.6)	66 (-37 to 53.4)	0.5
Singapore	★★★★★	398 (306 to 565)	12.2 (8.7 to 18.9)	4.8 (3.5 to 6.4)	8.4 (6.5 to 11.7)	-50.6 (-62.9 to -31)	0.4	14100 (10700 to 20600)	453.1 (318.8 to 708.1)	182.2 (132.3 to 239.9)	314.6 (240.3 to 448.3)	-52.1 (-63.5 to -51.4)	0.4
Australasia		3360 (2830 to 3830)	16.4 (13 to 19.2)	5 (4.5 to 5.5)	10.6 (8.9 to 12)	-19.9 (-31.7 to -8.3)	0.3	141000 (119000 to 159000)	729.8 (593.9 to 856.9)	225.5 (202.9 to 251.5)	477.2 (409 to 539.6)	-21.4 (-33.5 to -21.4)	0.3
Australia	★★★★★	2830 (2380 to 3260)	16.2 (12.9 to 19.2)	4.9 (4.3 to 5.5)	10.5 (8.9 to 12.1)	-20 (-32.5 to -8.5)	0.3	117000 (99600 to 135000)	712.7 (574 to 846.7)	217.7 (191 to 247.1)	465.6 (396.7 to 539.6)	-21.8 (-33.9 to -21.7)	0.3
New Zealand	★★★★★	536 (436 to 633)	17.2 (13 to 21.2)	5.4 (4.6 to 6.4)	11.1 (9 to 13.1)	-19.6 (-34.3 to -3.6)	0.3	23800 (19000 to 27800)	816.6 (608.7 to 997.6)	264.4 (221.8 to 311.5)	535.5 (428.3 to 628.7)	-19.6 (-34.8 to -19.5)	0.3
Western Europe		52600 (46600 to 65100)	15.2 (13.4 to 20.8)	4.3 (4.1 to 4.6)	9.6 (8.7 to 12.4)	-31.4 (-38 to -20)	0.3	1790000 (1620000 to 2350000)	602.6 (532.4 to 859.4)	167.5 (158.5 to 178.1)	384.1 (347.2 to 514)	-32.4 (-39.8 to -32.2)	0.3
Andorra	☆☆☆☆☆	7.84 (5.61 to 10.7)	14 (9.3 to 20.1)	2.7 (1.7 to 4.1)	8.3 (6 to 11.2)	-15 (-43.1 to 32.1)	0.2	302 (216 to 411)	604.9 (399.5 to 860.4)	118.4 (75.2 to 171.7)	362.4 (259.4 to 489.6)	-16.1 (-44.9 to -16)	0.2
Austria	★★★★★	1470 (1280 to 1990)	20.5 (17.2 to 31)	5.4 (4.8 to 6)	12.6 (10.9 to 18)	-42.6 (-50.2 to -25.9)	0.3	47800 (41100 to 71200)	769.9 (633.5 to 1254.8)	198.3 (175.4 to 224.2)	482.5 (412.1 to 732)	-46.6 (-52.8 to -45.4)	0.3
Belgium	★★★★☆	2250 (1920 to 2720)	23.9 (19.5 to 31.5)	8.5 (7.1 to 10)	16 (13.7 to 19.8)	-18 (-33.5 to -5.6)	0.4	78900 (67300 to 98500)	953.5 (785.6 to 1278.8)	325.5 (271.7 to 383.3)	640 (545.5 to 814.7)	-18.5 (-36.2 to -19.1)	0.3
Cyprus	★★☆☆☆	50.9 (45.1 to 61.5)	7.8 (6.7 to 9.8)	1.9 (1.7 to 2.1)	4.7 (4.1 to 5.8)	-22.3 (-35.2 to -7.4)	0.2	1930 (1690 to 2520)	316.9 (269.4 to 438.1)	67.7 (58.6 to 77.1)	187.3 (163.6 to 246.6)	-17.9 (-31.7 to -18.2)	0.2
Denmark	★★★★☆	730 (602 to 1060)	15.7 (12.3 to 25.9)	4.6 (3.8 to 5.5)	10 (8.1 to 15.1)	-60 (-68.3 to -37.6)	0.3	24200 (19500 to 37400)	595.1 (456.8 to 1045)	163.1 (133.5 to 197.8)	379.8 (305.5 to 605.6)	-63.1 (-69.3 to -61.1)	0.3
Finland	★★★★★	907 (762 to 1280)	22.3 (17.9 to 33.5)	6 (5.1 to 6.9)	14 (11.9 to 19.6)	-50 (-58.7 to -23.8)	0.3	34000 (28700 to 47100)	946.5 (768.5 to 1386)	260.8 (224.1 to 300.1)	607.7 (516.5 to 830.2)	-53.4 (-60.1 to -51.8)	0.3
France	★★★★☆	12400 (10900 to 15900)	24.7 (20.8 to 34.6)	6.8 (6 to 7.5)	15.3 (13.4 to 20.4)	-34.1 (-43 to -20.7)	0.3	412000 (359000 to 560000)	954.6 (794.6 to 1417)	256.3 (226.5 to 285)	599.4 (517.7 to 831.9)	-34.8 (-43.3 to -34.1)	0.3
Germany	★★★★☆	12000 (10300 to 15400)	16.7 (13.8 to 24.2)	4.7 (4.1 to 5.5)	10.5 (9.1 to 14.3)	-30.5 (-41.6 to -16.9)	0.3	379000 (326000 to 527000)	636.3 (524.9 to 997.4)	175.4 (149 to 202.9)	406.4 (346.4 to 583.8)	-32.7 (-43.9 to -32.2)	0.3
Greece	★★★★☆	513 (424 to 624)	6.4 (5.2 to 8.8)	1.2 (1 to 1.4)	3.7 (3.1 to 5)	-3.9 (-20.3 to 11.4)	0.2	17900 (15100 to 24700)	265.6 (213.2 to 400.8)	48.9 (42.1 to 56)	157.1 (130.9 to 227.1)	-1.5 (-18.5 to -2.1)	0.2
Iceland	★★★★★	41.5 (35.9 to 50.8)	17.7 (14.8 to 22.5)	4.2 (3.5 to 4.8)	10.8 (9.3 to 13.4)	-29.2 (-40 to -18)	0.2	1480 (1290 to 1920)	693.9 (585.4 to 942.8)	147.9 (125.3 to 172.7)	422.7 (366.3 to 551.6)	-31.6 (-42.3 to -31.4)	0.2
Ireland	★★★★★	482 (348 to 594)	16.4 (10.8 to 20.8)	3.7 (3 to 4.5)	9.9 (7.1 to 12.2)	-7.5 (-28.5 to 13.8)	0.2	20900 (14900 to 25800)	760 (490.4 to 965.2)	167.4 (134.8 to 204.7)	460.8 (325.2 to 568.8)	-5.1 (-25.9 to -5.2)	0.2
Israel	★★★★☆	628 (472 to 868)	13.2 (9.4 to 19.6)	2.5 (1.9 to 3.4)	7.7 (5.8 to 10.8)	-21.8 (-42.3 to 1.7)	0.2	25400 (18900 to 36700)	554.8 (393.7 to 854.3)	95.5 (69.5 to 129.2)	324.3 (241.5 to 470)	-16.7 (-38.8 to -16.7)	0.2
Italy	★★★★★	4700 (3950 to 6070)	9.4 (7.5 to 14.1)	2.2 (1.9 to 2.5)	5.7 (4.7 to 8)	-29.9 (-43.4 to -17)	0.2	150000 (124000 to 218000)	365.2 (287.3 to 603.6)	84.7 (73.6 to 97.1)	223.8 (183.9 to 342.2)	-28.4 (-43.5 to -28.8)	0.2
Luxembourg	★★★★☆	68.2 (56.6 to 97.4)	14.1 (10.9 to 22.7)	4.9 (4.1 to 5.8)	9.4 (7.8 to 13.7)	-46.4 (-57.4 to -25.9)	0.3	2350 (1940 to 3590)	524.5 (399 to 906.7)	178.8 (150 to 210.5)	353.2 (289.8 to 543.7)	-51.8 (-60.5 to -50.3)	0.3
Malta	★★★★★	24.3 (19.1 to 32.8)	8.1 (6 to 11.5)	1.3 (1 to 1.7)	4.7 (3.6 to 6.6)	-17.1 (-35.6 to 6.2)	0.2	868 (656 to 1280)	326.4 (235.7 to 514)	51.9 (38 to 67.5)	192.8 (145.2 to 293.3)	-13.4 (-33.4 to -13.1)	0.2
Netherlands	★★★★☆	1960 (1660 to 2300)	13.3 (10.5 to 17)	5.3 (4.5 to 6.1)	9.2 (7.8 to 11.1)	-22.6 (-36.6 to -11.7)	0.4	69000 (58500 to 83500)	533.6 (426.7 to 706.7)	205.8 (177.8 to 238.2)	369.5 (314.8 to 456.4)	-22.7 (-38.5 to -23.3)	0.4
Norway	★★★★★	591 (499 to 752)	14.3 (11.5 to 19.6)	5.3 (4.4 to 6.3)	9.8 (8.3 to 12.4)	-34.7 (-44.8 to -15.5)	0.4	23400 (19700 to 29400)	619.3 (492.9 to 819.9)	226.5 (186.3 to 273.1)	427.4 (362.5 to 530.6)	-37 (-45.9 to -36)	0.4
Portugal	★★★★☆	1430 (1140 to 1650)	16.9 (13.8 to 21)	3.5 (3 to 4)	9.7 (8.3 to 12)	-31.4 (-43.6 to -17.5)	0.2	43600 (37100 to 56400)	619.6 (503 to 899.8)	121.7 (105.8 to 138.4)	360.6 (302.2 to 502.8)	-37.4 (-48.4 to -36.9)	0.2
Spain	★★★★☆	3760 (3190 to 4630)	9.6 (8 to 13.4)	2.7 (2.4 to 3)	6 (5.2 to 7.9)	-24.4 (-44.5 to -11.4)	0.3	123000 (105000 to 168000)	360.2 (294.9 to 557)	101.3 (89.2 to 114.6)	230.1 (195.9 to 329.3)	-24.3 (-50.4 to -26.6)	0.3
Sweden	★★★★★	1430 (1140 to 1670)	17.2 (12.8 to 21.5)	6.9 (5.8 to 8.4)	12.1 (9.7 to 14.2)	-35 (-45 to -20.3)	0.4	51900 (41700 to 61300)	710.7 (539.6 to 888.6)	291.9 (242.9 to 355.2)	504.4 (411.5 to 599.5)	-35.2 (-44.8 to -34.7)	0.4
Switzerland	★★★★☆	1310 (963 to 1890)	18.2 (12.1 to 29.6)	5.9 (4 to 8.4)	11.8 (8.6 to 17.8)	-50.3 (-63.5 to -27.9)	0.3	43100 (31100 to 67400)	686.1 (447.2 to 1158.2)	217.3 (146.8 to 316.1)	452.1 (325.3 to 707)	-55.9 (-67.2 to -54.3)	0.3
United Kingdom	★★★★★	5800 (4890 to 6410)	12.3 (9.6 to 13.7)	3.5 (3.4 to 3.7)	7.9 (6.6 to 8.5)	-21.7 (-27.4 to -15.6)	0.3	234000 (194000 to 253000)	546.3 (422.6 to 596.2)	153.4 (146.6 to 161.6)	349.1 (289 to 375.3)	-21.6 (-27.8 to -22.1)	0.3
Southern Latin America		7810 (6630 to 9240)	19.6 (16 to 24)	4.1 (3.6 to 4.7)	11.5 (9.7 to 13.6)	-15 (-27.3 to -1.1)	0.2	340000 (277000 to 392000)	838.8 (645.7 to 998.1)	188.1 (162.9 to 217.6)	510.2 (414.4 to 589.8)	-6.8 (-21.1 to -7.1)	0.2
Argentina	★★★★☆	5010 (4130 to 5780)	19.1 (14.9 to 22.7)	4.2 (3.7 to 4.7)	11.2 (9.2 to 12.9)	2.9 (-13.2 to 18)	0.2	223000 (176000 to 257000)	828.2 (611.3 to 973.2)	190.9 (165.9 to 218.6)	505.6 (399.9 to 582.3)	21.5 (-7.3 to 19.5)	0.2
Chile	★★★★☆	2160 (1550 to 3040)	18.8 (12.9 to 27.7)	3.5 (2.3 to 5)	10.9 (7.9 to 15.4)	-46 (-62.7 to -10.9)	0.2	92300 (66300 to 127000)	799 (543.6 to 1145.9)	163 (108.5 to 236.9)	480.1 (345.8 to 655.1)	-45 (-61.2 to -43.9)	0.2
Uruguay	★★★★☆	642 (486 to 722)	28.4 (19.6 to 32.9)	6.8 (5.9 to 7.8)	16.8 (12.8 to 18.9)	38.7 (-0.4 to 63)	0.2	24600 (19100 to 27800)	1136.2 (800.5 to 1323.6)	278.9 (241.2 to 322.3)	698.8 (546.2 to 792.1)	52.7 (3.3 to 46.3)	0.2
High-income North America		51200 (43400 to 57000)	20 (16.2 to 23)	5.8 (5.5 to 6)	12.7 (10.8 to 14.1)	-2.3 (-14.4 to 3.1)	0.3	2060000 (1740000 to 2270000)	856.4 (696.9 to 973.2)	257.6 (245.3 to 268.7)	556.4 (473.7 to 613.3)	-2 (-16.2 to -3.1)	0.3
Canada	★★★★★	4570 (3800 to 5190)	16.3 (12.4 to 19.2)	5.6 (5.1 to 6.1)	10.9 (9 to 12.3)	-17.5 (-26.4 to -2.9)	0.3	185000 (149000 to 208000)	717.9 (529.1 to 837.9)	254.8 (232.8 to 277.7)	486.1 (387.9 to 548.2)	-19.8 (-28 to -19.3)	0.4
Greenland	★★★☆☆	40.1 (31.7 to 50.5)	118.4 (89.4 to 155.6)	38.1 (28.3 to 49.2)	79.7 (63.4 to 99.5)	-42.5 (-55 to -22.7)	0.3	2010 (1590 to 2490)	5749 (4295.9 to 7513.3)	2044.2 (1529.6 to 2666.6)	3996.2 (3173.7 to 4951.5)	-45.7 (-57.6 to -45.2)	0.4
United States	★★★★★	46600 (39300 to 52000)	20.4 (16.4 to 23.6)	5.8 (5.5 to 6.1)	12.9 (10.9 to 14.4)	-0.3 (-13.7 to 5.7)	0.3	1870000 (1600000 to 2070000)	872 (712.2 to 992.2)	258 (243.8 to 270.6)	564.3 (483.3 to 623.8)	0.4 (-15.6 to -0.9)	0.3
Latin America and Caribbean		37900 (32000 to 44500)	11.1 (8.8 to 13.4)	2.4 (2.3 to 2.6)	6.6 (5.6 to 7.7)	-6.1 (-16.1 to 3.5)	0.2	1780000 (1500000 to 2110000)	482.4 (385.7 to 588.2)	114.1 (106.2 to 123.1)	295.6 (249.8 to 349.3)	-1.8 (-12.5 to -1.6)	0.2
Caribbean		4320 (3800 to 4860)	15.3 (12.9 to 17.6)	4 (3.3 to 4.7)	9.4 (8.3 to 10.6)	-24.5 (-32.9 to -15.5)	0.3	174000 (153000 to 199000)	598.1 (515.9 to 707.4)	160.9 (128.4 to 199.7)	375.9 (329.7 to 429.7)	-26.9 (-35 to -26.8)	0.3
Antigua and Barbuda	★★★★☆	2.13 (1.63 to 2.65)	3.9 (2.8 to 5.1)	0.8 (0.6 to 1)	2.3 (1.7 to 2.8)	3.7 (-19 to 31)	0.2	97.1 (73.2 to 123)	173.4 (120.9 to 228.5)	35.3 (27.7 to 44.3)	100.8 (76 to 127.3)	5 (-18.1 to 5.4)	0.2
The Bahamas	★★★★☆	13 (9.96 to 16)	5.3 (3.8 to 6.8)	1.1 (0.9 to 1.3)	3.1 (2.4 to 3.8)	8.8 (-13 to 37.3)	0.2	597 (445 to 757)	236.3 (163 to 313.9)	46.8 (38.3 to 57.4)	140 (104.4 to 177.6)	7.2 (-14 to 9.2)	0.2
Barbados	★★★★☆	15.4 (12.2 to 18.4)	8.1 (5.9 to 10.2)	2 (1.7 to 2.4)	4.9 (3.9 to 5.8)	12.1 (-9.6 to 36)	0.2	614 (490 to 736)	347.9 (252.7 to 441.1)	91 (74.6 to 111.1)	214.3 (169.5 to 259.4)	12 (-10.6 to 12.2)	0.3
Belize	★★★★☆	24.8 (18 to 32.3)	13.2 (8.8 to 17.4)	2.7 (2.1 to 3.3)	7.9 (5.8 to 10)	52.2 (8.8 to 100.7)	0.2	1200 (875 to 1600)	545.6 (359.7 to 750.5)	119.5 (92.5 to 150.6)	329.9 (240.1 to 432.4)	45.3 (4.2 to 44.6)	0.2
Bermuda	★★★★★	3 (2.37 to 4.22)	6.9 (5.2 to 10.2)	1.7 (1.3 to 2.1)	4.2 (3.3 to 5.9)	-46.8 (-59.4 to -15.5)	0.2	117 (91.1 to 178)	262 (191.5 to 426.1)	60.9 (47.1 to 77.2)	160 (124.4 to 242.1)	-52.3 (-63.2 to -50.9)	0.2
Cuba	★★★★★	1750 (1500 to 2160)	19.2 (15.8 to 25.2)	5 (4.3 to 5.8)	12 (10.2 to 15.1)	-42 (-50.6 to -28.7)	0.3	55600 (46800 to 74900)	668.9 (544.3 to 999)	168.6 (142 to 197.8)	420.4 (352.3 to 592)	-50.2 (-57.5 to -49.3)	0.3
Dominica	★★★★☆	3.51 (2.38 to 4.4)	8.1 (5 to 10.4)	1.3 (1.1 to 1.6)	4.6 (3.2 to 5.8)	36.4 (0.1 to 76)	0.2	158 (105 to 201)	353.1 (217.2 to 463.3)	58.3 (46.7 to 74.1)	204.4 (137 to 260.5)	37.9 (1 to 38.5)	0.2
Dominican Republic	★★★☆☆	570 (435 to 719)	10.2 (7.2 to 13.4)	2.1 (1.6 to 2.7)	6 (4.5 to 7.6)	17.9 (-20.7 to 60)	0.2	23500 (18200 to 29800)	378 (276.7 to 500.1)	81.3 (60.2 to 102.8)	227.3 (174.2 to 287)	14.5 (-22.4 to 12.9)	0.2
Grenada	★★★★☆	7.64 (5.69 to 9.74)	11.9 (8.4 to 15.5)	2.4 (1.9 to 3.1)	7.1 (5.4 to 8.9)	28.7 (-5 to 72.5)	0.2	370 (270 to 482)	531.9 (364.4 to 709.5)	109.5 (86.1 to 139.1)	323.1 (238.3 to 416)	26.3 (-7.9 to 27)	0.2
Guyana	★★★★☆	220 (163 to 262)	47.6 (31.7 to 58.2)	13.1 (10.7 to 15.7)	30.4 (22.8 to 36.1)	33.8 (1.2 to 63.9)	0.3	10700 (7830 to 12900)	2142.4 (1367.8 to 2636.1)	619 (500 to 746.1)	1397.2 (1021.4 to 1677)	35.8 (2.2 to 35.3)	0.3
Haiti	★☆☆☆☆	971 (673 to 1330)	14.7 (9.5 to 23)	5.4 (2.9 to 8.7)	9.8 (7 to 13.3)	-4.9 (-30.8 to 35.3)	0.4	49800 (33700 to 68700)	667.3 (416.8 to 1062.4)	248.2 (129 to 407.7)	452.3 (310.2 to 618)	-7.1 (-33.8 to -6.5)	0.4
Jamaica	★★★☆☆	84.6 (62.9 to 108)	4.8 (3.2 to 6.5)	1.1 (0.8 to 1.5)	2.9 (2.2 to 3.7)	70.9 (21 to 128.2)	0.2	3760 (2770 to 4900)	210.1 (136.3 to 288.6)	46.1 (33.4 to 61.3)	127.2 (93.8 to 164.4)	69.4 (19.8 to 69.7)	0.2
Puerto Rico	★★★★☆	314 (254 to 424)	14.1 (11 to 19.9)	1.7 (1.4 to 2)	7.5 (6 to 10.4)	-29.9 (-45.1 to -0.6)	0.1	12300 (9620 to 17900)	584.5 (436.1 to 887.7)	66.9 (55.6 to 79.8)	317 (244.2 to 469.6)	-32 (-46.4 to -29.7)	0.1
Saint Lucia	★★★★☆	13.7 (10 to 17.4)	12.5 (8.5 to 16.4)	1.9 (1.6 to 2.3)	7 (5.1 to 8.9)	3.3 (-20.7 to 35)	0.2	618 (443 to 797)	552.9 (363.4 to 736.3)	85.8 (70.1 to 104.1)	314.3 (225.9 to 405.2)	1 (-22.3 to 2.8)	0.2
Saint Vincent and the Grenadines	★★★★☆	8.49 (6.51 to 10.3)	13.2 (9.7 to 16.3)	2 (1.7 to 2.3)	7.6 (5.8 to 9.2)	31.6 (3.7 to 64.4)	0.2	389 (286 to 482)	578.9 (410.6 to 734.2)	87.4 (73.7 to 104.7)	336.2 (248.5 to 416.1)	30 (0.2 to 30.1)	0.2
Suriname	★★★☆☆	131 (105 to 150)	38.3 (29 to 44.6)	11.3 (9.5 to 13.1)	24.5 (19.6 to 28)	35.2 (5.4 to 61.8)	0.3	5960 (4700 to 6810)	1641.9 (1203.3 to 1935.1)	484.3 (401.6 to 565.9)	1063.9 (842.3 to 1216.6)	30.9 (2.4 to 29.7)	0.3
Trinidad and Tobago	★★★★★	177 (138 to 212)	20.7 (15.3 to 25.4)	3.8 (3.3 to 4.4)	12 (9.5 to 14.4)	-13.5 (-29.4 to 6.8)	0.2	8140 (6220 to 9990)	937.2 (674.5 to 1181.4)	188.3 (157.8 to 221.9)	559.9 (428.5 to 684.5)	-13.4 (-30.2 to -12.9)	0.2
Virgin Islands, U.S.	★★★☆☆	7.44 (5.5 to 9.36)	10.1 (6.7 to 13.4)	2.4 (1.6 to 3.1)	6 (4.2 to 7.6)	-2.7 (-31.2 to 31.9)	0.2	262 (183 to 330)	411.8 (254.6 to 552.2)	97.3 (58.3 to 133.6)	244.8 (158.7 to 313.3)	-7.6 (-40.2 to -8.4)	0.2
Andean Latin America		2880 (2390 to 3290)	7.4 (5.6 to 8.8)	2.9 (2.4 to 3.3)	5.1 (4.2 to 5.8)	-6.4 (-23 to 12.7)	0.4	140000 (115000 to 159000)	324.8 (243.3 to 385.6)	136.6 (116.6 to 157.4)	230 (190.4 to 262.4)	-5.8 (-23.5 to -5.9)	0.4
Bolivia	★☆☆☆☆	700 (524 to 894)	9.3 (5.9 to 12.6)	5.2 (3.6 to 7.2)	7.2 (5.5 to 9.1)	-12.5 (-35.1 to 23.4)	0.6	33000 (24500 to 43000)	396.4 (246.7 to 546.8)	221 (150.1 to 307.3)	308.3 (228.3 to 399.2)	-16.9 (-39.3 to -16.3)	0.6
Ecuador	★★★★☆	1270 (916 to 1480)	11.9 (7.2 to 14.6)	4 (3.5 to 4.7)	7.9 (5.6 to 9.2)	31.2 (-8.1 to 61.1)	0.3	64400 (47200 to 74900)	542 (332.6 to 671)	215.9 (184.5 to 253.1)	377.7 (276 to 440.5)	34.2 (-6.8 to 30.1)	0.4
Peru	★★★☆☆	917 (707 to 1180)	4.6 (3.2 to 6.2)	1.5 (1.1 to 2)	3 (2.3 to 3.9)	-30.4 (-48.5 to -8.7)	0.3	42100 (32400 to 54000)	191.5 (135 to 259.6)	68.5 (49.5 to 91)	129.5 (99.8 to 166.6)	-30.7 (-48.1 to -30.3)	0.4
Central Latin America		16200 (13100 to 19300)	11 (8.5 to 13.4)	2.1 (1.9 to 2.3)	6.4 (5.2 to 7.6)	14.6 (-5.9 to 31.3)	0.2	813000 (657000 to 988000)	503.8 (382.8 to 630.8)	106.2 (98.4 to 117.2)	302.4 (245.3 to 365.1)	19.7 (-4.3 to 18.8)	0.2
Colombia	★★★★☆	2820 (2200 to 3580)	9.9 (7.1 to 12.9)	1.8 (1.5 to 2.2)	5.7 (4.4 to 7.2)	-1.8 (-37.1 to 23.5)	0.2	138000 (107000 to 181000)	442.9 (323.4 to 601.7)	98 (80.6 to 118.6)	267.4 (206.9 to 350.2)	0.9 (-37.6 to -2.4)	0.2
Costa Rica	★★★★★	348 (250 to 413)	11.7 (8 to 14.2)	1.9 (1.6 to 2.2)	6.8 (4.9 to 8)	10.7 (-7.7 to 30)	0.2	16300 (11600 to 19700)	529.9 (346.4 to 658.8)	96.4 (82.1 to 113.1)	313.6 (224.1 to 378.9)	13.5 (-4.8 to 13.9)	0.2
El Salvador	★★★☆☆	591 (484 to 751)	16.5 (12.8 to 21.5)	3.8 (3 to 4.6)	9.4 (7.7 to 11.8)	-30.9 (-44.1 to -10.7)	0.2	30200 (24700 to 39300)	763.8 (592.4 to 1034.7)	207.4 (166.8 to 255.4)	458 (375.6 to 588.5)	-34.8 (-46.9 to -33.8)	0.3
Guatemala	★★★★☆	1060 (720 to 1690)	12.4 (7.9 to 20.8)	2.5 (1.6 to 3.6)	7.1 (4.9 to 11.1)	-22 (-50.1 to 35.8)	0.2	55400 (37500 to 91300)	560.5 (348.8 to 967.4)	129.8 (83.7 to 185.9)	333.8 (227.4 to 532.7)	-21.7 (-48.1 to -18.6)	0.2
Honduras	★★☆☆☆	307 (201 to 463)	7.1 (3.9 to 11.8)	2.3 (1.4 to 3.3)	4.6 (3 to 6.8)	2.2 (-33 to 61.3)	0.3	13800 (9100 to 21000)	268.1 (148.7 to 450.1)	83.3 (46.3 to 129.2)	174.6 (115.2 to 265.5)	-8.7 (-39.6 to -6)	0.3
Mexico	★★★★☆	7440 (5460 to 8330)	9.8 (6.7 to 11.3)	2 (1.9 to 2.2)	5.8 (4.3 to 6.5)	35.6 (1.1 to 51.2)	0.2	370000 (272000 to 417000)	446 (301.3 to 516.5)	104.1 (97.1 to 115)	273.5 (200.4 to 308.4)	49.7 (3 to 42.7)	0.2
Nicaragua	★★★☆☆	450 (353 to 560)	12.3 (8.9 to 16.1)	2.9 (2.2 to 3.7)	7.3 (5.7 to 9.1)	15.4 (-12 to 47.8)	0.2	23700 (18400 to 29500)	566.8 (411.1 to 738.8)	158.9 (118.9 to 207.6)	356.3 (279.2 to 444.2)	11.2 (-14.3 to 11.6)	0.3
Panama	★★★★☆	233 (177 to 313)	10.4 (7.5 to 14.3)	1.5 (1.2 to 1.9)	5.9 (4.5 to 7.9)	-2.2 (-25.5 to 30.7)	0.1	11200 (8340 to 15400)	473 (340.8 to 668.7)	73.2 (57 to 93.9)	273.5 (205.2 to 376)	0.7 (-24.9 to 1.8)	0.2
Venezuela	★★★★☆	2970 (2030 to 4600)	17.1 (11.2 to 27)	2.1 (1.6 to 2.7)	9.4 (6.5 to 14.3)	30.9 (-9.6 to 112.1)	0.1	154000 (101000 to 249000)	829.9 (517.7 to 1376.7)	98.4 (73.4 to 129)	463 (306.7 to 740.5)	33.5 (-2.2 to 48.4)	0.1
Tropical Latin America		14300 (11900 to 17600)	10.8 (8.8 to 14)	2.3 (2 to 2.6)	6.4 (5.3 to 7.9)	-11.6 (-22.4 to 4.4)	0.2	649000 (543000 to 803000)	464.7 (373.9 to 603.1)	103 (90.4 to 117.1)	280.9 (235.1 to 348.2)	-8.6 (-18.7 to -6.8)	0.2
Brazil	★★★★☆	13900 (11500 to 17200)	10.9 (8.7 to 14.2)	2.3 (2 to 2.6)	6.4 (5.3 to 7.9)	-12.8 (-23.7 to 3.7)	0.2	627000 (521000 to 786000)	465.6 (372.3 to 611.8)	100.6 (87.7 to 114.8)	279.9 (232 to 351.3)	-10 (-20.2 to -8.1)	0.2

Paraguay	★★★☆☆	422 (300 to 514)	9.7 (6 to 12.5)	3.4 (2.7 to 4.1)	6.5 (4.7 to 7.9)	70.4 (23.2 to 117.1)	0.4	21300 (14600 to 26300)	429.6 (241.3 to 557.3)	169.6 (135.7 to 212.2)	301.4 (211.2 to 369.6)	74.2 (24.6 to 73.7)	0.4
North Africa and Middle East		25900 (23000 to 30600)	6.8 (5.9 to 8.4)	2.6 (2.4 to 3)	4.8 (4.2 to 5.6)	-2.7 (-15.7 to 14.3)	0.4	1280000 (1140000 to 1530000)	308.9 (267 to 382.8)	117.9 (104.5 to 132.5)	216.3 (192.2 to 257.4)	-3.2 (-16.4 to -2.9)	0.4
North Africa and Middle East		25900 (22900 to 30500)	6.8 (5.9 to 8.4)	2.6 (2.4 to 3)	4.8 (4.2 to 5.6)	-2.8 (-15.7 to 14.3)	0.4	1280000 (1140000 to 1530000)	308.6 (266.7 to 382.3)	117.8 (104.4 to 132.3)	216 (192 to 257.1)	-3.2 (-16.4 to -2.9)	0.4
Afghanistan	★☆☆☆☆	2250 (1790 to 3680)	16.4 (13.2 to 26.3)	3.6 (2.9 to 4.5)	10.1 (8.4 to 15.2)	9.7 (-10.3 to 37.2)	0.2	116000 (90300 to 192000)	667.1 (514.3 to 1185.6)	140 (106.8 to 184.6)	413 (332 to 675.7)	7.2 (-14.7 to 7.5)	0.2
Algeria	★☆☆☆☆	1440 (1140 to 1960)	5.5 (4.1 to 8.1)	1.9 (1.5 to 2.4)	3.7 (3 to 5)	-10.4 (-33.7 to 24.4)	0.3	67800 (53600 to 93000)	239.6 (180.7 to 351.8)	82.9 (66.2 to 104.1)	161.4 (128.1 to 221.4)	-15.2 (-36.4 to -13.9)	0.3
Bahrain	★★★☆☆	96 (70 to 137)	9.7 (6.8 to 13.7)	2.8 (2 to 3.7)	7 (5.2 to 9.5)	-14.2 (-39.6 to 17.3)	0.3	4770 (3450 to 6830)	384.7 (265.1 to 571.3)	117.2 (82.6 to 158.9)	288 (211.6 to 406.4)	-15.9 (-40.4 to -15.5)	0.3
Egypt	★★★☆☆	3090 (2180 to 4290)	6.1 (3.9 to 9)	1.6 (1.1 to 2.1)	3.8 (2.7 to 5.3)	0 (-21.6 to 27.9)	0.3	144000 (102000 to 199000)	256 (168.6 to 374.3)	67.1 (44.9 to 93.7)	161.8 (113.3 to 223.6)	-6.4 (-27.1 to -6.2)	0.3
Iran	★★★☆☆	5010 (4070 to 6250)	7.9 (5.9 to 10.5)	3.9 (2.9 to 5.1)	5.9 (4.8 to 7.4)	-8.5 (-32.3 to 25.8)	0.5	256000 (206000 to 316000)	373.4 (269.1 to 498.7)	195.3 (146 to 256.8)	284.2 (230.3 to 353.4)	-9.7 (-33.8 to -10)	0.5
Iraq	★☆☆☆☆	2920 (2050 to 3870)	12.8 (8.1 to 18)	6.6 (4.4 to 9.8)	9.7 (7 to 12.7)	16 (-20.8 to 62)	0.5	149000 (104000 to 198000)	598.7 (367.1 to 854.5)	286.7 (187.5 to 430.6)	444.3 (312 to 589.6)	15.1 (-21.5 to 15.5)	0.5
Jordan	★★☆☆☆	218 (158 to 305)	4.9 (3.3 to 7)	2.1 (1.4 to 2.9)	3.5 (2.6 to 4.7)	-34.9 (-55 to -5.8)	0.4	10600 (7600 to 15000)	204.4 (132.6 to 307)	78.2 (53.3 to 112.8)	142.8 (103.2 to 199.7)	-37.3 (-56.8 to -36.6)	0.4
Kuwait	★★★★☆	113 (70.1 to 163)	3.8 (2.1 to 5.7)	1.3 (0.9 to 2)	2.7 (1.7 to 3.8)	25.7 (-21 to 85.2)	0.3	5670 (3520 to 8170)	165.6 (90.5 to 254.2)	53.3 (33 to 80.7)	116.8 (72.8 to 166.7)	23.1 (-22.8 to 23.1)	0.3
Lebanon	★☆☆☆☆	146 (99.1 to 218)	3.4 (2 to 5.6)	1.4 (1 to 1.9)	2.4 (1.6 to 3.5)	-44.2 (-61.3 to -8.7)	0.4	6830 (4680 to 10400)	149.6 (87.9 to 245.9)	60.2 (42.6 to 84.3)	104.6 (71.2 to 157.8)	-49.6 (-63.8 to -47.1)	0.4
Libya	★☆☆☆☆	282 (198 to 406)	7.5 (4.8 to 11.3)	2.1 (1.6 to 2.9)	4.7 (3.4 to 6.8)	13.7 (-17.2 to 55.4)	0.3	13400 (9350 to 19400)	321.6 (205.5 to 492.3)	89 (67 to 128.5)	204.5 (144.1 to 295.2)	9 (-19.7 to 11)	0.3
Morocco	★☆☆☆☆	1790 (1450 to 2210)	5.5 (4.2 to 7.5)	5.2 (4.1 to 6.6)	5.4 (4.4 to 6.7)	3.7 (-16.7 to 44.5)	0.9	80500 (63800 to 101000)	249.9 (186.6 to 344.9)	202.1 (150.7 to 251)	225.5 (180 to 282)	-1.4 (-20.6 to 0.4)	0.8
Palestine	★★☆☆☆	97.8 (76 to 129)	4.1 (3 to 5.7)	1.3 (1 to 1.8)	2.7 (2.1 to 3.6)	-6.8 (-35.9 to 35.8)	0.3	5070 (3900 to 6750)	179 (130.9 to 244.4)	46.7 (35.4 to 64.4)	113.5 (88.4 to 149.6)	-16.1 (-41.6 to -15.1)	0.3
Oman	★★☆☆☆	150 (113 to 212)	4.2 (3.2 to 5.7)	1.1 (0.9 to 1.3)	3.1 (2.5 to 4.2)	-1.8 (-34.8 to 75.9)	0.3	7590 (5630 to 10900)	169.4 (125.2 to 239.5)	39.5 (30.9 to 50.7)	128.7 (97.6 to 177.2)	-11.1 (-39.6 to -5.7)	0.2
Qatar	★★☆☆☆	135 (92 to 195)	6.3 (4.1 to 9.2)	1.6 (1 to 2.5)	5 (3.4 to 7.2)	-32.1 (-56.5 to 7.3)	0.3	6860 (4710 to 9870)	258 (168.2 to 384.3)	72.8 (43.8 to 113.3)	211.5 (143.4 to 306.4)	-36.4 (-59.1 to -35.6)	0.3
Saudi Arabia	★★☆☆☆	929 (710 to 1130)	4.4 (3.2 to 5.5)	1.3 (1 to 1.6)	3.1 (2.4 to 3.7)	17.2 (-21.3 to 90)	0.3	44900 (34500 to 54800)	194 (140.2 to 240.1)	48.9 (37.9 to 62.3)	132.3 (102.1 to 161.1)	11.3 (-24.3 to 14.2)	0.3
Sudan	☆☆☆☆☆	1750 (1280 to 2170)	7.9 (5.8 to 10.7)	2.5 (1.8 to 3.4)	5.2 (4 to 6.5)	8.4 (-11.8 to 46.3)	0.3	91100 (65700 to 114000)	363 (255.9 to 496.1)	117 (75.1 to 166)	239.5 (175.4 to 298.8)	4.5 (-15.4 to 5.6)	0.3
Syria	★★★☆☆	377 (307 to 468)	4 (3.1 to 5.1)	1 (0.8 to 1.2)	2.5 (2 to 3)	-7.7 (-29.4 to 23.7)	0.3	18700 (15000 to 23600)	173.6 (133.1 to 226.7)	38.1 (30.4 to 47.5)	106.3 (85.8 to 132.6)	-9.1 (-32.8 to -8.4)	0.2
Tunisia	★☆☆☆☆	436 (313 to 583)	5.8 (4 to 8.2)	1.8 (1.2 to 2.5)	3.7 (2.7 to 5)	5 (-22.9 to 39.3)	0.3	19500 (14100 to 26400)	257.1 (173.1 to 365.8)	68.2 (46.1 to 95.2)	161 (116.7 to 217.1)	2.2 (-25 to 2.6)	0.3
Turkey	★★★☆☆	2750 (2140 to 3430)	5.6 (4.2 to 7.3)	1.4 (1 to 1.7)	3.4 (2.6 to 4.2)	-24.1 (-43.2 to -0.6)	0.3	133000 (102000 to 166000)	257.6 (187.7 to 335)	64.7 (48.8 to 84.3)	159.9 (123.7 to 199.3)	-18.2 (-40.5 to -18.3)	0.3
United Arab Emirates	★☆☆☆☆	445 (291 to 697)	4.6 (3 to 7.3)	1.6 (1.1 to 2.4)	3.9 (2.6 to 6)	3.3 (-37 to 74.9)	0.3	22100 (14300 to 34700)	204.4 (131.1 to 332.1)	73.4 (47.1 to 110.8)	170.9 (113.2 to 265.5)	-2.3 (-41 to -1.6)	0.4
Yemen	☆☆☆☆☆	1450 (1040 to 1860)	8.9 (6.5 to 12.3)	4.2 (2.8 to 6)	6.5 (4.9 to 8.3)	12.8 (-12.8 to 63.7)	0.5	77000 (54600 to 99900)	407.1 (282.3 to 565.6)	189.4 (118.4 to 278.7)	298.9 (213.4 to 380.9)	8.7 (-18 to 8.5)	0.5
South Asia		252000 (214000 to 273000)	18.2 (12.7 to 20.2)	12.6 (11.2 to 13.9)	15.4 (12.9 to 16.6)	-17.5 (-25.8 to -3.2)	0.7	12600000 (10900000 to 13700000)	782.9 (562.1 to 868.9)	626.6 (556.1 to 690.5)	704.8 (605.1 to 764.8)	-24 (-30.9 to -23)	0.8
South Asia		252000 (214000 to 273000)	18.2 (12.7 to 20.2)	12.6 (11.2 to 13.9)	15.4 (12.9 to 16.6)	-17.5 (-25.8 to -3.2)	0.7	12600000 (10900000 to 13700000)	782.9 (562.1 to 868.9)	626.6 (556.1 to 690.5)	704.8 (605.1 to 764.8)	-24 (-30.9 to -23)	0.8
Bangladesh	★★☆☆☆	11600 (9690 to 13700)	8.9 (6.8 to 10.7)	6.4 (5.1 to 8.2)	7.6 (6.3 to 8.9)	-39.1 (-50.8 to -24.3)	0.7	584000 (487000 to 711000)	339.3 (266.6 to 438.8)	326.1 (253.1 to 422.1)	332.3 (279.1 to 398.6)	-49.7 (-59.8 to -49.6)	1
Bhutan	☆☆☆☆☆	50.3 (37.5 to 66.4)	9.8 (6.9 to 13.4)	3.5 (2.5 to 4.8)	7 (5.4 to 8.9)	-27.2 (-47.6 to 8.9)	0.4	2330 (1710 to 3240)	396 (270 to 572)	139.3 (96.5 to 199)	280.2 (208.3 to 374.3)	-30.9 (-51.2 to -29.9)	0.4
India	★★☆☆☆	230000 (194000 to 250000)	21.2 (14.6 to 23.6)	14.7 (13.1 to 16.2)	17.9 (15 to 19.4)	-15.2 (-23.7 to -0.3)	0.7	11500000 (9880000 to 12500000)	917 (641.1 to 1021.7)	733.3 (649.9 to 810.7)	825.6 (702.6 to 897.8)	-21 (-28.4 to -19.9)	0.8
Nepal	★☆☆☆☆	2970 (2270 to 3540)	14.2 (9 to 18)	8.5 (7 to 10.3)	11.1 (8.5 to 13.1)	-20.7 (-40.4 to 16.4)	0.6	152000 (117000 to 183000)	630.1 (394.1 to 803.3)	409.3 (328.6 to 503.9)	509.7 (388.1 to 611.8)	-27.8 (-46.9 to -27.4)	0.6
Pakistan	★☆☆☆☆	7040 (5650 to 8720)	3.9 (2.8 to 5.2)	3.7 (2.8 to 4.9)	3.8 (3.1 to 4.7)	0.7 (-24.1 to 34.2)	0.9	388000 (309000 to 483000)	186.7 (133.1 to 251.7)	194.4 (140.7 to 256.8)	190.2 (152.4 to 235.8)	0.1 (-26.1 to 0.6)	1
Sub-Saharan Africa		60900 (53700 to 70600)	16.8 (14.4 to 19.7)	6.8 (5.7 to 8)	11.5 (10.1 to 13.1)	-7.6 (-18.4 to 5.9)	0.4	2540000 (2240000 to 3e+06)	536.8 (457.9 to 654)	199.9 (168.4 to 234.8)	364.1 (321.5 to 422.7)	-12.1 (-23.3 to -11.4)	0.4
Central Sub-Saharan Africa		6630 (5080 to 8700)	16.6 (11.6 to 22.5)	5.9 (5 to 7.1)	10.9 (8.5 to 14)	-2.3 (-21.4 to 17.4)	0.4	275000 (208000 to 374000)	532.4 (370.4 to 747.5)	180.2 (150.9 to 220.7)	351.3 (269 to 462)	-3.5 (-24.1 to -4.1)	0.3
Angola	★☆☆☆☆	1230 (896 to 1740)	15.3 (11.3 to 21.2)	6.6 (4.6 to 9.2)	10.6 (8 to 14.5)	-11.6 (-36.6 to 37)	0.4	51200 (36300 to 75100)	457.3 (328.8 to 683.8)	202 (139 to 289.7)	324.2 (236.1 to 456.6)	-17 (-41.8 to -15.7)	0.4
Central African Republic	☆☆☆☆☆	462 (276 to 671)	23.8 (12.7 to 36.1)	6.9 (4.4 to 10.2)	14.8 (9 to 21)	5.9 (-20.9 to 41.4)	0.3	19200 (11300 to 28400)	801.8 (425.8 to 1250.5)	222.8 (137.8 to 341)	502.2 (295.8 to 730.4)	6.8 (-20.9 to 7.9)	0.3
Congo	☆☆☆☆☆	292 (198 to 430)	14.9 (9 to 23.6)	7 (4.8 to 10)	10.8 (7.4 to 15.5)	-25.7 (-50.2 to 12.8)	0.5	11600 (7700 to 18000)	473.6 (274.3 to 771.5)	222.3 (148.2 to 329.6)	345.6 (232.3 to 515.7)	-28 (-52.5 to -27.1)	0.5
Democratic Republic of the Congo	★☆☆☆☆	4460 (3190 to 6020)	16.7 (10.5 to 23.5)	5.6 (4.5 to 7.1)	10.8 (7.8 to 14)	4.7 (-15.7 to 25.8)	0.3	187000 (131000 to 257000)	542.6 (343.6 to 779.6)	168.4 (135.5 to 211.1)	350.5 (248.4 to 473)	3 (-18.9 to 3)	0.3
Equatorial Guinea	☆☆☆☆☆	48.9 (27 to 79.9)	14.1 (7.4 to 22.7)	4.8 (2.4 to 8.9)	9.5 (5.4 to 15)	-39.6 (-67.4 to 19)	0.3	1890 (1040 to 3150)	416.8 (210.7 to 718)	148.6 (69.4 to 277.6)	287.9 (160 to 476.2)	-47.9 (-71.8 to -46.1)	0.4
Gabon	☆☆☆☆☆	127 (88.1 to 182)	16.5 (9.9 to 25.5)	5.6 (4 to 7.6)	10.8 (7.6 to 15.2)	-16.8 (-44.7 to 25.7)	0.3	4760 (3230 to 6970)	516.1 (305 to 828.6)	170.2 (117.5 to 242.4)	343.3 (235.9 to 504.9)	-18.8 (-46.9 to -18)	0.3
Eastern Sub-Saharan Africa		24200 (21500 to 27500)	18.7 (15.8 to 22.1)	7 (5.9 to 8.2)	12.5 (11.1 to 14.1)	-15.3 (-26.5 to 2.3)	0.4	969000 (848000 to 1110000)	555.7 (464.5 to 665.8)	203.8 (172.4 to 236.9)	373.5 (330.5 to 425.2)	-18.1 (-29.1 to -16.9)	0.4
Burundi	★☆☆☆☆	821 (636 to 1030)	21.5 (15.6 to 29.7)	8.5 (6.1 to 11.5)	14.6 (11.4 to 18.3)	-33.8 (-50.2 to -8.2)	0.4	33800 (26000 to 43000)	647 (461 to 900.1)	260.1 (182.2 to 359)	447.4 (345.9 to 565.3)	-35.4 (-51.6 to -34.9)	0.4
Comoros	☆☆☆☆☆	39 (24 to 76.5)	13.3 (7.3 to 29.9)	4.8 (2.2 to 7.4)	8.7 (5.4 to 17.1)	-0.5 (-27.5 to 33.6)	0.4	1630 (984 to 3160)	399.5 (219.1 to 907.6)	153.2 (69.5 to 239.9)	273.4 (167.5 to 536.5)	-6.3 (-31 to -3)	0.4
Djibouti	☆☆☆☆☆	40.1 (23.4 to 71)	10.2 (5.6 to 19.9)	3.6 (1.6 to 6.2)	6.7 (4 to 11.8)	5.6 (-28.3 to 50.8)	0.4	1570 (907 to 2780)	309.9 (166.1 to 613.7)	107.2 (43.9 to 186.6)	207.1 (120.8 to 366)	1.7 (-30.6 to 4.3)	0.3
Eritrea	☆☆☆☆☆	353 (286 to 431)	20.1 (15.6 to 25.5)	7.5 (5.9 to 9.3)	12.9 (10.6 to 15.5)	-20.9 (-40.3 to 5.6)	0.4	15100 (12000 to 18600)	613.9 (470.2 to 781.3)	225.5 (174.1 to 284)	408.3 (331.5 to 497.2)	-21.5 (-42.5 to -21.8)	0.4
Ethiopia	★★☆☆☆	6980 (5540 to 8620)	19.6 (14.6 to 25.1)	6.5 (4.9 to 8.4)	12.7 (10.1 to 15.5)	-29.4 (-47 to 3.8)	0.3	279000 (219000 to 344000)	583.7 (426.4 to 760.6)	201.4 (149.5 to 263.3)	386.8 (304.9 to 475)	-33.5 (-50.4 to -32.7)	0.3
Kenya	★☆☆☆☆	2110 (1660 to 2650)	15.4 (11.6 to 19.4)	3.5 (2.5 to 4.6)	9 (7.1 to 11.1)	5.7 (-17.4 to 48.6)	0.2	83700 (65000 to 107000)	435.8 (328.9 to 567.1)	100.6 (71.5 to 133.1)	263 (207.4 to 332.1)	9.9 (-15.6 to 7.8)	0.2
Madagascar	★☆☆☆☆	1430 (1110 to 1800)	16.2 (12 to 21.4)	5.5 (3.8 to 7.5)	10.6 (8.3 to 13.1)	-12.5 (-32.8 to 16.7)	0.3	59300 (45000 to 76000)	500.2 (353.8 to 673.7)	173.7 (118.9 to 235.3)	332.7 (256.9 to 419.1)	-13.8 (-35.7 to -13)	0.3
Malawi	★☆☆☆☆	1070 (821 to 1370)	20.4 (15.4 to 26.9)	5.7 (3.8 to 7.6)	12.4 (9.8 to 15.6)	10.5 (-20.3 to 54.3)	0.3	42700 (31600 to 55200)	597.7 (431.6 to 795.9)	164.6 (108.6 to 222.4)	373.1 (282.7 to 475.8)	10.6 (-23.5 to 9.3)	0.3
Mozambique	★☆☆☆☆	1870 (1430 to 2340)	22 (16.5 to 28.1)	5.2 (3.8 to 6.9)	12.5 (9.9 to 15.5)	-9.2 (-28.4 to 18.1)	0.2	75800 (56600 to 95900)	666.3 (472.7 to 873.5)	156.8 (117.3 to 204.9)	390.1 (297.5 to 490.6)	-11.6 (-32.4 to -11.1)	0.2
Rwanda	★☆☆☆☆	698 (543 to 901)	17.9 (13.3 to 24.6)	6.9 (4.9 to 9.1)	11.5 (9 to 14.5)	-39.8 (-55.2 to -12)	0.4	28100 (21600 to 36900)	508.6 (365 to 725.1)	202.5 (144.6 to 273.8)	336.6 (260.9 to 434.4)	-43.2 (-57.5 to -41.2)	0.4
Somalia	☆☆☆☆☆	512 (338 to 774)	11.8 (7.3 to 21.4)	7.6 (4.6 to 11.6)	9.5 (6.3 to 14.5)	-2.9 (-23.6 to 24.8)	0.6	20800 (13800 to 31600)	360.6 (223.8 to 659.6)	234.7 (140.9 to 368.5)	295.7 (193.9 to 448.5)	-4.3 (-26.2 to -3.4)	0.7
South Sudan	☆☆☆☆☆	455 (264 to 766)	9 (5.2 to 17.6)	4.8 (2.5 to 7.3)	6.8 (4 to 11.3)	7.9 (-19 to 43)	0.5	17700 (10100 to 30400)	265.8 (146 to 531)	142.4 (74.1 to 220.7)	202.6 (116.2 to 341.7)	5.7 (-21.5 to 6.6)	0.5
Tanzania	★☆☆☆☆	3120 (2550 to 3900)	17.3 (13.5 to 22.3)	5.9 (4.3 to 7.7)	11.4 (9.3 to 14)	-4.1 (-24.7 to 24.4)	0.3	124000 (99100 to 156000)	516.2 (395.7 to 677)	173.9 (124.7 to 231.8)	341 (277.8 to 428.5)	-8.6 (-27.7 to -7.4)	0.3
Uganda	★☆☆☆☆	3320 (2680 to 4030)	22.4 (17.3 to 28.1)	18.7 (12.7 to 25.5)	20.6 (16 to 25.1)	-16.1 (-33.1 to 12.3)	0.8	130000 (105000 to 159000)	675.3 (527.6 to 852.2)	471.3 (328.8 to 647.4)	574.9 (463 to 703.3)	-14.9 (-32.5 to -14.1)	0.7
Zambia	★☆☆☆☆	1380 (1050 to 1800)	28.4 (19.7 to 37.1)	8.6 (4.9 to 12.4)	17.7 (13.4 to 22.8)	61.6 (5.2 to 128.7)	0.3	56200 (42500 to 73600)	851.4 (585.9 to 1156.8)	261.9 (149.1 to 393.1)	545 (410.2 to 713.8)	66.2 (5.9 to 63)	0.3
Southern Sub-Saharan Africa		10900 (9570 to 13200)	26.1 (21.9 to 31.6)	7.8 (6.7 to 8.9)	16.3 (14.3 to 19.3)	-10.8 (-25 to 19.1)	0.3	510000 (442000 to 628000)	1056.5 (896.2 to 1341.8)	296.1 (250.8 to 344.3)	664.1 (579.6 to 809.8)	-17.2 (-30.1 to -16.2)	0.3
Botswana	☆☆☆☆☆	346 (152 to 563)	29.1 (13.5 to 48.2)	6.9 (1.6 to 13.5)	17.6 (8 to 27.6)	10.1 (-46.5 to 85.3)	0.2	16500 (7050 to 27100)	1202.1 (522.8 to 1986.4)	256.1 (54.6 to 540.9)	726.2 (319.1 to 1177.8)	7.9 (-47.8 to 8.5)	0.2
Lesotho	☆☆☆☆☆	566 (384 to 817)	40.2 (25.6 to 60.1)	35.3 (16.8 to 54.7)	39 (25.5 to 55.7)	37.8 (-8.5 to 104.2)	0.9	25300 (17300 to 36800)	1692.3 (1081.2 to 2534)	1084.6 (488.6 to 1787.7)	1413.2 (944.9 to 2065.9)	43.8 (-6.1 to 45.4)	0.6
Namibia	☆☆☆☆☆	255 (174 to 360)	24 (17 to 34.9)	3.8 (1.4 to 6.5)	13 (9 to 18)	-18.9 (-44.2 to 13)	0.2	12400 (8360 to 17800)	980.6 (675.6 to 1465.1)	137.4 (46.8 to 258.7)	537.1 (366.1 to 762.6)	-18.3 (-44.9 to -18.7)	0.1
South Africa	★★☆☆☆	7370 (6130 to 8380)	22.7 (18.6 to 26.2)	6.6 (5.6 to 7.6)	14.2 (12 to 16.1)	-26.3 (-38.4 to -4.2)	0.3	356000 (291000 to 410000)	977.9 (771.1 to 1140.6)	292.7 (240.7 to 343.5)	626.7 (516.7 to 717.1)	-28.9 (-41.5 to -28.7)	0.3
Swaziland	★☆☆☆☆	185 (117 to 271)	31.4 (20.1 to 46)	7.3 (3 to 13.5)	18.7 (11.7 to 27)	22.2 (-16.1 to 72.7)	0.2	9250 (5950 to 13400)	1320.9 (848.7 to 1920.9)	271.1 (101.3 to 535.1)	783 (491 to 1161.3)	27.7 (-12.9 to 28.9)	0.2
Zimbabwe	★★☆☆☆	2200 (1630 to 3570)	43.2 (30.4 to 59.8)	14 (9.7 to 18.7)	27.8 (21.1 to 37.2)	96.2 (30.7 to 268.4)	0.3	90700 (64500 to 172000)	1383.3 (937.6 to 2360.2)	334.1 (220.3 to 464.1)	839.9 (619.5 to 1335.2)	87.7 (23.1 to 90.6)	0.2
Western Sub-Saharan Africa		19100 (15800 to 24900)	12.6 (10.2 to 17.1)	6.7 (4.8 to 8.9)	9.6 (7.9 to 12.3)	4.3 (-10.4 to 20.7)	0.5	783000 (644000 to 1020000)	397.1 (316.9 to 543.2)	182.2 (133.9 to 240.8)	288.9 (238.1 to 375.7)	3.8 (-10.5 to 4.4)	0.5
Benin	★☆☆☆☆	847 (672 to 1020)	20.4 (15.2 to 25.4)	8.2 (6.2 to 10.4)	13.9 (11.1 to 16.4)	23.9 (-0.5 to 52.8)	0.4	35500 (28000 to 43700)	672.6 (495.3 to 848.7)	228.1 (175.5 to 286.3)	444.8 (352.4 to 537.5)	24 (-3.2 to 23)	0.3
Burkina Faso	★☆☆☆☆	1170 (998 to 1420)	19.5 (15.7 to 23.8)	7.9 (6.5 to 9.5)	12.9 (11.1 to 15.1)	-4.4 (-19.8 to 17.5)	0.4	50200 (42200 to 62900)	595.7 (485 to 762.5)	246.3 (202.3 to 298.6)	408.6 (350.2 to 492.6)	-2.9 (-18.6 to -2.4)	0.4
Cameroon	☆☆☆☆☆	2180 (1640 to 2800)	23.6 (16.8 to 29.9)	10.7 (7.1 to 15)	16.9 (13 to 21.2)	37.6 (4.9 to 76.8)	0.5	90300 (66200 to 117000)	780.8 (540.6 to 1017.2)	297.4 (192.4 to 420.8)	536.4 (399.3 to 691.6)	38.1 (2.5 to 37.7)	0.4
Cape Verde	★★☆☆☆	86.9 (65 to 107)	36.2 (26.9 to 45.7)	6.4 (5 to 7.9)	20.1 (15.6 to 24.5)	33.3 (0 to 76.6)	0.2	3760 (2600 to 4750)	1349.8 (935.2 to 1724.1)	183 (143.1 to 231.5)	745.5 (543.2 to 927.2)	34.3 (-1 to 34.4)	0.1
Chad	☆☆☆☆☆	642 (448 to 929)	11.1 (7.6 to 18)	8.9 (5.2 to 13)	10 (6.9 to 14.5)	14.5 (-7.6 to 42.4)	0.8	26400 (18600 to 38400)	365.9 (249.9 to 588.1)	234.3 (139.8 to 333.3)	300.7 (208.1 to 436.6)	12.1 (-8.1 to 13.8)	0.6
Cote d'Ivoire	★☆☆☆☆	2050 (1690 to 2510)	24.4 (19.8 to 29.8)	7.9 (6 to 10.5)	16.6 (13.8 to 19.9)	17.6 (-5.8 to 49.1)	0.3	83600 (68300 to 105000)	778.1 (618.9 to 983.7)	235.1 (177.5 to 315.9)	517 (427.1 to 636.2)	15.6 (-8.7 to 15.6)	0.3
The Gambia	★☆☆☆☆	70 (50.4 to 109)	9.7 (6.2 to 17.6)	5.4 (4.1 to 6.9)	7.5 (5.5 to 11.5)	1.5 (-21 to 30.5)	0.6	2950 (2070 to 4730)	307.2 (192.3 to 565.5)	147.4 (112.2 to 185.7)	224.7 (162.6 to 350.1)	-1.8 (-22.8 to -0.2)	0.5
Ghana	★☆☆☆☆	1400 (1140 to 1700)	15.7 (12.3 to 19)	2.3 (1.8 to 2.8)	8.4 (6.8 to 10)	17.5 (-6.7 to 47.8)	0.1	57300 (46400 to 71100)	477.4 (373.5 to 595.7)	74.5 (58.4 to 94.2)	266.6 (217.3 to 324.8)	7.8 (-13.5 to 8.5)	0.2
Guinea	★☆☆☆☆	671 (457 to 988)	11.8 (7.6 to 19.5)	7.6 (4.8 to 10.6)	9.7 (6.6 to 14.1)	30.4 (1.1 to 61.8)	0.6	27100 (18700 to 40800)	384 (248.8 to 646.1)	212 (137.7 to 291.8)	297.9 (203.8 to 439.7)	30.5 (0.4 to 30.6)	0.6

Guinea-Bissau	★☆☆☆☆	137<br>(99.5 to 202)	16.3<br>(11.2 to 26.2)	9.1<br>(6.2 to 12.6)	12.5<br>(9.1 to 18.2)	16.4<br>(-8.8 to 51.9)	0.6	5720<br>(4100 to 8570)	558.8<br>(386.2 to 908.9)	266.4<br>(181.2 to 374.4)	410.4<br>(296.4 to 604)	12.7<br>(-11.5 to 13.5)	0.5
Liberia	★☆☆☆☆	381<br>(305 to 455)	15.8<br>(11.6 to 19.4)	17<br>(12.5 to 21.5)	16.5<br>(13.3 to 19.6)	45.9<br>(17 to 78.4)	1.1	14400<br>(11500 to 17300)	497.2<br>(362.4 to 621.2)	446.9<br>(331.4 to 562.9)	475.4<br>(378.9 to 569.7)	39.7<br>(11.3 to 39)	0.9
Mali	★☆☆☆☆	511<br>(331 to 874)	8.6<br>(5.1 to 17)	3.5<br>(2.2 to 5.2)	6<br>(3.9 to 10.1)	-5.6<br>(-29.5 to 37.8)	0.4	22100<br>(14400 to 37700)	269.5<br>(154.8 to 537.1)	106.6<br>(66.5 to 155.4)	186.5<br>(120.2 to 320.7)	-13.4<br>(-34.4 to -9.7)	0.4
Mauritania	☆☆☆☆☆	133<br>(73.7 to 265)	8.3<br>(4.2 to 18.4)	3.3<br>(1.8 to 5.1)	5.6<br>(3.3 to 10.6)	-11.9<br>(-40.3 to 26.9)	0.4	5440<br>(2820 to 11300)	255<br>(112.2 to 593.8)	96.8<br>(50.3 to 154.8)	174.1<br>(95.6 to 350.4)	-21.4<br>(-47.8 to -16.3)	0.4
Niger	★☆☆☆☆	752<br>(496 to 1230)	10.2<br>(6.3 to 19)	5.8<br>(3.3 to 8.8)	7.9<br>(5.2 to 12.9)	14.4<br>(-12.5 to 50.7)	0.6	30700<br>(20100 to 50700)	330.9<br>(198.2 to 616.4)	162.2<br>(91.2 to 243.8)	245.7<br>(161.2 to 404.8)	7.7<br>(-16.6 to 10.6)	0.5
Nigeria	★☆☆☆☆	6090<br>(3830 to 10300)	7.4<br>(3.8 to 14.6)	6.5<br>(3.2 to 11)	7<br>(4.4 to 11.5)	-15.9<br>(-38.3 to 11.5)	0.9	244000<br>(153000 to 420000)	232.3<br>(113.6 to 459.9)	162.5<br>(79.3 to 275.5)	198.5<br>(124.2 to 334.9)	-17<br>(-39.4 to -15.2)	0.7
Sao Tome and Principe	★☆☆☆☆	3.76<br>(2.56 to 5.16)	4.3<br>(2.6 to 6.3)	1.8<br>(1.3 to 2.4)	3<br>(2.2 to 4)	12.8<br>(-18.5 to 52.3)	0.4	176<br>(113 to 247)	165.1<br>(90.6 to 245.9)	60.8<br>(39.9 to 84.6)	111.9<br>(76.2 to 153.5)	9.3<br>(-23.5 to 9.5)	0.4
Senegal	★☆☆☆☆	1040<br>(882 to 1280)	21.8<br>(17.3 to 26.6)	7<br>(5.7 to 8.8)	13.6<br>(11.4 to 16)	17.9<br>(-2.3 to 44)	0.3	41700<br>(34800 to 54200)	647.1<br>(521.4 to 832.1)	203.5<br>(163.4 to 255.3)	408<br>(345.4 to 501)	15.4<br>(-5 to 15.8)	0.3
Sierra Leone	★☆☆☆☆	334<br>(248 to 473)	11.4<br>(7.8 to 18.3)	8.2<br>(5.7 to 11.4)	9.8<br>(7.2 to 13.7)	39.6<br>(10.5 to 71)	0.7	14100<br>(10400 to 20200)	377.7<br>(253.7 to 614.1)	234.7<br>(168 to 324)	305.1<br>(225.2 to 431.3)	35.6<br>(6.1 to 35.6)	0.6
Togo	☆☆☆☆☆	645<br>(502 to 797)	23.1<br>(16.8 to 29.2)	8.7<br>(6.6 to 11)	15.5<br>(12.3 to 18.9)	29.4<br>(0.4 to 64.3)	0.4	28000<br>(21500 to 35100)	792.7<br>(561.4 to 1012)	247.8<br>(189.5 to 314.7)	511<br>(398.3 to 629.8)	31.4<br>(0 to 30.9)	0.3

SR Table 2. Number of location years of data by source type for fatal self-harm (suicide) data		
Location	Data Source Type	
	Vital registration	Other
China	106562	0
North Korea	0	0
Taiwan	1472	0
Cambodia	0	79
Indonesia	0	1752
Laos	0	0
Malaysia	1106	0
Maldives	169	0
Mauritius	3320	0
Myanmar	0	17
Philippines	2440	0
Sri Lanka	1000	0
Seychelles	749	0
Thailand	600	120
Timor-Leste	0	0
Vietnam	0	128
American Samoa	1686	0
Federated States of Micronesia	0	0
Fiji	1240	0
Guam	2253	0
Kiribati	682	0
Marshall Islands	0	0
Northern Mariana Islands	1575	0
Papua New Guinea	0	0
Samoa	0	0
Solomon Islands	0	0
Tonga	40	0
Vanuatu	0	0
Armenia	1480	0
Azerbaijan	1000	0
Georgia	1880	0
Kazakhstan	1520	0
Kyrgyzstan	2582	0
Mongolia	4	0
Tajikistan	920	0
Turkmenistan	1240	0
Uzbekistan	1800	0
Albania	2139	0
Bosnia and Herzegovina	476	0
Bulgaria	3280	0
Croatia	3600	0
Czech Republic	3588	0

Hungary	4317	0
Macedonia	2600	0
Montenegro	840	0
Poland	2495	0
Romania	2800	0
Serbia	2160	0
Slovakia	2640	0
Slovenia	3408	0
Belarus	1080	0
Estonia	2960	0
Latvia	2920	0
Lithuania	3080	0
Moldova	2920	0
Russia	0	0
Ukraine	1240	0
Brunei	1079	0
Japan	191535	0
South Korea	2680	0
Singapore	2786	0
Australia	4200	0
New Zealand	4200	0
Andorra	0	0
Austria	4120	0
Belgium	3800	0
Cyprus	1317	0
Denmark	3080	0
Finland	2920	0
France	4080	0
Germany	3000	0
Greece	3160	0
Iceland	3652	0
Ireland	4000	0
Israel	2976	0
Italy	3720	0
Luxembourg	4039	0
Malta	4170	0
Netherlands	4320	0
Norway	3720	0
Portugal	3960	0
Spain	4200	0
Sweden	8637	0
Switzerland	2880	0
United Kingdom	625110	0
Argentina	2840	0
Chile	2800	0
Uruguay	2760	0
Canada	3480	0

Greenland	2244	0
United States	217004	0
Antigua and Barbuda	1991	0
The Bahamas	2237	0
Barbados	2305	0
Belize	2720	0
Bermuda	2630	0
Cuba	2772	0
Dominica	2591	0
Dominican Republic	2600	0
Grenada	2222	0
Guyana	2372	0
Haiti	0	0
Jamaica	1667	0
Puerto Rico	0	0
Saint Lucia	2679	0
Saint Vincent and the Grenadines	2462	0
Suriname	2760	0
Trinidad and Tobago	2200	0
Virgin Islands, U.S.	2263	0
Bolivia	0	0
Ecuador	2920	0
Peru	1640	0
Colombia	4080	0
Costa Rica	2840	0
El Salvador	2440	0
Guatemala	2480	0
Honduras	120	0
Mexico	138120	0
Nicaragua	2360	0
Panama	2520	0
Venezuela	2680	0
Brazil	100515	0
Paraguay	2840	0
Afghanistan	0	40
Algeria	0	0
Bahrain	1960	0
Egypt	8	0
Iran	800	0
Iraq	120	0
Jordan	960	0
Kuwait	2800	0
Lebanon	0	0
Libya	0	0
Morocco	0	0
Palestine	1360	0

Oman	40	0
Qatar	1560	0
Saudi Arabia	8536	0
Sudan	0	0
Syria	560	0
Tunisia	0	0
Turkey	600	47
United Arab Emirates	0	61
Yemen	0	0
Bangladesh	0	1793
Bhutan	0	40
India	81	912
Nepal	0	0
Pakistan	0	35
Angola	0	40
Central African Republic	0	0
Congo	0	0
Democratic Republic of the Congo	0	0
Equatorial Guinea	0	0
Gabon	0	0
Burundi	0	0
Comoros	0	0
Djibouti	0	0
Eritrea	0	0
Ethiopia	0	177
Kenya	0	1000
Madagascar	0	0
Malawi	0	376
Mozambique	0	120
Rwanda	0	0
Somalia	0	0
South Sudan	0	0
Tanzania	0	120
Uganda	0	0
Zambia	80	0
Botswana	0	0
Lesotho	0	0
Namibia	0	0
South Africa	18760	1300
Swaziland	0	0
Zimbabwe	110	0
Benin	0	0
Burkina Faso	0	167
Cameroon	0	0
Cape Verde	244	0
Chad	0	0

Cote d'Ivoire	0	20
The Gambia	0	20
Ghana	202	328
Guinea	0	0
Guinea-Bissau	0	0
Liberia	0	0
Mali	0	0
Mauritania	0	0
Niger	0	0
Nigeria	0	0
Sao Tome and Principe	40	0
Senegal	0	40
Sierra Leone	0	0
Togo	0	0

SR Table 3. CODEm covariates used in suicide death estimation and expected direction of covariate by sex, and age					
**Cause**	**Sex**	**Age start**	**Age end**	**Direction**	**Covariate**
Self-harm and interpersonal violence	Female	0-6 days	95+ years	1	Alcohol (liters per capita)
Self-harm and interpersonal violence	Female	0-6 days	95+ years	-1	Education (years per capita)
Self-harm and interpersonal violence	Female	0-6 days	95+ years	0	LDI (I$ per capita)
Self-harm and interpersonal violence	Female	0-6 days	95+ years	0	Elevation Over 1500m (proportion)
Self-harm and interpersonal violence	Female	0-6 days	95+ years	0	Population Density (over 1000 ppl/sqkm, proportion)
Self-harm and interpersonal violence	Female	0-6 days	95+ years	0	Population Density (under 150 ppl/sqkm, proportion)
Self-harm and interpersonal violence	Female	0-6 days	95+ years	0	Elevation Under 100m (proportion)
Self-harm and interpersonal violence	Female	0-6 days	95+ years	1	Log-transformed SEV scalar: Oth Unint
Self-harm and interpersonal violence	Female	0-6 days	95+ years	1	Healthcare access and quality index
Self-harm and interpersonal violence	Male	0-6 days	95+ years	1	Alcohol (liters per capita)
Self-harm and interpersonal violence	Male	0-6 days	95+ years	-1	Education (years per capita)
Self-harm and interpersonal violence	Male	0-6 days	95+ years	0	LDI (I$ per capita)
Self-harm and interpersonal violence	Male	0-6 days	95+ years	0	Elevation Over 1500m (proportion)
Self-harm and interpersonal violence	Male	0-6 days	95+ years	0	Population Density (over 1000 ppl/sqkm, proportion)
Self-harm and interpersonal violence	Male	0-6 days	95+ years	0	Population Density (under 150 ppl/sqkm, proportion)
Self-harm and interpersonal violence	Male	0-6 days	95+ years	0	Elevation Under 100m (proportion)
Self-harm and interpersonal violence	Male	0-6 days	95+ years	1	Log-transformed SEV scalar: Oth Unint
Self-harm and interpersonal violence	Male	0-6 days	95+ years	1	Healthcare access and quality index
Self-harm	Female	10-14 years	95+ years	1	Alcohol (liters per capita)
Self-harm	Female	10-14 years	95+ years	0	Education (years per capita)
Self-harm	Female	10-14 years	95+ years	0	LDI (I$ per capita)
Self-harm	Female	10-14 years	95+ years	0	Population Density (150-300 ppl/sqkm, proportion)
Self-harm	Female	10-14 years	95+ years	0	Population Density (300-500 ppl/sqkm, proportion)
Self-harm	Female	10-14 years	95+ years	0	Population Density (500-1000 ppl/sqkm, proportion)
Self-harm	Female	10-14 years	95+ years	0	Population Density (over 1000 ppl/sqkm, proportion)
Self-harm	Female	10-14 years	95+ years	0	Population Density (under 150 ppl/sqkm, proportion)

Self-harm	Female	10-14 years	95+ years	-1	Religion (binary, >50% Muslim)
Self-harm	Female	10-14 years	95+ years	1	Log-transformed SEV scalar: Self Harm
Self-harm	Female	10-14 years	95+ years	0	Socio-demographic Index
Self-harm	Female	10-14 years	95+ years	1	Major depressive disorder
Self-harm	Female	10-14 years	95+ years	-1	Healthcare access and quality index
Self-harm	Male	10-14 years	95+ years	1	Alcohol (liters per capita)
Self-harm	Male	10-14 years	95+ years	0	Education (years per capita)
Self-harm	Male	10-14 years	95+ years	0	LDI (I$ per capita)
Self-harm	Male	10-14 years	95+ years	0	Population Density (150-300 ppl/sqkm, proportion)
Self-harm	Male	10-14 years	95+ years	0	Population Density (300-500 ppl/sqkm, proportion)
Self-harm	Male	10-14 years	95+ years	0	Population Density (500-1000 ppl/sqkm, proportion)
Self-harm	Male	10-14 years	95+ years	0	Population Density (over 1000 ppl/sqkm, proportion)
Self-harm	Male	10-14 years	95+ years	0	Population Density (under 150 ppl/sqkm, proportion)
Self-harm	Male	10-14 years	95+ years	-1	Religion (binary, >50% Muslim)
Self-harm	Male	10-14 years	95+ years	0	Socio-demographic Index
Self-harm	Male	10-14 years	95+ years	-1	Healthcare access and quality index
Self-harm by firearm	Female	10-14 years	95+ years	1	Alcohol (liters per capita)
Self-harm by firearm	Female	10-14 years	95+ years	0	Education (years per capita)
Self-harm by firearm	Female	10-14 years	95+ years	0	LDI (I$ per capita)
Self-harm by firearm	Female	10-14 years	95+ years	0	Population Density (150-300 ppl/sqkm, proportion)
Self-harm by firearm	Female	10-14 years	95+ years	0	Population Density (300-500 ppl/sqkm, proportion)
Self-harm by firearm	Female	10-14 years	95+ years	0	Population Density (500-1000 ppl/sqkm, proportion)
Self-harm by firearm	Female	10-14 years	95+ years	0	Population Density (over 1000 ppl/sqkm, proportion)
Self-harm by firearm	Female	10-14 years	95+ years	0	Population Density (under 150 ppl/sqkm, proportion)
Self-harm by firearm	Female	10-14 years	95+ years	-1	Religion (binary, >50% Muslim)
Self-harm by firearm	Female	10-14 years	95+ years	1	Log-transformed SEV scalar: Self Harm
Self-harm by firearm	Female	10-14 years	95+ years	0	Socio-demographic Index
Self-harm by firearm	Female	10-14 years	95+ years	1	Major depressive disorder
Self-harm by firearm	Female	10-14 years	95+ years	-1	Healthcare access and quality index

Self-harm by firearm	Male	10-14 years	95+ years	1	Alcohol (liters per capita)
Self-harm by firearm	Male	10-14 years	95+ years	0	Education (years per capita)
Self-harm by firearm	Male	10-14 years	95+ years	0	LDI (I$ per capita)
Self-harm by firearm	Male	10-14 years	95+ years	0	Population Density (150-300 ppl/sqkm, proportion)
Self-harm by firearm	Male	10-14 years	95+ years	0	Population Density (300-500 ppl/sqkm, proportion)
Self-harm by firearm	Male	10-14 years	95+ years	0	Population Density (500-1000 ppl/sqkm, proportion)
Self-harm by firearm	Male	10-14 years	95+ years	0	Population Density (over 1000 ppl/sqkm, proportion)
Self-harm by firearm	Male	10-14 years	95+ years	0	Population Density (under 150 ppl/sqkm, proportion)
Self-harm by firearm	Male	10-14 years	95+ years	-1	Religion (binary, >50% Muslim)
Self-harm by firearm	Male	10-14 years	95+ years	1	Log-transformed SEV scalar: Self Harm
Self-harm by firearm	Male	10-14 years	95+ years	0	Socio-demographic Index
Self-harm by firearm	Male	10-14 years	95+ years	1	Major depressive disorder
Self-harm by firearm	Male	10-14 years	95+ years	-1	Healthcare access and quality index
Self-harm by other specified means	Female	10-14 years	95+ years	1	Alcohol (liters per capita)
Self-harm by other specified means	Female	10-14 years	95+ years	0	Education (years per capita)
Self-harm by other specified means	Female	10-14 years	95+ years	0	LDI (I$ per capita)
Self-harm by other specified means	Female	10-14 years	95+ years	0	Population Density (150-300 ppl/sqkm, proportion)
Self-harm by other specified means	Female	10-14 years	95+ years	0	Population Density (300-500 ppl/sqkm, proportion)
Self-harm by other specified means	Female	10-14 years	95+ years	0	Population Density (500-1000 ppl/sqkm, proportion)
Self-harm by other specified means	Female	10-14 years	95+ years	0	Population Density (over 1000 ppl/sqkm, proportion)
Self-harm by other specified means	Female	10-14 years	95+ years	0	Population Density (under 150 ppl/sqkm, proportion)
Self-harm by other specified means	Female	10-14 years	95+ years	-1	Religion (binary, >50% Muslim)
Self-harm by other specified means	Female	10-14 years	95+ years	1	Log-transformed SEV scalar: Self Harm
Self-harm by other specified means	Female	10-14 years	95+ years	0	Socio-demographic Index
Self-harm by other specified means	Female	10-14 years	95+ years	1	Major depressive disorder
Self-harm by other specified means	Female	10-14 years	95+ years	-1	Healthcare access and quality index
Self-harm by other specified means	Male	10-14 years	95+ years	1	Alcohol (liters per capita)
Self-harm by other specified means	Male	10-14 years	95+ years	0	Education (years per capita)
Self-harm by other specified means	Male	10-14 years	95+ years	0	LDI (I$ per capita)

Self-harm by other specified means	Male	10-14 years	95+ years	0	Population Density (150-300 ppl/sqkm, proportion)
Self-harm by other specified means	Male	10-14 years	95+ years	0	Population Density (300-500 ppl/sqkm, proportion)
Self-harm by other specified means	Male	10-14 years	95+ years	0	Population Density (500-1000 ppl/sqkm, proportion)
Self-harm by other specified means	Male	10-14 years	95+ years	0	Population Density (over 1000 ppl/sqkm, proportion)
Self-harm by other specified means	Male	10-14 years	95+ years	0	Population Density (under 150 ppl/sqkm, proportion)
Self-harm by other specified means	Male	10-14 years	95+ years	-1	Religion (binary, >50% Muslim)
Self-harm by other specified means	Male	10-14 years	95+ years	1	Log-transformed SEV scalar: Self Harm
Self-harm by other specified means	Male	10-14 years	95+ years	0	Socio-demographic Index
Self-harm by other specified means	Male	10-14 years	95+ years	1	Major depressive disorder
Self-harm by other specified means	Male	10-14 years	95+ years	-1	Healthcare access and quality index

SR Table 4. Total number of deaths, mortality rate per 100,000, total years of life lost (YLL) and YLL rate per 100,000 by sex and 5-year age group globally in 2016. Values in brackets are 95% uncertainty intervals.								
	**Deaths**				**YLLs**			
	**Number**		**Rate**		**Number**		**Rate**	
**Age (years)**	**Female**	**Male**	**Female**	**Male**	**Female**	**Male**	**Female**	**Male**
10 to 14	3443 (2935 to 3973)	4533 (3759 to 5226)	1.2 (1 to 1.3)	1.4 (1.2 to 1.7)	255420 (217752 to 294737)	335984 (278636 to 387405)	86.6 (73.8 to 99.9)	106.3 (88.2 to 122.6)
15 to 19	24269 (21474 to 27295)	25002 (22016 to 28007)	8.5 (7.5 to 9.6)	8.2 (7.2 to 9.2)	1680169 (1486670 to 1889675)	1729132 (1522633 to 1937196)	588.3 (520.5 to 661.7)	566.9 (499.2 to 635.1)
20 to 24	29624 (26668 to 32689)	50088 (44511 to 55363)	10.2 (9.2 to 11.2)	16.2 (14.4 to 17.9)	1907169 (1716876 to 2104513)	3221939 (2863325 to 3561319)	654.6 (589.3 to 722.3)	1041.6 (925.6 to 1151.3)
25 to 29	27970 (25456 to 30465)	57703 (51608 to 64771)	9.3 (8.5 to 10.2)	18.4 (16.4 to 20.6)	1662786 (1513252 to 1811177)	3428497 (3066460 to 3848383)	554.7 (504.8 to 604.2)	1092.5 (977.1 to 1226.3)
30 to 34	20843 (19138 to 22628)	54874 (47900 to 60578)	7.5 (6.9 to 8.2)	19.2 (16.8 to 21.2)	1135371 (1042462 to 1232644)	2988885 (2609217 to 3299609)	410.2 (376.6 to 445.3)	1047.2 (914.2 to 1156.1)
35 to 39	18293 (16984 to 19812)	49900 (42609 to 55900)	7.3 (6.8 to 8)	19.6 (16.7 to 21.9)	906004 (841153 to 981292)	2472196 (2110838 to 2769678)	363.8 (337.8 to 394)	970.2 (828.4 to 1086.9)
40 to 44	16722 (15727 to 17959)	47374 (41752 to 53739)	6.9 (6.5 to 7.5)	19.2 (17 to 21.8)	746156 (701748 to 801313)	2115160 (1864119 to 2399125)	310 (291.5 to 332.9)	859.1 (757.2 to 974.5)
45 to 49	17118 (16154 to 18148)	46515 (40804 to 52243)	7.5 (7 to 7.9)	20.1 (17.6 to 22.5)	681316 (642967 to 722299)	1852518 (1625060 to 2080605)	297.1 (280.4 to 315)	798.6 (700.6 to 897)
50 to 54	17409 (16538 to 18574)	44884 (39328 to 51620)	8.5 (8 to 9)	21.9 (19.2 to 25.2)	610887 (580310 to 651742)	1575656 (1380558 to 1812076)	296.7 (281.9 to 316.5)	768 (672.9 to 883.2)
55 to 59	15165 (14212 to 16252)	41246 (35534 to 47190)	8.6 (8.1 to 9.3)	24.1 (20.8 to 27.6)	461598 (432613 to 494703)	1256677 (1082671 to 1437837)	263.1 (246.6 to 282)	734.7 (633 to 840.6)
60 to 64	15273 (14164 to 16410)	35295 (30261 to 39672)	9.9 (9.2 to 10.7)	24.1 (20.7 to 27.1)	395425 (366725 to 424846)	915132 (784758 to 1028627)	256.8 (238.2 to 276)	625.4 (536.3 to 703)
65 to 69	13688 (12699 to 14778)	29275 (24383 to 33101)	11.6 (10.7 to 12.5)	27.2 (22.6 to 30.7)	293831 (272595 to 317253)	629630 (524501 to 711971)	248.7 (230.7 to 268.5)	584.8 (487.2 to 661.3)
70 to 74	11066 (10234 to 12025)	23192 (19363 to 26089)	13.2 (12.2 to 14.3)	31.7 (26.5 to 35.7)	190582 (176232 to 207083)	400500 (334311 to 450465)	226.6 (209.6 to 246.2)	547.4 (457 to 615.7)
75 to 79	10037 (9221 to 10952)	20561 (16831 to 22931)	15.4 (14.2 to 16.8)	40 (32.7 to 44.6)	133144 (122315 to 145263)	273970 (224236 to 305506)	204.3 (187.7 to 222.9)	533 (436.2 to 594.3)
80 to 84	7857 (7268 to 8474)	15650 (12786 to 17448)	18.6 (17.2 to 20.1)	51.6 (42.1 to 57.5)	76597 (70860 to 82607)	153499 (125320 to 171155)	181.4 (167.8 to 195.6)	505.8 (412.9 to 563.9)
85 to 89	5638 (5279 to 6070)	9477 (7752 to 10475)	22.4 (21 to 24.2)	64.4 (52.7 to 71.2)	38656 (36193 to 41621)	65477 (53547 to 72400)	153.9 (144.1 to 165.7)	444.9 (363.9 to 492)
90 to 94	2483 (2324 to 2665)	3309 (2651 to 3613)	24.3 (22.8 to 26.1)	72.6 (58.2 to 79.3)	11625 (10877 to 12476)	15631 (12518 to 17072)	113.9 (106.6 to 122.2)	342.9 (274.6 to 374.5)
95 plus	727 (677 to 779)	646 (532 to 711)	29.2 (27.2 to 31.3)	80.1 (65.9 to 88.1)	2193 (2043 to 2351)	2005 (1653 to 2204)	88.1 (82 to 94.4)	248.4 (204.7 to 273)

SR Table 5. List of 195 countries and territories and their corresponding 3 letter identifiers used in the GBD study

Location Name	ISO
Afghanistan	AFG
Albania	ALB
Algeria	DZA
American Samoa	ASM
Andorra	AND
Angola	AGO
Antigua and Barbuda	ATG
Argentina	ARG
Armenia	ARM
Australia	AUS
Austria	AUT
Azerbaijan	AZE
Bahrain	BHR
Bangladesh	BGD
Barbados	BRB
Belarus	BLR
Belgium	BEL
Belize	BLZ
Benin	BEN
Bermuda	BMU
Bhutan	BTN
Bolivia	BOL
Bosnia and Herzegovina	BIH
Botswana	BWA
Brazil	BRA
Brunei	BRN
Bulgaria	BGR
Burkina Faso	BFA
Burundi	BDI
Cambodia	KHM
Cameroon	CMR
Canada	CAN
Cape Verde	CPV
Central African Republic	CAF
Chad	TCD
Chile	CHL

SR Table 5. List of 195 countries and territories and their corresponding 3 letter identifiers used in the GBD study

China	CHN
Colombia	COL
Comoros	COM
Congo	COG
Costa Rica	CRI
Cote d'Ivoire	CIV
Croatia	HRV
Cuba	CUB
Cyprus	CYP
Czech Republic	CZE
Democratic Republic of the Congo	COD
Denmark	DNK
Djibouti	DJI
Dominica	DMA
Dominican Republic	DOM
Ecuador	ECU
Egypt	EGY
El Salvador	SLV
Equatorial Guinea	GNQ
Eritrea	ERI
Estonia	EST
Ethiopia	ETH
Federated States of Micronesia	FSM
Fiji	FJI
Finland	FIN
France	FRA
Gabon	GAB
Georgia	GEO
Germany	DEU
Ghana	GHA
Greece	GRC
Greenland	GRL
Grenada	GRD
Guam	GUM
Guatemala	GTM
Guinea	GIN
Guinea-Bissau	GNB

SR Table 5. List of 195 countries and territories and their corresponding 3 letter identifiers used in the GBD study

Guyana	GUY
Haiti	HTI
Honduras	HND
Hungary	HUN
Iceland	ISL
India	IND
Indonesia	IDN
Iran	IRN
Iraq	IRQ
Ireland	IRL
Israel	ISR
Italy	ITA
Jamaica	JAM
Japan	JPN
Jordan	JOR
Kazakhstan	KAZ
Kenya	KEN
Kiribati	KIR
Kuwait	KWT
Kyrgyzstan	KGZ
Laos	LAO
Latvia	LVA
Lebanon	LBN
Lesotho	LSO
Liberia	LBR
Libya	LBY
Lithuania	LTU
Luxembourg	LUX
Macedonia	MKD
Madagascar	MDG
Malawi	MWI
Malaysia	MYS
Maldives	MDV
Mali	MLI
Malta	MLT
Marshall Islands	MHL
Mauritania	MRT

SR Table 5. List of 195 countries and territories and their corresponding 3 letter identifiers used in the GBD study

Mauritius	MUS
Mexico	MEX
Moldova	MDA
Mongolia	MNG
Montenegro	MNE
Morocco	MAR
Mozambique	MOZ
Myanmar	MMR
Namibia	NAM
Nepal	NPL
Netherlands	NLD
New Zealand	NZL
Nicaragua	NIC
Niger	NER
Nigeria	NGA
North Korea	PRK
Northern Mariana Islands	MNP
Norway	NOR
Oman	OMN
Pakistan	PAK
Palestine	PSE
Panama	PAN
Papua New Guinea	PNG
Paraguay	PRY
Peru	PER
Philippines	PHL
Poland	POL
Portugal	PRT
Puerto Rico	PRI
Qatar	QAT
Romania	ROU
Russia	RUS
Rwanda	RWA
Saint Lucia	LCA
Saint Vincent and the Grenadines	VCT
Samoa	WSM
Sao Tome and Principe	STP

SR Table 5. List of 195 countries and territories and their corresponding 3 letter identifiers used in the GBD study

Saudi Arabia	SAU
Senegal	SEN
Serbia	SRB
Seychelles	SYC
Sierra Leone	SLE
Singapore	SGP
Slovakia	SVK
Slovenia	SVN
Solomon Islands	SLB
Somalia	SOM
South Africa	ZAF
South Korea	KOR
South Sudan	SSD
Spain	ESP
Sri Lanka	LKA
Sudan	SDN
Suriname	SUR
Swaziland	SWZ
Sweden	SWE
Switzerland	CHE
Syria	SYR
Taiwan	TWN
Tajikistan	TJK
Tanzania	TZA
Thailand	THA
The Bahamas	BHS
The Gambia	GMB
Timor-Leste	TLS
Togo	TGO
Tonga	TON
Trinidad and Tobago	TTO
Tunisia	TUN
Turkey	TUR
Turkmenistan	TKM
Uganda	UGA
Ukraine	UKR
United Arab Emirates	ARE

SR Table 5. List of 195 countries and territories and their corresponding 3 letter identifiers used in the GBD study

United Kingdom	GBR
United States	USA
Uruguay	URY
Uzbekistan	UZB
Vanuatu	VUT
Venezuela	VEN
Vietnam	VNM
Virgin Islands, U.S.	VIR
Yemen	YEM
Zambia	ZMB
Zimbabwe	ZWE
