# Supplementary material for: Global, regional, and national burden of suicide mortality 1990 to 2016: systematic analysis for the Global Burden of Disease Study 2016
Source: BMJ. 2019 Feb 6;364:l94. doi: 10.1136/bmj.l94 (PMC6598639; doi:10.1136/bmj.l94)
Supplement: Supplementary file 2 — Supplementary materials: Full author list and affiliations [file nagm045998.ww2.pdf]

Authors

Heather M Orpana, Adjunct Professor, Researcher[1,2;] Laurie B Marczak, Scientific Publications Manager[3]; Megha Arora, Publications Officer[3]; Nooshin Abbasi, Researcher[4]; Rizwan Suliankatchi Abdulkader, Lecturer[5]; Zegeye Abebe, Researcher[6]; Haftom Niguse Abraha, Lecturer, Clinical Pharmacist[7,8]; Mohsen Afarideh, Research Collaborator[9]; Mahdi Afshari, Associate Professor[10]; Alireza Ahmadi, Assistant Professor[11]; Amani Nidhal Aichour, Medical Student[12]; Ibtihel Aichour, Medical Student[12]; Miloud Taki Eddine Aichour, Veterinarian[13]; Nadia Akseer, Senior Research Associate[14]; Rajaa M Al-Raddadi, Assistant Professor[15]; Fares Alahdab, Assistant Professor[16]; Ala'a Alkerwi, Principal Investigator[17]; Peter Allebeck, Professor, Secretary General[18,19]; Nelson Alvis-Guzman, Professor Titular, Professor Titular[20,21]; Nahla Hamed Anber, Researcher, Biochemistry Fellow[22,23]; Mina Anjomshoa, PhD Student, Researcher[24,25]; Carl Abelardo T Antonio, Assistant Professor, PhD Student[26,27]; Amit Arora, Senior Lecturer[28,29]; Krishna K Aryal, Deputy Team Leader[30]; Solomon Weldegebreal Asgedom, Assistant Professor[31]; Ashish Awasthi, Assistant Professor, Assistant Professor[32,33]; Beatriz Paulina Ayala Quintanilla, Doctor, Researcher[34,35]; Hamid Badali, Associate Professor[36]; Suzanne Lyn Barker-Collo, Professor[37]; Till Winfried Bärnighausen, Director, Professor[38,39]; Shahrzad Bazargan-Hejazi, Professor, Adjunct Professor[40,41]; Corina Benjet, Researcher[41,42]; Isabela M Bensenor, Associate Professor[43]; Noami Berfeld, Research Assistant[44]; Mircea Beuran, Director[45]; Zulfiqar A Bhutta, Co-Director, Director[46,47]; Belete Biadgo, Assistant Professor[48]; Nigus Bililign, Lecturer[49]; Guilherme Borges, Researcher[50]; Rohan Borschmann, Senior Research Fellow, Senior Research Fellow[51,52]; Alexandra Brazinova, Associate Professor[53]; Nicholas J K Breitborde, Associate Professor, Associate Professor[54,55]; Traolach Brugha, Clinical Professor[56]; Zahid A Butt, Post-Doctoral Research Fellow, Visiting Associate Professor[57,58] ; Juan J Carrero, Associate Professor[59]; Félix Carvalho, Professor[60]; Deborah Carvalho Malta, Associate Professor[61]; Carlos A Castañeda-Orjuela, Director, PhD Student[62,63]; Ferrán Catalá-López, Senior scientist, Affiliate Investigator[64,65] ; Liliana G Ciobanu, PhD Student, Research Associate[66,67]; Berihun Assefa Dachew, Assistant Professor, PhD Student[68,69]; Lalit Dandona, Professor, Professor[34,3]; Rakhi Dandona, Professor, Clinical Professor[33,3]; Paul I Dargan, Consultant Physician, Professor of Clinical Toxicology[70,71]; Ahmad Daryani, Professor[72]; Dragos Virgil Davitoiu, Lecturer, Consultant Surgeon[73,74]; Kairat Davletov, Director[75]; Louisa Degenhardt, Professor, Affiliate Professor[77,3]; Gebre Teklemariam Demoz, Lecturer, Researcher[77,78]; Don C Des Jarlais, Professor[79]; Samath Dhamminda Dharmaratne, Associate Professor, Affiliate Assistant Professor[80,3]; Shirin Djalalinia, Assistant Professor[81]; Linh Doan, Researcher[82]; David Teye Doku, Senior Lecturer, Adjunct Professor[83,84]; Manisha Dubey, Food and Nutrition Security Analysis Expert[85]; Ziad El-Khatib, Associate Professor[86]; Sharareh Eskandarieh, Researcher[87]; Alireza Esteghamati, Professor[88]; Sadaf Esteghamati, Research Collaborator[89]; Andre Faro, Professor[90]; Farshad Farzadfar, Associate Professor[91]; Wubalem Fekadu, Assistant Professor, PhD student[92,93]; Eduarda Fernandes, Associate Professor[94]; Alize J Ferrari, Researcher[69,95]; Irina Filip, Adult Psychiatrist, Lecturer[96,97]; Florian Fischer, Assistant Professor[98]; Kyle J. Foreman, Director of Data Science[3]; Takeshi Fukumoto, Researcher, Doctor[99,100]; Abadi Kahsu Gebre, Lecturer[101]; Giuseppe Grosso, Researcher, Assistant Professor[102,103]; Rahul Gupta, Chief Health Officer/Commissioner of Public Health, Adjunct Professor[104,105]; Juanita A Haagsma, Assistant Professor[106]; Hassan Haghparast Bidgoli, Assistant Professor[107]; Arvin Haj-Mirzaian, Researcher, Doctor[108,109]; Samer Hamidi, Professor[110]; Graeme J Hankey, Professor, Consultant Neurologist[111,112]; Josep Maria Haro, Director, Associate Professor[113,114]; Hamid Yimam Hassen, Lecturer, Student[115,116]; Simon I Hay, Professor, Professor[3,117]; Behnam Heidari, Research Collaborator[88]; Delia Hendrie, Lecturer[118]; Enayatollah Homaie Rad, Researcher[119]; Seyed Mostafa Hosseini, Professor[120]; Sorin Hostiuc, Associate Professor, Assistant Professor[121,122]; Seyed Sina Naghibi Irvani, Researcher[91,123]; Sheikh

Mohammed Shariful Islam, Senior Research Fellow, Honorary Senior Lecturer[124,125]; Mihajlo Jakovljevic, Professor, Visiting Research Fellow[126,127]; Spencer James, Researcher[3]; Achala Upendra Jayatilleke, Lecturer, Lecturer[128,129]; Ravi Prakash Jha, PhD Student[130]; Jost B Jonas, Clinical Professor[131,132]; Jacek Jerzy Jozwiak, Professor[133]; Rajendra Kadel, Researcher[134]; Amaha Kahsay, Lecturer[135]; Amir Kasaeian, Assistant Professor of Biostatistics[136,137]; Getachew Mullu Kassa, Lecturer[138]; Norito Kawakami, Professor[139]; Adane Teshome Kefale, Lecturer[140]; Grant Rodgers Kemp, Editorial Coordinator[3]; Yousef Saleh Khader, Professor[141]; Morteza Abdullatif Khafaie, Associate Professor[142]; Ibrahim A Khalil, Senior Associate[3]; Ejaz Ahmad Khan, Associate Professor[143]; Muhammad Ali Khan, Assistant Professor, Research Fellow[144,145]; Muhammad Shahzeb Khan, Doctor, Researcher[146,147]; Young-Ho Khang, Director, Professor[148,149]; Jagdish Khubchandani, Associate Professor[150]; Aliasghar A Kiadaliri, Associate Researcher[151]; Christian Kieling, Associate Professor, Researcher[152,153]; Young-Eun Kim, Research Professor[154]; Adnan Kisa, Professor, Professor[155,156]; Ann Kristin Skrindo Knudsen, Researcher, Associate Professor[157,158]; Yoshihiro Kokubo, Doctor[159]; Ai Koyanagi, Researcher[114]; Varsha Sarah Krish, Data Analyst[3]; Barthelemy Kuate Defo, Professor, Professor[160,161]; G Anil Kumar, Researcher[162]; Manasi Kumar, Lecturer, Researcher[163,164]; Prabhat Lamichhane, Lecturer[165]; Justin J Lang, Researcher[166]; Arman Latifi, Assistant Professor[167]; Paul H Lee, Assistant Professor[168]; Janni Leung, Research Fellow[169]; Lee-Ling Lim, Lecturer, PhD Student[170,171]; Alan D Lopez, Professor[52,3;] Stefan Lorkowski, Professor, Professor[172,173]; Paulo A Lotufo, Clinical Professor[174]; Rafael Lozano, Professor[3,117]; Raimundas Lunevicius, Doctor[175]; P A Mahesh, Professor[176]; Marek Majdan, Associate Professor[177]; Reza Majdzadeh, Professor, Professor[178,179]; Reza Malekzadeh, Director, Professor[180,91;] Ana-Laura Manda, Doctor[181]; Mohammad Ali Mansournia, Associate Professor[182]; Lorenzo Giovanni Mantovani, Associate Professor[183]; Joemer C Maravilla, PhD Student[184]; Jose Martinez-Raga, Doctor[185,186]; Manu Raj Mathur, Associate Professor[162]; Pallab K Maulik, Director, Associate Professor[187,188]; John J McGrath, Professor[189,190]; Ravi Mehrotra, Clinical Professor[191]; Tesfa Mekonen, Assistant Professor[92]; Walter Mendoza, Program Analyst[192] ; Tuomo J Meretoja, Adjunct Professor[193,194]; Tomislav Mestrovic, Laboratory Director, Assistant Professor[195,196]; Ted R Miller, Researcher, Adjunct Professor[197,118;] GK Mini, Researcher, Associate Professor[198,199]; Erkin M Mirrakhimov, Clinical Professor, Doctor[200,201]; Philip B Mitchell, Professor[202]; Babak Moazen, PhD Student, Researcher[203]; Karzan Abdulmuhsin Mohammad, Lecturer[204,205]; Moslem Mohammadi, Associate Professor[206]; Shafiu Mohammed, Associate Professor, Research Associate[207,203;] Ali H Mokdad, Professor, Associate Chair for Collaboration[3,117]; Lorenzo Monasta, Researcher[208]; Mahmood Moosazadeh, Assistant Professor[209]; Ghobad Moradi, Associate Professor of Epidemiology[210,211]; Maziar Moradi-Lakeh, Professor[212]; Mehdi Moradinazar, Assistant Professor[213]; Ilais Moreno Velásquez, Researcher[214]; Naho Morisaki, Researcher[215]; Shane Douglas Morrison, Doctor[216]; Marilita M Moschos, Associate Professor[217,218]; Seyyed Meysam Mousavi, PhD Student[25]; Ghulam Mustafa, Assistant Professor, Chairperson[219,220]; Gabriele Nagel, Associate Professor[221]; Aliya Naheed, Associate Professor[222]; Gurudatta Naik, Researcher[223]; Farid Najafi, Professor[224]; Ionut Negoi, Doctor, Lecturer[45,181]; Ruxandra Irina Negoi, Associate Professor[225,226]; Huong Lan Thi Nguyen, Research Manager[227]; Long Hoang Nguyen, Director[228]; Molly R Nixon, Scientific Publications Manager[3]; Richard Ofori-Asenso, PhD Student[229,230]; Felix Akpojene Ogbo, Lecturer[231]; In-Hwan Oh, Associate Professor[232]; Andrew T Olagunju, Lecturer, PhD Student[233,234]; Tinuke O Olagunju, Graduate Student[235]; Simon Øverland, Director, Professor[236,158;] Mayowa Ojo Owolabi, Professor[237]; Songhomitra Panda-Jonas, Doctor[131]; Charles D H Parry, Director, Professor[238,239]; Sanghamitra Pati, Director[240]; Scott B Patten, Professor[241,242]; George C Patton, Professor, Researcher[243,244]; Max Petzold, Director[245]; Michael R Phillips, Professor[246]; Oleguer Plana-Ripoll, Researcher[190]; Maarten J Postma, Professor[247,248]; Akram

Pourshams, Clinical Professor[180]; Hossein Poustchi, Associate Professor[180]; Mostafa Qorbani, Assistant Professor[249]; Amir Radfar, Researcher, Board Member[250,251]; Anwar Rafay, Assistant Professor[252]; Alireza Rafiei, Professor of Immunology[253,254]; Fakher Rahim, Assistant Professor, Director[255,256]; Afarin Rahimi-Movaghar, Clinical Professor[257]; Vafa Rahimi-Movaghar, Clinical Professor[258]; Muhammad Aziz Rahman, Senior Lecturer[165,259]; Rajesh Kumar Rai, Senior Research Scientist, Research Associate[260,261]; Shahab Rezaeian, Assistant Professor[213]; Leonardo Roever, Clinical Epidemiologist[262]; Luca Ronfani, Medical Director[208]; Gholamreza Roshandel, Assistant Professor[180,263]; Ali Rostami, Assistant Professor[264]; Perminder S Sachdev, Professor, Director[202,265]; Hosein Safari, Assistant Professor[266]; Saeid Safiri, Assistant Professor[267]; Payman Salamati, Professor[258,268]; Yahya Salimi, Assistant Professor[269]; Joshua A Salomon, Professor[270]; Abdallah M Samy, Lecturer[271]; Itamar S Santos, Associate Professor[272]; Milena M Santric-Milicevic, Professor[273]; Benn Sartorius, Professor, Affiliate Professor[274,117;] Shahabeddin Sarvi, Associate Professor[72]; Maheswar Satpathy, Assistant Professor, Executive Director[275,276]; Monika Sawhney, Associate Professor[277]; David C Schwebel, Professor[278]; Sadaf G Sepanlou, Assistant Professor[91,180]; Masood Ali Shaikh, Independent Consultant[279]; Mehdi Sharif, Professor[280]; Kenji Shibuya, Director[281]; Mika Shigematsu, Researcher[282]; Rahman Shiri, Adjunct Professor[283]; Ivy Shiue, Researcher[284,285]; Soraya Siabani, Assistant Professor, Honorary Research Fellow[286,287]; Tariq J Siddiqi, Doctor[147]; Inga Dora Sigfusdottir, Professor, Professor[288,289]; João Pedro Silva, Research Associate[60]; Jasvinder A. Singh, Professor, Professor[223,290]; Adauto Martins Soares Filho, Epidemiologist, PhD Student[291,292]; Soheila Sobhani, Researcher[293]; Dan J Stein, Professor, Researcher[294,295]; Murray B Stein, Professor, Staff Psychiatrist[296,297]; Mu'awiyyah Babale Sufiyan, Lecturer, Consultant[298,299]; Bruno F Sunguya, Lecturer, Lecturer[300,301]; Rafael Tabarés-Seisdedos, Clinical Professor, Principal Investigator[185,302]; Karen M Tabb, Assistant Professor[303]; Mohammad Tavakkoli, Assistant Professor[80]; Arash Tehrani-Banihashemi, Assistant Professor, Vice Chancellor of Research[304,305]; Mohamad-Hani Temsah, Assistant Professor, Assistant Professor[306,307]; Roman Topor-Madry, Director, President[308,309]; Bach Xuan Tran, Associate Professor, Associate Professor[310,311]; Khanh Bao Tran, Researcher[312,313]; Irfan Ullah, Researcher, Assistant Professor[314,315]; Jurgen Unutzer, Professor, Adjunct Professor[316,317]; Muhammad Shariq Usman, Student[318]; Olalekan A Uthman, Associate Professor[319]; Pascual R Valdez, Adjunct Professor, Doctor[320,321]; Tommi Juhani Vasankari, Director[322]; Cintia Vasconcelos, Technologist[323]; Vasily Vlassov, Professor[324]; Theo Vos, Professor[3,117]; Isidora S Vujcic, Assistant Professor[325]; Yasir Waheed, Assistant Professor[326]; Yuan-Pang Wang, Associate Researcher[327]; Elisabete Weiderpass, Professor[328,329]; Andrea Werdecker, Researcher[330,331]; Ronny Westerman, Researcher[330]; Harvey A Whiteford, Professor, Affiliate Professor[332,333]; Grant M A Wyper, Researcher, PhD Student[334,335]; Mehdi Yaseri, Researcher[120,336]; Ebrahim M Yimer, Lecturer[337]; Engida Yisma, Lecturer[338]; Naohiro Yonemoto, Assistant Professor[339]; Seok-Jun Yoon, Doctor, Professor[154,340]; Marcel Yotebieng, Assistant Professor[341,342]; Mahmoud Yousefifard, Assistant Professor[343]; Chuanhua Yu, Adjunct Professor[344,345]; Zoubida Zaidi, Professor[346,347]; Mohammad Zamani, Medical Student[348]; ; Christopher J L Murray, Professor/Institute Director, Chair/Professor[3,117]; Mohsen Naghavi, Professor, Professor[3,117].

Affiliations

1 Applied Research Division, Public Health Agency of Canada, Ottawa, ON, Canada.

2 School of Psychology, University of Ottawa, ON, Canada.

3 Institute for Health Metrics and Evaluation, University of Washington, Seattle, WA, USA.

4 Montreal Neuroimaging Center, McGill University, Montreal, QC, Canada.

5 Department of Statistics, Manomaniam Sundaranar University, Tirunelveli, India.

6 Human Nutrition Department, University of Gondar, Gondar, Ethiopia.

7 Clinical Pharmacy Unit, Mekelle University, Mekelle, Ethiopia.

8 Ayder Comprehensive Specialized Hospital, Mekelle University, Mekelle, Ethiopia.

9 Endocrinology and Metabolism Research Center, Tehran University of Medical Sciences, Tehran, Iran.

10 Zabol University of Medical Sciences, Zabol, Iran.

11 Department of Anesthesiology, Kermanshah University of Medical Sciences, Kermanshah, Iran.

12 University Ferhat Abbas of Setif, Setif, Algeria.

13 Higher National School of Veterinary Medicine, Algiers, Algeria.

14 The Hospital for Sick Children, University of Toronto, Toronto, ON, Canada.

15 Department of Family and Community Medicine, King Abdulaziz University, Jeddah, Saudi Arabia.

16 Evidence Based Practice Center, Mayo Clinic Foundation for Medical Education and Research, Rochester, MN, USA.

17 Department of Population Health, Luxembourg Institute of Health, Strassen, Luxembourg.

18 Department of Public Health Sciences, Karolinska Institutet, Stockholm, Sweden.

19 Swedish Research Council for Health, Working Life, and Welfare, Stockholm, Sweden.

20 Research Group on Health Economics, University of Cartagena, Cartagena, Colombia.

21 Research Group in Hospital Management and Health Policies, University of the Coast, Barranquilla, Colombia.

22 Mansoura University, Mansoura, Egypt.

23 Faculty of Medicine, Mansoura University, Mamnsoura, Egypt.

24 Social Determinants of Health Research Center, Rafsanjan University of Medical Sciences, Rafsanjhan, Iran.

25 Department of Health Management and Economics, Tehran University of Medical Sciences, Tehran, Iran.

26 Department of Health Policy and Administration, University of the Philippines Manila, Manila, Philippines.

27 Department of Applied Social Sciences, Hong Kong Polytechnic University, Hong Kong, China.

28 School of Science and Health, Western Sydney University, Sydney, NSW, Australia.

29 Oral Health Services, Sydney Local Health District, Sydney, NSW, Australia.

30 Monitoring Evaluation and Operational Research Project, Abt Associates Nepal, Lalitpur, Nepal.

31 School of Pharmacy, Mekelle University, Mekelle, Ethiopia.

32 Indian Institute of Public Health, Gandhinagar, India.

33 Public Health Foundation of India, Gurugram, India.

34 The Judith Lumley Centre, La Trobe, University, Melbourne, VIC, Australia.

35 General Office for Research and Technological Transfer, Peruvian National Institute of Health, Lima, Peru.

36 Department of Medical Mycology, Mazandaran University of Medical Sciences, Sari, Iran.

37 School of Psychology, University of Auckland, Auckland, New Zealand.

38 Institute of Public Health, Heidelberg, University, Heidelberg, Germany.

39 Department of Global Health, Harvard University, Boston, MA, USA.

40 Department of Psychiatry, Charles R. Drew University of Medicine and Science, Los Angeles, CA, USA.

41 Department of Psychiatry and Biobehavioral Sciences, University of California Los Angeles, Los Angeles, CA, USA.

42 Department of Epidemiology and Psychosocial Research, Ramón de la Fuente Muñiz National Institute of Psychiatry, Mexico City, Mexico.

43 Department of Internal Medicine, University of São Paulo, São Paulo, Brazil.

44 Public Health Agency of Canada, Ottawa, ON, Canada.

45 Emergency Hospital of Bucharest, Carol Davila University of Medicine and Pharmacy, Bucharest, Romania.

46 The Hospital for Sick Children, University of Toronto, Toronto, ON, Canada.

47 Center of Excellence in Women and Child Health, Aga Khan University, Karachi, Pakistan.

48 Department of Clinical Chemistry, University of Gondar, Gondar, Ethiopia.

49 Department of Midwifery, Woldia University, Woldia, Ethiopia.

50 Department of Epidemiology and Psychosocial Research, Ramón de la Fuente Muñiz National Institute of Psychiatry, Mexico City, Mexico.

51 Centre for Adolescent Health, Murdoch Childrens Research Institute, Melbourne, VIC, Australia.

52 School of Population and Global Health, University of Melbourne, Melbourne, VIC, Australia.

53 Institute of Epidemiology, Comenius University, Bratislava, Slovakia.

54 Psychiatry and Behavioral Health Department, Ohio State University, Columbus, OH, USA.

55 Department of Psychology, Ohio State University, Columbus, OH, USA.

56 Department of Health Sciences, University of Leicester, Leicester, UK.

57 School of Population and Public Health, University of British Columbia, Vancouver, BC, Canada.

58 Al Shifa School of Public Health, Al Shifa Trust Eye Hospital, Rawalpindi, Pakistan.

59 Department of Medical Epidemiology and Biostatistics, Karolinska Institutet, Stockholm, Sweden.

60 Applied Molecular Biosciences Unit, University of Porto, Porto, Portugal.

61 Department of Maternal and Child Nursing and Public Health, Federal University of Minas Gerais, Belo Horizonte, Brazil.

62 Colombian National Health Observatory, National Institute of Health, Bogota, Colombia.

63 Epidemiology and Public Health Evaluation Group, National University of Colombia, Bogota, Colombia.

64 Department of Health Planning and Economics, Institute of Health Carlos III, Madrid, Spain.

65 Clinical Epidemiology Program, Ottawa Hospital Research Institute, Ottawa, ON, Canada.

66 Adelaide Medical School, University of Adelaide, Adelaide, SA, Australia.

67 School of Pharmacy and Medical Sciences, University of South Australia, Adelaide, SA, Australia.

68 Institute of Public Health, University of Gondar, Gondar, Ethiopia.

69 School of Public Health, The University of Queensland, Brisbane, QLD, Australia.

70 Clinical Toxicology Service, Guy's and St. Thomas' NHS Foundation Trust, London, UK.

71 Faculty of Life Sciences and Medicine, King's College London, London, UK.

72 Toxoplasmosis Research Center, Mazandaran University of Medical Sciences, Sari, Iran.

73 Department of General Surgery, Carol Davila University of Medicine and Pharmacy, Bucharest, Romania.

74 Department of Surgery, Clinical Emergency Hospital Sf. Pantelimon, Bucharest, Romania.

75 Department of Epidemiology, Kazakh National Medical University, Almaty, Kazakhstan.

76 National Drug and Alcohol Research Centre, University of New South Wales, Sydney, NSW, Australia.

77 Department of Clinical Pharmacy, Aksum University, Aksum, Ethiopia.

78 Addis Ababa University, Addis Ababa, Ethiopia.

79 Department of Psychiatry and Behavioral Sciences, New York Medical College, Valhalla, NY, USA.

80 Department of Community Medicine, University of Peradeniya, Peradeniya, Sri Lanka.

81 Deputy of Research and Technology, Ministry of Health and Medical Education, Tehran, Iran.

82 Center of Excellence in Health Service Management, Nguyen Tat Thanh University, Ho Chi Minh City, Vietnam.

83 Department of Population and Health, University of Cape Coast, Cape Coast, Ghana.

84 Faculty of Social Sciences, Health Sciences, University of Tampere, Tampere, Finland.

85 United Nations World Food Programme, New Delhi, India.

86 Department of Public Health Sciences, Karolinska Institutet, Stockholm, Sweden.

87 Multiple Sclerosis Research Center, Tehran University of Medical Sciences, Tehran, Iran.

88 Endocrinology and Metabolism Research Center, Tehran University of Medical Sciences, Tehran, Iran.

89 Endocrine Research Center, Tehran University of Medical Sciences, Tehran, Iran.

90 Department of Psychology, Federal University of Sergipe, Sao Cristovao, Brazil.

91 Non-communicable Diseases Research Center, Tehran University of Medical Sciences, Tehran, Iran.

92 Department of Psychiatry, Bahir Dar University, Bahir Dar, Ethiopia.

93 Department of Psychiatry, Addis Ababa University, Addis Ababa, Ethiopia.

94 REQUIMTE/LAQV, University of Porto, Porto, Portugal.

95 Queensland Centre for Mental Health Research, Brisbane, QLD, Australia.

96 Psychiatry Department, Kaiser Permanente, Fontana, CA, USA.

97 Department of Health Sciences, A.T. Still University, Mesa, AZ, USA.

98 Department of Public Health Medicine, Bielefeld University, Bielefeld, Germany.

99 Gene Expression & Regulation Program, Cancer Institute (W.I.A.), Philadelphia, PA, USA.

100 Department of Dermatology, Kobe University, Kobe, Japan.

101 School of Pharmacy, Mekelle University, Mekelle, Ethiopia.

102 Integrated Tumor Registry, Vittorio Emanuele University Hospital Polyclinic, Catania, Italy.

103 Department of Biomedical and Biotechnological Sciences, University of Catania, Catania, Italy.

104 West Virginia Bureau for Public Health, Charleston, WV, USA.

105 Department of Health Policy, Management & Leadership, West Virginia University, Morgantown, WV, USA.

106 Department of Public Health, Erasmus University Medical Center, Rotterdam, Netherlands.

107 Institute for Global Health, University College London, London, UK.

108 Department of Pharmacology, Tehran University of Medical Sciences, Tehran, Iran.

109 Obesity Research Center, Shahid Beheshti University of Medical Sciences, Tehran, Iran.

110 School of Health and Environmental Studies, Hamdan Bin Mohammed Smart University, Dubai, United Arab Emirates.

111 School of Medicine, University of Western Australia, Perth, WA, Australia.

112 Neurology Department, Sir Charles Gairdner Hospital, Perth, WA, Australia.

113 Biomedical Research Networking Center for Mental Health Network (CiberSAM), Madrid, Spain.

114 Research and Development Unit, San Juan de Dios Sanitary Park, Barcelona, Spain.

115 Public Health Department, Mizan-Tepi University, Teppi, Ethiopia.

116 Unit of Epidemiology and Social Medicine, University Hospital Antwerp, Wilrijk, Belgium.

117 Department of Health Metrics Sciences, University of Washington, Seattle, WA, USA.

118 School of Public Health, Curtin, Bentley, WA, Australia.

119 Guilan Road Trauma Research Center, Guilan University of Medical Sciences, Rasht, Iran.

120 Department of Epidemiology and Biostatistics, Tehran University of Medical Sciences, Tehran, Iran.

121 Department of Legal Medicine and Bioethics, Carol Davila University of Medicine and Pharmacy, Bucharest, Romania.

122 Clinical Legal Medicine, National Institute of Legal Medicine Mina Minovici, Bucharest, Romania.

123 Research Institute for Endocrine Sciences, Shahid Beheshti University of Medical Sciences, Tehran, Iran.

124 Institute for Physical Activity and Nutrition, Deakin University, Burwood, VIC, Australia.

125 Sydney Medical School, University of Sydney, Sydney, NSW, Australia.

126 Medical Sciences Department, University of Kragujevac, Kragujevac, Serbia.

127 Division of Health Economics, Lund University, Lund, Sweden.

128 Institute of Medicine, University of Colombo, Colombo, Sri Lanka.

129 Faculty of Graduate Studies, University of Colombo, Colombo, Sri Lanka.

130 Department of Community Medicine, Banaras Hindu University, Varanasi, India.

131 Department of Ophthalmology, Heidelberg University, Mannheim, Germany.

132 Beijing Institute of Ophthalmology, Beijing Tongren Hospital, Beijing, China.

133 Faculty of Medicine and Health Sciences, University of Opole, Opole, Poland.

134 Personal Social Services Research Unit, London School of Economics and Political Science, London, UK.

135 Department of Nutrition and Dietetics, Mekelle University, Mekelle, Ethiopia.

136 Hematology-Oncology and Stem Cell Transplantation Research Center, Tehran University of Medical Sciences, Tehran, Iran.

137 Hematologic Malignancies Research Center, Tehran University of Medical Sciences, Tehran, Iran.

138 College of Health Sciences, Debre Markos University, Debre Markos, Ethiopia.

139 Department of Mental Health, University of Tokyo, Tokyo, Japan.

140 Pharmacy Department, Mizan-Tepi University, Teppi, Ethiopia.

141 Department of Public Health and Community Medicine, Jordan University of Science and Technology, Ramtha, Jordan.

142 Department of Public Health, Ahvaz Jundishapur University of Medical Sciences, Ahvaz, Iran.

143 Epidemiology and Biostatistics Department, Health Services Academy, Islamabad, Pakistan.

144 Division of Gastroenterology & Hepatology, University of Alabama at Birmingham, Birmingham, Alabama.

145 University of Tennessee, Knoxville, Tennessee.

146 Department of Internal Medicine, John H. Stroger, Jr. Hospital of Cook County, Chicago, IL, USA.

147 Department of Internal Medicine, Dow University of Health Sciences, Karachi, Pakistan.

148 Department of Health Policy and Management, Seoul National University, Seoul, South Korea.

149 Institute of Health Policy and Management, Seoul National University, Seoul, South Korea.

150 Department of Nutrition and Health Sciences, Ball State University, Muncie, IN, USA.

151 Clinical Epidemiology Unit, Lund University, Lund, Sweden.

152 Department of Psychiatry, Federal University of Rio Grande do Sul, Porto Alegre, Brazil.

153 Child & Adolescent Psychiatry Division, Clinical Hospital, Porto Alegre, Brazil.

154 Department of Preventive Medicine, Korea University, Seoul, South Korea.

155 Department of Health Management and Health Economics, University of Oslo, Oslo, Norway.

156 Department of Global Community Health and Behavioral Sciences, Tulane University, New Orleans, LA, USA.

157 Center for Disease Burden, Norwegian Institute of Public Health, Bergen, Norway.

158 Department of Psychosocial Science, University of Bergen, Bergen, Norway.

159 Department of Preventive Cardiology, National Cerebral and Cardiovascular Center, Suita, Japan.

160 Department of Demography, University of Montreal, Montreal, QC, Canada.

161 Department of Social and Preventive Medicine, University of Montreal, Montreal, QC, Canada.

162 Public Health Foundation of India, Gurugram, India.

163 Department of Psychiatry, University of Nairobi, Nairobi, Kenya.

164 Department of Psychology, University College London, London, UK.

165 School of Medicine, Deakin University, Geelong, VIC, Australia.

166 Health Promotion and Chronic Disease Prevention Branch, Public Health Agency of Canada, Ottawa, ON, Canada.

167 Department of Public Health, Maragheh University of Medical Sciences, Maragheh, Iran.

168 School of Nursing, Hong Kong Polytechnic University, Hong Kong, China.

169 Center for Youth Substance Abuse Research, The University of Queensland, Brisbane, QLD, Australia.

170 Department of Medicine, Department of Medicine, Kuala Lumpur, Malaysia.

171 Department of Medicine and Therapeutics, The Chinese University of Hong Kong, Hong Kong, China.

172 Institute of Nutrition, Friedrick Schiller University Jena, Jena, Germany.

173 Competence Cluster for Nutrition and Cardiovascular Health (nutriCARD), Jena, Germany.

174 Department of Medicine, University of São Paulo, São Paulo, Brazil.

175 Department of General Surgery, Aintree University Hospital NHS Foundation Trust, Liverpool, UK.

176 Department of Pulmonary Medicine, JSS Academy of Higher Education and Research, Mysore, India.

177 Department of Public Health, Trnava University, Trnava, Slovakia.

178 Community-Based Participatory-Research (CBPR) Center, Tehran University of Medical Sciences, Tehran, Iran.

179 Knowledge Utilization Research Center (KURC), Tehran University of Medical Sciences, Tehran, Iran.

180 Digestive Diseases Research Institute, Tehran University of Medical Sciences, Tehran, Iran.

181 Surgery Department, Emergency Hospital of Bucharest, Carol Davila University of Medicine and Pharmacy, Bucharest, Romania.

182 Department of Epidemiology, Tehran University of Medical Sciences, Tehran, Iran.

183 School of Medicine and Surgery, University of Milan Bicocca, Monza, Italy.

184 Institute for Social Science Research, The University of Queensland, Brisbane, Australia.

185 Department of Medicine, University of Valencia, Valencia, Spain.

186 Psychiatry Department, University Hospital Doctor Peset, Valencia, Spain.

187 Research Department, The George Institute for Global Health, New Delhi, India.

188 School of Medicine, University of New South Wales, Sydney, NSW, Australia.

189 Queensland Brain Institute, The University of Queensland, Brisbane, QLD, Australia.

190 National Centre for Register-based Research, Aarhus University, Aarhus, Denmark.

191 Preventive Oncology, National Institute of Cancer Prevention and Research, Noida, India.

192 Peru Country Office, United Nations Population Fund, Lima, Peru.

193 Breast Surgery Unit, Helsinki University Hospital, Helsinki, Finland.

194 University of Helsinki, Helsinki, Finland.

195 Clinical Microbiology and Parasitology Unit, Dr. Zora Profozic Polyclinic, Zagreb, Croatia.

196 University Centre Varazdin, University North, Varazdin, Croatia.

197 Pacific Institute for Research & Evaluation, Calverton, MD, USA.

198 Achutha Menon Centre for Health Science Studies, Sree Chitra Tirunal Institute for Medical Sciences and Technology, Trivandrum, India.

199 Global Institute of Public Health, Ananthapuri Hospitals and Research Institute, Trivandrum, India.

200 Faculty of General Medicine, Kyrgyz State Medical Academy, Bishkek, Kyrgyzstan.

201 Department of Atherosclerosis and Coronary Heart Disease, National Center of Cardiology and Internal Disease, Bishkek, Kyrgyzstan.

202 School of Psychiatry, University of New South Wales, Sydney, NSW, Australia.

203 Institute of Public Health, Heidelberg, University, Heidelberg, Germany.

204 Department of Biology, Salahaddin University, Erbil, Iraq.

205 ISHIK University, Erbil, Iraq.

206 Department of Physiology and Pharmacology, Mazandaran University of Medical Sciences, Sari, Iran.

207 Health Systems and Policy Research Unit, Ahmadu Bello University, Zaria, Nigeria.

208 Clinical Epidemiology and Public Health Research Unit, Burlo Garofolo Institute for Maternal and Child Health, Trieste, Italy.

209 Health Sciences Research Center, Mazandaran University of Medical Sciences, Sari, Iran.

210 Social Determinants of Health Research, Kurdistan University of Medical Sciences, Sanandaj, Iran.

211 Department of Epidemiology and Biostatistics, Kurdistan University of Medical Sciences, Sanandaj, Iran.

212 Preventive Medicine and Public Health Research Center, Iran University of Medical Sciences, Tehran, Iran.

213 Research Center for Environmental Determinants of Health, Kermanshah University of Medical Sciences, Kermanshah, Iran.

214 Gorgas Memorial Institute for Health Studies, Panama City, Panama.

215 Department of Social Medicine, National Center for Child Health and Development, Setagaya, Japan.

216 Department of Surgery, University of Washington, Seattle, WA, USA.

217 Department of Ophthalmology, University of Athens, Athens, Greece.

218 Biomedical Research Foundation, Academy of Athens, Athens, Greece.

219 Pediatrics Department, Nishtar Medical University, Multan, Pakistan.

220 Pediatrics & Pediatric Pulmonology, Institute of Mother & Child Care, Multan, Pakistan.

221 Institute of Epidemiology and Medical Biometry, Ulm University, Ulm, Germany.

222 Initiative for Non Communicable Diseases, International Centre for Diarrhoeal Disease Research, Dhaka, Bangladesh.

223 Department of Epidemiology, University of Alabama at Birmingham, Birmingham, AL, USA.

224 Department of Epidemiology & Biostatistics, Kermanshah University of Medical Sciences, Kermanshah, Iran.

225 Anatomy and Embryology Department, Carol Davila University of Medicine and Pharmacy, Bucharest, Romania.

226 Department of Cardiology, Cardio-Aid, Bucharest, Romania.

227 Institute for Global Health Innovations, Duy Tan University, Hanoi, Vietnam.

228 Center for Excellence in Behavioral Health, Nguyen Tat Thanh University, Ho Chi Minh City, Vietnam.

229 Centre of Cardiovascular Research and Education in Therapeutics, Monash University, Melbourne, VIC, Australia.

230 Independent Consultant, Accra, Ghana.

231 Translational Health Research Institute, Western Sydney University, Penrith, NSW, Australia.

232 Department of Preventive Medicine, Kyung Hee University, Seoul, South Korea.

233 Department of Psychiatry, University of Lagos, Lagos, Nigeria.

234 Department of Psychiatry, University of Adelaide, Adelaide, SA, Australia.

235 Department of Pathology and Molecular Medicine, McMaster University, Hamilton, ON, Canada.

236 Division of Mental and Physical Health, Norwegian Institute of Public Health, Bergen, Norway.

237 Institute for Advanced Medical Research and Training, University of Ibadan, Ibadan, Nigeria.

238 Alcohol, Tobacco, & Other Drug Use Research Unit, Medical Research Council South Africa, Cape Town, South Africa.

239 Department of Psychiatry, Stellenbosch University, Cape Town, South Africa.

240 Regional Medical Research Centre, Indian Council of Medical Research, Bhubaneswar, India.

241 Community Health Sciences, University of Calgary, Calgary, AB, Canada.

242 Department of Psychiatry, University of Calgary, Calgary, AB, Canada.

243 Department of Paediatrics, University of Melbourne, Melbourne, VIC, Australia.

244 Population Health Group, Murdoch Childrens Research Institute, Melbourne, VIC, Australia.

245 Swedish National Data Service, Gothenburg, Sweden.

246 Shanghai Mental Health Center, Shanghai Jiao Tong University, Shanghai, China.

247 Department of Pharmacy, University of Groningen, Groningen, Netherlands.

248 Department of Economics and Business, University of Groningen, Groningen, Netherlands.

249 Non-communicable Diseases Research Center, Alborz University of Medical Sciences, Karaj, Iran.

250 A.T. Still University, Mesa, AZ, USA.

251 Medichem, Barcelona, Spain.

252 Department of Epidemiology & Biostatistics, Contech School of Public Health, Lahore, Pakistan.

253 Department of Immunology, Mazandaran University of Medical Sciences, Sari, Iran.

254 Molecular and Cell Biology Research Center, Mazandaran University of Medical Sciences, Sari, Iran.

255 Thalassemia and Hemoglobinopathy Research Center, Health Research Institute, Ahvaz, Iran.

256 Endocrinology and Metabolism Molecular-Cellular Sciences Institute, Metabolomics and Genomics Research Center, Tehran, Iran.

257 Iranian National Center for Addiction Studies (INCAS), Tehran University of Medical Sciences, Tehran, Iran.

258 Sina Trauma and Surgery Research Center, Tehran University of Medical Sciences, Tehran, Iran.

259 Austin Clinical School of Nursing, La Trobe University, Heidelberg, VIC, Australia.

260 Society for Health and Demographic Surveillance, Suri, India.

261 Department of Economics, University of Goettingen, Göttingen, Germany.

262 Department of Clinical Research, Federal University of Uberlândia, Uberlândia, Brazil.

263 Golestan Research Center of Gastroenterology and Hepatology, Golestan University of Medical Sciences, Gorgan, Iran.

264 Infectious Diseases and Tropical Medicine Research Center, Babol University of Medical Sciences, Babol, Iran.

265 Neuropsychiatric Institute, Prince of Wales Hospital, Randwick, Australia.

266 Department of Neurosurgery, Ahvaz Jundishapur University of Medical Sciences, Ahvaz, Iran.

267 Managerial Epidemiology Research Center, Maragheh University of Medical Sciences, Maragheh, Iran.

268 School of Health and Policy Management, York University, Toronto, ON, Canada.

269 Social Development and Health Promotion Research Center, Kermanshah University of Medical Sciences, Kermanshah, Iran.

270 Center for Health Policy & Center for Primary Care and Outcomes Research, Stanford University, Stanford, CA, USA.

271 Department of Entomology, Ain Shams University, Cairo, Egypt.

272 Department of Internal Medicine, University of São Paulo, São Paulo, Brazil.

273 Centre School of Public Health and Health Management, University of Belgrade, Belgrade, Serbia.

274 Department of Public Health Medicine, University of KwaZulu-Natal, Durban, South Africa.

275 UGC Centre of Advanced Study in Psychology, Utkal University, Bhubaneswar, India.

276 Udyam-Global Association for Sustainable Development, Bhubaneswar, India.

277 Department of Public Health Sciences, University of North Carolina at Charlotte, Charlotte, NC, USA.

278 Department of Psychology, University of Alabama at Birmingham, Birmingham, AL, USA.

279 Independent Consultant, Karachi, Pakistan.

280 Department of Basic Sciences, Islamic Azad University, Sari, Iran.

281 Department of Global Health Policy, University of Tokyo, Tokyo, Japan.

282 National Institute of Infectious Diseases, Tokyo, Japan.

283 Finnish Institute of Occupational Health, Helsinki, Finland.

284 Institute of Medical Epidemiology, Martin Luther University Halle-Wittenberg, Halle, Germany.

285 Alzheimer Scotland Dementia Research Centre, University of Edinburgh, Edinburgh, Scotland.

286 Imam Ali Cardiovascular Research Center, Kermanshah University of Medical Sciences, Kermanshah, Iran.

287 School of Health, University of Technology Sydney, Sydney, NSW, Australia.

288 Department of Psychology, Reykjavik University, Reykjavik, Iceland.

289 Department of Health and Behavior Studies, Columbia University, New York City, NY, USA.

290 Department of Medicine, University of Alabama at Birmingham, Birmingham, AL, USA.

291 Department of Diseases and Noncommunicable Diseases and Health Promotion, Federal Ministry of Health, Brasilia, Brazil.

292 Collective Health, University of Brasilia, Brasilia, Brazil.

293 Students' Scientific Research Center, Tehran University of Medical Sciences, Tehran, Iran.

294 Department of Psychiatry and Mental Health, University of Cape Town, Cape Town, South Africa.

295 South African Medical Research Council, Cape Town, South Africa.

296 Departments of Psychiatry and Family Medicine & Public Health, University of California San Diego, La Jolla, CA, USA.

297 VA San Diego Healthcare System, San Diego, CA, USA.

298 Community Medicine Department, Ahmadu Bello University, Zaria, Nigeria.

299 Community Medicine, Ahmadu Bello University Teaching Hospital, Zaria, Nigeria.

300 Department of Community Health, Muhimbili University of Health and Allied Sciences, Dar es Salaam, Tanzania.

301 Implementation Science Tanzania, Muhimbili University of Health and Allied Sciences, Dar es Salaam, Tanzania.

302 Carlos III Health Institute, Biomedical Research Networking Center for Mental Health Network (CiberSAM), Madrid, Spain.

303 School of Social Work, University of Illinois, Urbana, IL, USA.

304 Preventive Medicine and Public Health Research Center, Iran University of Medical Sciences, Tehran, Iran.

305 Department of Community Medicine, Iran University of Medical Sciences, Tehran, Iran.

306 Department of Pediatrics, King Saud University, Riyadh, Saudi Arabia.

307 College of Medicine, Alfaisal University, Riyadh, Saudi Arabia.

308 Institute of Public Health, Jagiellonian University Medical College, Krakow, Poland.

309 Agency for Health Technology Assessment and Tariff System, Warszawa, Poland.

310 Department of Health Economics, Hanoi Medical University, Hanoi, Vietnam.

311 Bloomberg School of Public Health, Johns Hopkins University, Baltimore, MD, USA.

312 Molecular Medicine and Pathology, University of Auckland, Auckland, New Zealand.

313 Clinical Hematology and Toxicology, Military Medical University, Hanoi, Vietnam.

314 TB Culture Laboratory, Mufti Mehmood Memorial Teaching Hospital, Dera Ismail Khan, Pakistan.

315 Gomal Center of Biochemistry and Biotechnology, Gomal University, Dera Ismail Khan, Pakistan.

316 Department of Psychiatry and Behavioral Sciences, University of Washington, Seattle, WA, USA.

317 Health Services and Global Health, University of Washington, Seattle, WA, Washington.

318 Department of Internal Medicine, Dow University of Health Sciences, Karachi, Pakistan.

319 Division of Health Sciences, University of Warwick, Coventry, UK.

320 Argentine Society of Medicine, Buenos Aires, Argentina.

321 Velez Sarsfield Hospital, Buenos Aires, Argentina.

322 UKK Institute, Tampere, Finland.

323 Department of Surveillance of Noncommunicable Diseases and Diseases and Health Promotion, Ministry of Health, Brasilia, Brazil.

324 Department of Health Care Administration and Economy, National Research University Higher School of Economics, Moscow, Russia.

325 Institute of Epidemiology, University of Belgrade, Belgrade, Serbia.

326 Foundation University Medical College, Foundation University, Rawalpindi, Pakistan.

327 Department of Psychiatry, University of São Paulo, São Paulo, Brazil.

328 Department of Research, Cancer Registry of Norway Institute for Population Based Cancer Research, Oslo, Norway.

329 Department of Medical Epidemiology and Biostatistics, Karolinska Institute, Stockholm, Sweden.

330 Competence Center Mortality-Follow-Up, Federal Institute for Population Research, Wiesbaden, Germany.

331 Independent Consultant, Staufenberg, Germany.

332 Population Mental Health, The University of Queensland, Brisbane, QLD, Australia.

333 Institute for Health Metrics and Evaluation, University of Washington, Seattle, WA, USA.

334 Information Services Division, National Health Service Scotland, Edinburgh, UK.

335 Institute of Pharmacy and Biomedical Sciences, University of Strathclyde, Glasgow, UK.

336 Ophthalmic Research Center, Shahid Beheshti University of Medical Sciences, Tehran, Iran.

337 Department of Pharmacology and Toxicology, Mekelle University, Mekelle, Ethiopia.

338 Department of Nursing and Midwifery, Addis Ababa University, Addis Ababa, Ethiopia.

339 Department of Psychopharmacology, National Center of Neurology and Psychiatry, Tokyo, Japan.

340 The National Mental Health and Welfare Commission, Seoul, South Korea.

341 Division of Epidemiology, Ohio State University, Columbus, OH, USA.

342 School of Public Health, University of Kinshasa, Kinshasa, Democratic Republic of the Congo.

343 Physiology Research Center, Iran University of Medical Sciences, Tehran, Iran.

344 Department of Epidemiology and Biostatistics, Wuhan University, Wuhan, China.

345 Global Health Institute, Wuhan University, Wuhan, China.

346 Department of Epidemiology, University Hospital of Setif, Setif, Algeria.

347 Department of General Medicine, University Ferhat Abbas of Setif, Setif, Algeria.

348 Student Research Committee, Babol University of Medical Sciences, Babol, Iran.
